# ﻿Taxonomic revalidation of *Selenobrachys* Schmidt, 1999 and *Chilocosmia* Schmidt & von Wirth, 1992 based on morphological and molecular analyses (Araneae, Theraphosidae), with the description of a new species from Romblon Island, Philippines

**DOI:** 10.3897/zookeys.1233.128056

**Published:** 2025-03-31

**Authors:** Darrell C. Acuña, Maria Mikaela U. Dumbrique, Maricel C. Ranido, Lorenz Rhuel P. Ragasa, Charles Nylxon C. Noriega, Anna Beatriz R. Mayor, Gregorio Antonio Florendo Jr, Mary Jane A. Fadri, Volker von Wirth, Myla R. Santiago-Bautista, Leonardo A. Guevarra Jr

**Affiliations:** 1 Research Center for Natural and Applied Sciences, University of Santo Tomas, Sampaloc, Manila 1008, Philippines; 2 Philippine Arachnological Society, Inc., Paco, Manila 1007, Philippines; 3 Graduate School, Polytechnic University of the Philippines, Sta. Mesa, Manila 1016, Philippines; 4 Department of Biochemistry, Faculty of Pharmacy, University of Santo Tomas, Sampaloc, Manila 1008, Philippines; 5 Romblon State University, Odiongan, Romblon 5505, Philippines; 6 Theraphosid Research Team, Hofmarkstr. 6, Eitting 85462, Germany

**Keywords:** Asian tarantulas, biogeography, *Orphnaecus*, Philippine spiders, phylogeny, Selenocosmiina

## Abstract

*Selenobrachys* Schmidt, 1999 and *Chilocosmia* Schmidt & von Wirth, 1992 were considered junior synonyms to *Orphnaecus* Simon, 1892 without further morphological investigation nor the use of molecular methods of analysis. Herein, the type specimens are reexamined with newly collected samples of currently known *Orphnaecus* species, including new specimens from Romblon Island, Philippines. Morphological and molecular analyses were performed, utilizing cytochrome oxidase I (COI) and ribosomal genes (12S–tRNA-Val–16S). Synapomorphies in the structure of maxillary lyra, spermathecae, and male palpal morphology were observed in *O.philippinus* and the Romblon specimen which are distinct from other *Orphnaecus* species. In addition, lyrate morphology, setae structure on the patella of palp dorsal, and the male palpal organ morphology of *O.dichromatus* differ from other *Orphnaecus* species. Cladistic separation observed in molecular phylogenetic analyses supports morphological observations. Our findings suggest that the genus *Selenobrachys* is distinct from *Orphnaecus*; hence, the genus *Selenobrachys* Schmidt, 1999, **stat. rev.** and its type species *Selenobrachysphilippinus* Schmidt, 1999, **comb. rest.**, are restored and the new species from Romblon Island, *Selenobrachysustromsupasius***sp. nov.**, be identified as the second *Selenobrachys* species. Furthermore, the genus *Chilocosmia* Schmidt & von Wirth, 1992, **stat. rev.** and the original combination of its type species, *Chilocosmiadichromata* Schmidt & von Wirth, 1992, **comb. rest.** are restored. Male specimens of *S.philippinus* and *C.dichromata* were described for the first time. Insights on the biogeography of Philippine tarantulas are discussed.

## ﻿Introduction

Tarantulas of the family Theraphosidae Thorell, 1869, are large-sized spiders that currently comprise 168 genera and over 1000 species (World Spider Catalog 2024). These spiders currently have colonized every continent on Earth, except Antarctica, and have a diverse array of terrestrial, burrowing, and arboreal species that exhibit unique defense mechanisms, predatory behaviors, reproductive strategies, and ecological adaptations (Pérez-Miles 2020; WSC 2024).

*Orphnaecus* Simon, 1892 is one of the 11 recognized tarantula genera of the subfamily Selenocosmiinae Simon, 1899 found in Asia and Australia (following the transfer of *Poecilotheria* Simon, 1885 from Selenocosmiinae to the revalidated subfamily Poecilotheriinae Simon, 1892 (Lüddecke et al. 2018; Foley et al. 2019). This genus currently has five accepted species (WSC 2024), which include *O.adamsoni*Salamanes et al., 2022, *O.dichromatus*, *O.kwebaburdeos* (Barrion-Dupo et al., 2015), *O.pellitus* Simon, 1892, and *O.philippinus* (Schmidt, 1999). Aside from *O.dichromatus*, which was discovered in Western New Guinea, Indonesia, the rest of the *Orphnaecus* species are endemic to the Philippines (Schmidt and von Wirth 1992; Nunn et al. 2016).

Simon (1892) distinguished the genus *Orphnaecus* in having smaller and more separated eyes. West et al. (2012) noted the unique structure of the maxillary lyra of *Orphnaecus* by its patch of similar short bacilliform rods in reniform shape and with rows of large clavate bacillae. Nunn et al. (2016) transferred *Phlogielluskwebaburdeos* to *Orphnaecus* by its lyrate morphology description that obviously fits the genus. Recently, Salamanes et al. (2022) described a new species, *O.adamsoni*, from the Dinagat Islands, Philippines.

Two species of *Orphnaecus*, namely *O.philippinus* and *O.dichromatus*, which are currently known only from female specimens, became part of this genus after *Selenobrachys* and *Chilocosmia* were synonymized with *Orphnaecus* (West et al. 2012). *Selenobrachysphilippinus* was transferred to *Orphnaecus* despite its structural difference in lyrate morphology (West et al. 2012). This species has an oval with a proximally truncate and distally tapering lyrate patch with rows of strong paddles, which is atypical of the characteristic reniform shape with rows of clavate bacillae generally found in *Orphnaecus* species (Schmidt 1999; West et al. 2012; Nunn et al. 2016). This characteristic of *O.philippinus* was considered, particularly the variation in lyrate morphology, as an autapomorphy, disregarding the recognition of *Selenobrachys* as a separate genus (West et al. 2012). *Chilocosmiadichromata* was transferred to *Selenocosmia* Ausserer, 1871 after the synonymy of *Chilocosmia* to *Selenocosmia* due to the lack of comparison which can adequately support the recognition of the genus (Raven 2000). Later, *Chilocosmia* was synonymized with *Orphnaecus* based on its lyrate morphology, median carapace line, spermathecal shape, male embolus keel, and cheliceral striker morphology (West et al. 2012). West et al. (2012) transferred only the type species, *C.dichromata*, to *Orphnaecus* and the other species formerly placed in *Chilocosmia* were continued to be treated as *Selenocosmia* (*Se.arndsti*, *Se.barensteinerae*, *Se.peerboomi*, and *Se.samarae*) (WSC 2024). Schmidt (2015) disagreed with this and suggested that genetic investigations be made to confirm the validity of the revision.

Molecular biology methods such as DNA barcoding and sequence analysis are techniques that have become useful in identifying organisms and resolving issues related to taxonomic identity (Hebert et al. 2003a, 2003b, 2004, 2016; Hebert and Gregory 2005; Antil et al. 2022). DNA barcoding has been a complementary method that is used as a rapid method to identify organisms and resolve taxonomic ambiguities (Tyagi et al. 2019). This technique allows rapid identification of organisms at the species level based on the sequences of genes with a length of between 400 and 800 base pairs (Kress and Erickson 2008). Typically, the mitochondrial markers cytochrome oxidase I (COI) and 16S rRNA are the genes used in DNA barcoding for animals (Amer 2021). The ultimate objective of this technique is to produce and report a DNA sequence that represents an organism in a global database and use this as taxonomic information for comparison with unknown organisms for identification (Hebert et al. 2003a, 2003b, 2004, 2016; Hebert and Gregory 2005; Wilson et al. 2018).

In this study, we restored two genera: *Chilocosmia* stat. rev. with type species *C.dichromata* comb. rest. and *Selenobrachys* stat. rev. with type species *S.philippinus* comb. rest. A new *Selenobrachys* species collected from Romblon Island, Philippines is described. Males of *C.dichromata* comb. rest. and *S.philippinus* comb. rest. are described for the first time. Also, we utilized the concept of Pleistocene Aggregate Island Complexes (PAICs), which identify the seven major biogeographical regions of the Philippines, to discuss our insights on the biogeography of Philippine theraphosid fauna.

## ﻿Materials and methods

### ﻿Specimen collection

The newly collected specimens used in this study were collected through opportunistic sampling, mostly after midday to night, from different site localities of tarantula spiders in the Philippines. Specimens of *O.dichromatus* examined were from the Staatliches Museum für Naturkunde Stuttgart collection in Germany. For field collection, permits and consent were acquired from respective local government units and Protected Area Management Bureau (PAMB). Gratuitous permit was secured from the Department of Environment and Natural Resources- Biodiversity Management Bureau (DENR-BMB) prior to sampling. Newly collected specimens were preserved in 80% ethanol.

### ﻿Species concept

The unified species concept proposed by de Queiroz (2005, 2007) was followed, which utilizes pluralistic evidence and a pragmatic approach. The best available evidence we have for species delimitation used in this study is morphological and genetic criteria. Species delimitation was conducted using morphological differences and then verified using genetic data based on the result of phylogenetic analysis.

The undescribed putative species used in the phylogenetic analysis were morphospecies initially identified based on their distribution and morphological differences, such as in lyra, genitalia, legs, and setation. They are denoted by their island locality and species number (e.g., “L1” = “Luzon Island species 1”).

### ﻿Morphological analysis

The descriptive format generally follows West et al. (2012). Species and genera described in this study are diagnosed and compared using type specimens and newly collected samples. Observations and documentation were made using an Olympus SZ61 stereomicroscope with a Touptek camera attachment or with a 3.0 USB C-mount Touptek microscope camera adapted with a Nikon SMZ 18 stereo microscope. Illumination was a Starlight RL 5 ring light (daylight) and a Starlight IL 11 incident light (pure white). Images were stacked with Helicon Focus 8.2.0 and post-processed with Adobe Photoshop CS2 9.0. Micro measurements were measured using ToupView software version X64, and macro measurements were taken using digital calipers. All measurements are given in millimeters to the nearest 0.01 mm. Measurement of total body length includes chelicerae but does not include spinnerets. Carapace length is measured from the anterior tip to the posterior longest point. Cephalic height is measured from the base of the carapace to the highest point of the cephalic region laterally. The cheliceral strikers are counted and categorized into three rows: the primary rows are the strikers found at the lowest rows which are distinctly longer, secondary rows are in the middle rows which are often shorter than the primary strikers, and the tertiary rows are above the secondary rows which are the very tiny strikers. Pseudostrikers are rows of long and pallid setae found below the cheliceral strikers. The length of leg segments was measured on the lateral aspect up to the longest point and did not include trochanter and coxa. Leg width is measured through the widest point and includes both lateral and dorsal aspects. The length of the tarsus does not include claws and tufts. The width of the tarsus and metatarsus does not include scopulae. The length and width of the coxae and trochanter were measured ventrally. The leg formula is the order of legs based on leg length given in descending order. The orientation of the eye rows is described by connecting the center or midpoint of each eye. Eye diameter is measured through the widest point or the major axis. The length of curve structures (e.g., fangs, claws, etc.) is measured by their arc/curve length. Fovea width and curve length were both measured. Palpal bulb structure followed Bertani (2000) based on the position of the keels. Measurements of tegulum and embolus followed Bertani (2023). Spermathecae are cleaned using lactic acid, and emboli are bleached using hydrogen peroxide (see von Wirth and Hildebrandt 2022, 2023). Variation in measurements, if available, is provided as ‘total sample size: minimum–maximum’ (n: min–max). Indices herein were created to ease comparative morphometrics between theraphosid species for future referencing.

### ﻿Indices used herein

**CI** Carapace Index = Car. width/ Car. length × 100, the resulting value shows the ratio of carapace width to length

**CLI** Cephalic Region Length Index = Cephalic region length/ Car. length × 100, the resulting value shows the length ratio of the cephalic region within the carapace

**CHI** Cephalic Region Height Index = Car. height/ Car. length × 100, the resulting value shows the ratio of the cephalic height to carapace length

**EI** Eye Index: EI(AME) = AME diameter/Car. length × 100; EI(ALE) = ALE diameter/Car. Length × 100; the resulting value shows the diameter ratio of AME/ALE to carapace length

**DLI** Dorsal Leg Index = Leg dor. width/ Leg length × 100, the resulting value shows the ratio of dorsal width to length of a leg

**LLI** Lateral Leg Index = Leg lat. width/ Leg length × 100, the resulting value shows the ratio of lateral width to the length of a leg

**RF**~ Leg Relation Factor (von Wirth and Striffler 2005) = Leg I length/Leg IV length × 100, the resulting value shows the length ratio of Leg I to Leg IV

**MI** Metatarsal Index = Met. length / Tib. length × 100, the resulting value shows the length ratio of the metatarsus to the tibia of the same leg

**TI** Tarsal Index = Tar. length/ Met. length × 100, the resulting value shows the length ratio of tarsus to metatarsus of the same leg

**EMI** Embolic Index = Embolus length/ tegulum length × 100, the resulting value shows the length ratio of the male embolus to its tegulum

**POI** Palpal Organ Index = (Embolus + tegulum length)/ Palp tib. length × 100, the resulting value shows the length ratio of the male palpal organ to the palpal tibia.

Setation followed Guadanucci et al. (2020) with additional terminologies. Terminologies on setation used in this study herein are designated as:

**TS** Tactile setae: hard mechanosensory setae for touch perception, which are the most common body sensilla of spiders (Guadanucci et al. 2020) and are diverse in form

**SC** Cuticular scales: flattened lanceolate or acicular (some are wavy and cotton-like) light-reflective covering setae or setal mat that have weak pedicel, almost parallel to the cuticle (Townsend and Felgenhauer 1998; Guadanucci et al. 2020)

**ETB** Epitrichobothria: tactile-like setae but very short, intermix with trichobothria on clusters (Guadanucci et al. 2020)

**TB** Trichobothria: clavate or filiform, ground or air movement sensitive sensilla (Guadanucci et al. 2020)

**CHS** Chemosensory sensilla: tiny translucent erect sensilla tapering distally (Guadanucci et al. 2020), usually found singly on the legs and abdomen

**FS** Femoral Setation: a field of modified tactile setae (TS) on the prolateral surface of femora I which varies in form (e.g., sword-like, acicular, filiform, etc.), that can be diagnostic in identifying selenocosmiine genera

**PB** Palpal Brush (first mentioned in West et al. 2012): a layer of modified elongated flat scales (SC) that is present on the male palpal patella and tibia dorsally; long and dense in *Orphnaecus*. They are classified as scales herein due to their weak pedicel and scale properties.

### ﻿Abbreviations and acronyms

**car.** carapace; **dor.** dorsal; **lat.** lateral; **fem.** femur; **met.** metatarsus; **OT** ocular tubercle; **tib.** tibia; **troch.** trochanter; **PS** prolateral superior keel; **PI** prolateral inferior keel; **A** apical keel; **BL** basal lobe; **Op** embolic opening; **StR** subtegular ridge; **AME** anterior median eye; **ALE** anterior lateral eye; **PME** posterior median eye; **PLE** posterior lateral eye; **PMS** posterior median spinneret; **PLS** posterior lateral spinneret; **MNHN**Muséum National d’Histoire Naturelle, Paris; **PASI** Philippine Arachnological Society, Inc. Reference Collection, Manila; **PNM**Philippine National Museum, Manila; **SMF**Senckenberg Museum, Frankfurt am Main; **SMNS**Staatliches Museum für Naturkunde, Stuttgart; **UPLB-MNH**University of the Philippines Los Baños - Museum of Natural History, Laguna; **UST-ARC** University of Santo Tomas - Arachnid Reference Collection, Manila; **ZMB**Museum für Naturkunde, Berlin; **PAIC** Pleistocene Aggregated Island Complex.

### ﻿DNA Isolation, amplification, and analysis

DNA samples were isolated from tissue collected from the right leg III of the tarantulas. DNA extraction was performed using the QIAGEN DNeasy Blood and Tissue Kit following the manufacturer’s protocol. For amplification of the Cytochrome oxidase I (COI) gene, degenerate primer pairs LCO1490_PH (forward)-HCO2198_PH (reverse) and C1-J- 2123-PH (forward)-C1-N-2776-PH (reverse) were used after modifications from the reference primers (see Table 1). The PCR mix consisted of a final concentration of 0.4uM each of forward and reverse primer, < 10 ng/μL DNA, 12.5ul 2X GoTaq G2 Master Mix, and water for a final volume of 25 μL. PCR conditions included an initial denaturation at 94 °C for 3 min, followed by five cycles consisting of denaturation at 94 °C for 30 s, annealing at 45 °C for 30 s, extension at 72 °C for 1 min, 35 cycles of denaturation at 94 °C for 30 s, annealing at 51 °C for 1 min, extension at 72 °C for 1 min, and a final extension at 72 °C for 10 min.

**Table 1.** List of primers used to amplify and sequence the DNA barcoding regions COI and rRNA genes.

Primers	Primer sequence	Source
**COI**		
LCO1490_PH (forward)	5’-TTTCWACTAATCATARGGATATTGG-3’	modified from LCO1490aphonopelma (Hamilton et al. 2011)
HCO2198_PH (reverse)	5’-TAAACCTCCGGATGWCCAAAAAAYCA-3’	modified from dgHCO-2198 (Meyer 2003)
C1-J-2123_PH (forward)	5’- GATCGAAATTTTAATACTTCKTTYTTTGA-3’	modified from C1-J-2123 (Vidergar et al. 2014)
C1-N-2776_PH (reverse)	5’-GGATAATCAGAATATCGTCGAGGTATTCCAT-3’	modified from C1-N-2776 (Hedin and Maddison 2001)
**12S–tRNA-Val–16S**		
12SUST_PHTarantula (forward)	5’-CGTCACCCTCGTCCAAAGAT-3’	this study
16SUST_PHTarantula (reverse)	5’-CGATAGGGTCTTGTCGTCCC-3’	this study

Amplification of the ribosomal genes, a continuous stretch of partial 12S, complete tRNA- Val, and partial 16S (12S–tRNA-Val–16S), was performed using our newly designed primer pair 12SUST-PHTarantula (forward)-16SUST-PHTarantula (reverse) (Table 1). PCR mix content is the same as with the COI primers above. PCR was performed with initial denaturation at 94 °C for 1 min, followed by 25 cycles consisting of denaturation at 94 °C for 1 min, annealing at 68 °C for 30 s, and extension at 72 °C for 1 min. This was followed by five cycles of denaturation at 94 °C for 1 min, annealing at 55 °C for 30 s, and extension at 72 °C for 1 min. The final extension step was performed at 72 °C for 10 min.

After PCR amplification, the products were separated and visualized on a 1.2% agarose gel with a 1kb DNA ladder. The resulting amplicons were sent for sequencing by Macrogen (Seoul, Republic of Korea). Sequence data was processed using PREGAP4 and GAP4 (Staden Package, http://staden.sourceforge.net/; Bonfield et al. 1995). Raw sequences were trimmed based on base quality. Forward and reverse reads were aligned and manually checked for ambiguous sites. A consensus sequence was then generated for each individual. Sequences from all COI primer pairs were combined to generate the COI consensus sequence for each individual for an extended length. Sequences were aligned with Theraphosidae sequences from databases using the MUSCLE algorithm. The hypervariable regions in rRNA gene alignment were removed using GBLOCKS v. 0.91b online tool (Castresana 2000) under all of the less stringent set of conditions. The rRNA genes were not separately analyzed due to the short lengths of 12S and tRNA-Val genes after cleaning.

Maximum-likelihood trees with 10,000 bootstrap replicates were generated in MEGA11 for both markers using the General Time Reversible model with gamma-distributed rates among sites with invariant sites (GTR+G+I), chosen in MEGA11 as best models for both markers based on the lowest Bayesian Information Criterion (BIC) score (Tamura et al. 2021). All sequences were used in generating the group (genus rank) mean and pairwise genetic distances using the Maximum Composite Likelihood model (Tamura et al. 2004) in MEGA11. Percent similarity is computed by getting the difference of 100% and the percent distance (similarity% = 100% - distance%). The two markers are not concatenated due to the unsuccessful ribosomal gene sequencing for many of the samples and many of the outgroups do not have corresponding COI or ribosomal gene sequences, hence analyzed separately. DNA sequences of outgroups were acquired from GenBank. The new sequences in this study are deposited to GenBank (Suppl. material 1).

Accession numbers of the sequences obtained from GenBank: **COI**— • JN018124 = *Aphonopelmaseemanni*MNHN-JAC96 • JN018125 = *Chilobrachyshuahini*MNHN-JAC48 • JF884459 = *Chilobrachys* sp. 2GAB_PAK (iBOL) • KJ744742 = *Selenotholus* sp. MYG381-T113579 • KT022078 = *Grammostola* sp. JEA-2015 • KT995328 = *Brachypelmaverdezi* IBUNAM:CNNA Ar003417 • MF804598 = *Chilobrachys* sp. USNM ENT 01117339 • MK234708 = *Grammostolarosea* NCA 2017/394 ; **12S–tRNA-Val–16S**— • MG273466 = *Brachypelma* sp. ZSM-A 2017/0058 • MG273467 = *Chilobrachysfimbriatus* ZSM-A 2017/0059 • MG273468 = *Cyriopagopuslividus* ZSM-A 2017/0060 • MG273470 = *Lampropelmanigerrimum* ZSM-A 2017/0063 • MG273471 = *Orphnaeus* [*Orphnaecus*] sp. ZSM-A 2017/0064 • MG273476 = *Lyrognathusgiannisposatoi* ZSM-A 2017/0070 • MG273477 = *Phlogiellus* sp. [?] ZSM-A 2017/0071 • MG273480 = *Selenocosmiajavanensis* ZSM-A 2017/0074 • MG273484 = *Poecilotheriaformosa* PONAL1 • MG273485 = *Grammostolapulchripes* GRGOL1.

Materials of subject species described in this study are placed on their respective taxonomic treatments (see Results).

### ﻿Comparative materials examined

#### ﻿Type materials

• *Orphnaecusadamsoni*Salamanes et. al, 2022: Philippines: Dinagat Island— **Dinagat Islands Prov.** • holotype ♂, PNM 14889; Loreto, Mt. Mangkuno; Oct 2018, J Santos, GG Villancio leg., forest grounds • allotype ♀, PNM 14888 [Ornithoctoninae sp.*]; Basilisa-Cagdianao, Mt. Arayat; Oct 2018, J Santos, GG Villancio leg.; PNM

*Remarks: The allotype ♀ (PNM 14888) described in Salamanes et al. (2022) and herein examined was misidentified and misplaced in *O.phnaecus.* It belongs to Ornithoctoninae due to the presence of plumose setal field on retrolateral chelicerae and has a stridulatory organ with rows of thorn setae on the prolateral maxilla, which characteristics are absent in Selenocosmiinae but synapomorphy to Ornithoctoninae. A taxon cannot be assigned for this specimen due to its poor condition.

• *Orphnaecuskwebaburdeos* (Barrion-Dupo et al., 2015): Philippines: Polillo Island—**Quezon Prov.** • paratypes, 3 ♂♂, BPB 2112012-4, BPB 2112012-12, BPB 2112012-13 and 1 ♀, BPB 2112012-2; Burdeos, [Brgy. Aluyon], Puting Bato Cave 3–4; 02 Nov 2012, J. Rasalan leg.; UPLB-MNH

• *Orphnaecuspellitus* Simon, 1892: Philippines: Luzon Island— **Camarines Sur Prov.** • syntypes ♂♀, AR4678; Libmanan, Calapnitan Caves [Culapnitan Caves] (now Libmanan Caves National Park); MNHN

#### ﻿Other materials

• *Orphnaecuskwebaburdeos* (Barrion-Dupo et al., 2015): Philippines: Polillo Island— **Quezon Prov.** • 4 ♂♂ and 14 ♀♀, 7 j, UST- ARC 0059–0083; Burdeos, Brgy. Aluyon, inside Puting Bato Cave 2 and 3; 150 m horiz. depth, 13–14 May 2023, DC Acuña, JD Fornillos leg.; UST-ARC

• *Orphnaecuspellitus* Simon, 1892: Philippines: Luzon Island— **Camarines Sur Prov.** • 2 ♂♂, 3 ♀♀, 12 j, UST-ARC 0031–0047; Libmanan, Brgy. Sigamot, Libmanan Caves National Park (Culapnitan Caves), inside Kalangkawan Cave; 50–300 m horiz. depth, 20 Apr 2023, LA Guevarra, DC Acuña, CN Noriega, JD Fornillos leg. • 4 j, UST-ARC 0048– 0051; [same general locality data as above], inside Alinsanay Cave; 50 m horiz. depth, [same collection data as above] • 2 ♂♂, 4 ♀♀, 1 j, UST-ARC 0052–0058; [same general locality data as above], inside Laya Cave; 30–50 m horiz. depth, 20 Jul 2023, LA Guevarra, DC Acuña, JD Fornillos leg.; UST-ARC

• *Orphnaecus* sp. ‘C1’: Philippines: Catanduanes Island— **Catanduanes Prov.** • 4 ♀♀, 1 j, PNM 18829–18833; Aug 1990, P Gonzales et al. leg.; PNM

• *Orphnaecus* sp. ‘L1’: Philippines: Luzon Island— **Laguna-Quezon Prov.** border • 1 ♂, 7 ♀♀, 1 j, UST-ARC 0105–0111; UP Sierra Madre Land Grant; 450 m a.s.l., burrows under rocks and logs, 19 Aug 2023, LA Guevarra, DC Acuña, CN Noriega, JD Fornillos, EP Maglangit leg. — **Laguna Prov.** • 1 j, UST-ARC 0084; Siniloan, Brgy. Magsaysay, Tulay na Bato Falls trail; 330 m a.s.l., burrows under logs, 04 Feb 2023, LA Guevarra, DC Acuña, CN Noriega, JD Fornillos leg.; UST-ARC

• *Orphnaecus* sp. ‘L2’: Philippines: Luzon Island— **Laguna Prov.** • 5 ♀♀, 9 j, UST- ARC 0088–0101; Siniloan, Brgy. Halayhayin; 15–405 m a.s.l., 05 Feb 2023, LA Guevarra, DC Acuña, CN Noriega, JD Fornillos leg. • 3 j, UST-ARC 0085–0087; Brgy. Magsaysay-Galalan, Southern Sierra Madre; 470 m a.s.l., under logs, 04 Feb 2023, LA Guevarra, DC Acuña, CN Noriega, JD Fornillos leg. — **Quezon Prov.** • 3 ♀♀, UST-ARC 0102–0104; Real, Brgy. Llavac; 300 m a.s.l., 14 May 2023; DC Acuña, CN Noriega, JD Fornillos leg.; UST-ARC

• *Orphnaecus* sp. ‘L3’: Philippines: Luzon Island— **Camarines Sur Prov.** • ♂♀ UST-ARC 0132–0133; Libmanan, Brgy. Sigamot, Libmanan Caves National Park; burrows under and inside logs on forest slope near Kalangkawan Cave, 20 July 2023, LA Guevarra, DC Acuña, JD Fornillos leg. • ♂♀ UST-ARC 0130–0131; [same locality and natural history data as above]; 20 Apr 2023, LA Guevarra, DC Acuña, CN Noriega, JD Fornillos leg. • ♀ UST-ARC 0134; Libmanan, Brgy. Malinao; burrow under limestone rock, 20 Apr 2023, LA Guevarra, DC Acuña, CN Noriega, JD Fornillos leg. • 3 ♂♂, 4 ♀♀, UST-ARC 0135–0139; [same locality data as the latter]; 20 Jul 2023, burrows under piles of coconut husks, LA Guevarra, DC Acuña, JD Fornillos leg.; UST-ARC

• *Orphnaecus* sp. ‘L4’: Philippines: Luzon Island— **Camarines Sur Prov.** • 4 ♀♀, UST-ARC 0141–0144; Libmanan, Brgy. Sigamot, Libmanan Caves National Park; burrows under logs on forest slope near Laya Cave, 20 July 2023, LA Guevarra, DC Acuña, JD Fornillos leg.; UST-ARC

• *Orphnaecus* sp. ‘L5’: Philippines: Luzon Island— **Nueva Ecija Prov.** • ♀, UST- ARC 0145; Bongabon; 2019, G Bathan leg. —**Pangasinan Prov.** • 3 ♂♂, 3 ♀♀, PASI ara0014–ara0019; Calasiao, Brgy. Nalsian, Sitio Centro; neglected residential lot and mango orchard, burrows under leaf litter, 08 Nov 2022, DOR Mapile leg.; UST-ARC/ PASI

• *Orphnaecus* sp. ‘L6’: Philippines: Luzon Island— **Pangasinan Prov.** • ♀ PASI- 0020; Sison, Brgy. Poblacion Sur; residential lot, burrows under rock, 08 Apr 2023, DC Acuña leg.; PASI

• *Orphnaecus* sp. ‘M1’: Philippines: Mindanao Island— **Agusan del Sur Prov.** • ♀, UST-ARC 0146; Prosperidad, Brgy. Poblacion, Puting Buhangin Cave- level 3; 2019, GD Petros leg.; UST-ARC

• *Orphnaecus* sp.: Philippines: Luzon Island—**Metro Manila** • ♀, ZMB 32341; Manila; Scheteley leg.; ZMB

## ﻿Results

### ﻿DNA sequence and phylogenetic analysis

**Figure 1.**
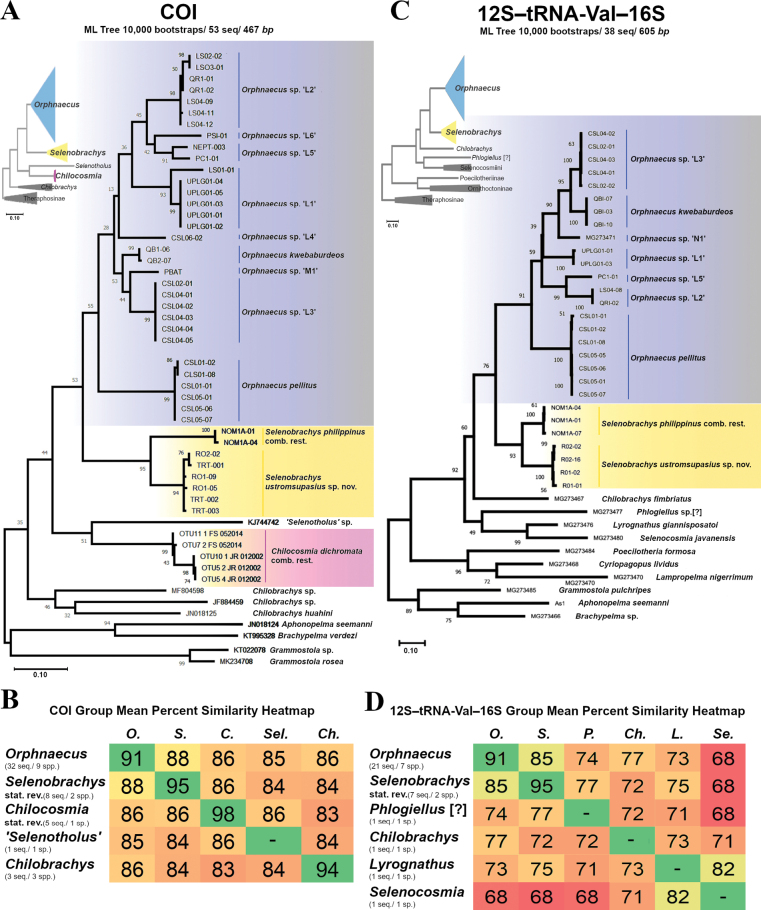
Maximum-Likelihood phylogenetic tree of **A**COI gene **C** rRNA genes (12S–tRNA-Val–16S). Group mean percent similarity heatmap of **B**COI gene **D** rRNA genes (12S–tRNA-Val–16S), using the Maximum Composite Likelihood model. Abbreviations: *O. = Orphnaecus*, *S. = Selenobrachys*, *C. = Chilocosmia*, *Ch. = Chilobrachys*, *P. = Phlogiellus*, *Se. = Selenocosmia*, *Se..* = *Selenotholus.* Note: dashes (-) in B and D denote values that are not computed because they only contain one gene sequence.

A total of 56 individuals were sampled which resulted in 45 new COI sequences and 28 new 12S–tRNA-Val–16S sequences. Additional 8 COI sequences and 10 12S–tRNA-Val–16S sequences were obtained from GenBank (Lüddecke et al. 2018). After alignment, a total of 53 sequences with 467 base pairs were used for COI, and 38 sequences with 605 base pairs were used for 12S–tRNA-Val–16S. The sequences under investigation include representatives from different genera, including *Orphnaecus*, *Selenobrachys* stat. rev., *Chilocosmia* stat. rev., and outgroups (Suppl. material 1). The difference between *Orphnaecus*, *Selenobrachys* stat. rev., and *Chilocosmia* stat. rev. is evident in genetic similarity matrices as well as their distinct placements in the phylogenetic tree (Fig. 1).

### ﻿Intergeneric genetic relationship

The phylogenetic tree topologies of both COI and ribosomal genes (12S–tRNA-Val–16S) depict the segregation of *Orphnaecus*, *Selenobrachys*, *Chilocosmia* into different clades (Fig. 1A, C). In the rRNA phylogenetic tree, *Orphnaecus*, *Selenobrachys*, Selenocosmiini genera (*Lyrognathus* and *Selenocosmia*), and Ornithoctoninae are among the clades that have moderate to good support, with bs = 76–99 (Fig. 1C). However, it is noticeable that all clade separations in the COI phylogenetic tree (Fig. 1A) have very low support (except Theraphosinae outgroups) despite having a good alignment during the analysis. This gene probably cannot resolve cladistic distinctions for these taxa or might need more gene samples to have better support to clades. The separation of *Orphnaecus* and *Selenobrachys* in the rRNA phylogenetic tree has moderate support with bs = 76. On the other hand, this separation is well supported by the genetic similarity matrices, indicating substantial genetic divergence between *Orphnaecus* and *Selenobrachys* clades, with an average of 88% similarity in COI and an average of 85% similarity in rRNA genes (Fig. 1B, D). The percent similarity of the two genera using both markers shows their close affinity suggesting that they have common ancestry and that *Selenobrachys* might have recently diverged into a distinct clade. Percent pairwise distance among individual samples between *Orphnaecus* and *Selenobrachys* ranges from 10.20%–15.50% in COI and from 12.05%–18.33% in rRNA genes (Suppl. material 2). *Chilocosmia* is more distant at a lower average similarity value of 86% to both *Orphnaecus* and *Selenobrachys*, as well as to *Selenotholus*, using the COI marker (rRNA gene sequences for *Chilocosmia* are not available) (Fig. 1B, D). Pairwise percent distances among individual samples of *Chilocosmia* from *Orphnaecus* individuals using the COI gene range from 10.59%–16.76%, and from *Selenobrachys* samples range from 11.81%–18.11% (Suppl. material 2).

### ﻿Interspecific genetic relationship

The percent similarity values between putative 9 species (only 7 putative species were successfully sequenced for rRNA genes) within *Orphnaecus* range from 89.14%–95.10% (at 4.9%–10.86% distance) in COI sequences and 86.46%–95.02% (at 4.98%–13.54% distance) in 12S–tRNA-Val–16S sequences using Maximum Composite Likelihood model (Tamura et al. 2004) (Suppl. material 2). Some species within *Orphnaecus* have relatively high pairwise distance values for COI. For instance, between *O.pellitus* (the type species) and the clade of the putative species *Orphnaecus* spp. ‘L1’, ‘L2’, ‘L5’, and ‘L6’ have 9.63%–10.86% pairwise distance (Suppl. material 2). Some species of this group have a closer genetic distance from other *Orphnaecus* species which does not support separation from *Orphnaecus* (Suppl. material 2). On the other hand, *O.kwebaburdeos* and *Orphnaecus* sp. ‘L3’ recorded the closest genetic affinity having a 4.90%–5.09% distance in COI, but heterospecificity is well supported by their distinct genital structures. In the rRNA genes, the furthest distance recorded with 12.03%–13.54% distance is between *O.pellitus* and *Orphnaecus* spp. ‘L2’, and ‘L3’ (‘L6’ has no successful sequence), except for *Orphnaecus* sp. ‘L1’ which has 8.47%–9.46% distance to *O.pellitus.* The closest distance is also between *O.kwebaburdeos* and *Orphnaecus* sp. ‘L3’ with 4.98% distance in all rRNA gene sequences.

The close relationship between the two *Selenobrachys* species, *S.philippinus* comb. rest. and *S.ustromsupasius* sp. nov., is evident in both the COI and rRNA genes (12S–tRNA- Val–16S) genetic matrices (Suppl. material 2). In the phylogenetic tree, their relationship has enough support in COI (bs = 95) and rRNA genes (bs = 93) (Fig. 1A, C). In the COI matrix, these two species exhibit percent similarity values of 90.57%–91.74% (8.43%–9.26% distance), while in the rRNA genes matrix, they show values of 90.18%–90.84% (9.16%–9.82% distance) (Suppl. material 2). These higher similarity values compared to other sequences suggest a relatively reduced genetic divergence between them. The phylogenetic tree placement further corroborates this observation. The COI sequences of *S.philippinus* comb. rest. and *S.ustromsupasius* sp. nov. cluster together within a distinct clade, suggesting a shared evolutionary history.

### ﻿Taxonomy

#### ﻿Family Theraphosidae Thorell, 1869

**Subfamily Selenocosmiinae Simon, 1889**

##### ﻿Tribe Selenocosmiini

Taxon classificationAnimaliaAraneaeTheraphosidae

Simon 1889

CB65A79A-D5E1-538C-A80D-4D054E879EEF

Phlogiini Simon, 1892 (synonymized by Simon 1903).

###### Included genera.

*Chilocosmia* Schmidt & von Wirth, 1992 stat. rev. (provisionally placed), *Coremiocnemis* Simon, 1892, *Lyrognathus* Pocock, 1895, *Psednocnemis* West, Nunn & Hogg, 2012, *Selenocosmia* Ausserer, 1871.

###### Remarks.

The genus *Chilocosmia* does not fit to any of the three currently recognized selenocosmiine tribes (Chilobrachini, Selenocosmiini, and Yamiini) and probably belongs to a tribe currently in synonymy with Selenocosmiini (probably Phlogiini Simon, 1892); hence, we provisionally placed it in Selenocosmiini until further investigation is conducted on the proper systematic placement of most of Papuan and Australian taxa.

##### ﻿Genus Chilocosmia

Taxon classificationAnimaliaAraneaeTheraphosidae

Schmidt & von Wirth, 1992 stat. rev.

BB3E3276-0712-5932-9FBA-544D5093C466

###### Type and included species.

*C.dichromata* Schmidt & von Wirth, 1992, comb. rest., by original designation and monotypy.

###### Diagnosis.

*Chilocosmia* stat. rev. differs from all known selenocosmiine genera (i) in having a palpal organ with twisted tegulum (Figs 4A–C, 5A–D) and (ii) in having a stridulatory organ on the prolateral maxilla with short bacilliform rods that form an arcuate strip of a lyrate patch and with club-shaped bacillae at lowest row (Fig. 3D, E). The lyra of *Chilocosmia* stat. rev. (Fig. 3D, E) quite resembles the lyra of *Orphnaecus* (Fig. 20G) but differs in the shape of the largest stridulating setae which lacks a pointed apex (Fig. 20C, F). It further differs from *Orphnaecus* in the male palpal organ morphology in having a twisted tegulum and a thicker embolus with shorter PS and lacking a basal lobe (Fig. 5A–E), and in lacking palpal brush on dorsal palp in males (Fig. 4D).

###### Distribution.

Indonesia: West New Guinea.

###### Etymology.

A combination of two Greek words *chilos* (*cheilos*; χείλος), which means lip, and *kosmein* (κοσμείν), which means arrange or keep in order (Schmidt and von Wirth 1992). Gender is feminine.

##### ﻿ Chilocosmia dichromata

Taxon classificationAnimaliaAraneaeTheraphosidae

Schmidt & von Wirth, 1992, comb. rest.

3645FB48-6D42-5FD8-8A11-CF8B66665EAA

**Figure 2.**
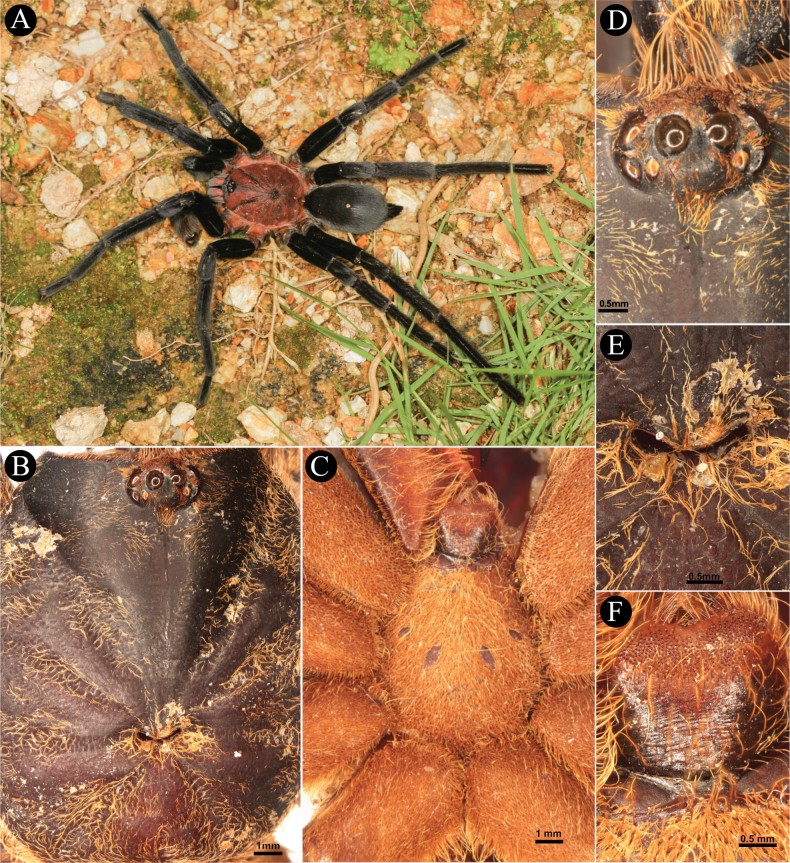
*Chilocosmiadichromata* comb. rest. ♂, SMNS Aran-004182 **A** habitus, in situ **B** prosoma, dorsal view **C** ventral view **D** ocular tubercle, dorsal view **E** fovea, dorsal view **F** labium, ventral view. Photo credit: Frank Schneider (Fig. 2A).

**Figure 3.**
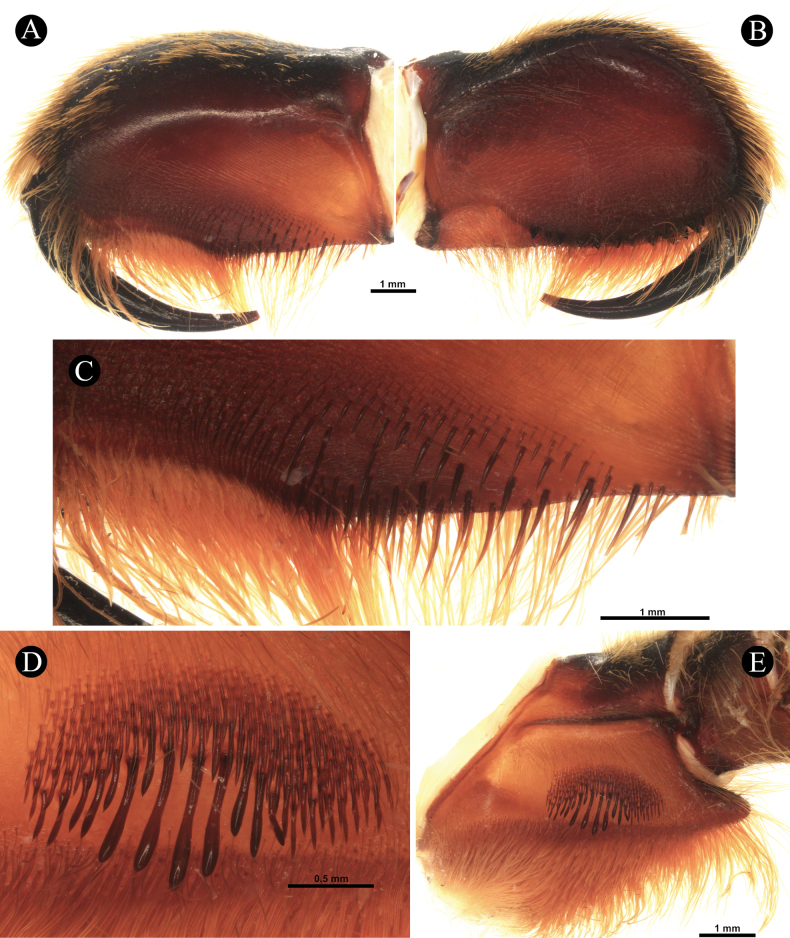
*Chilocosmiadichromata* comb. rest. ♂, SMNS Aran-004182 **A** left chelicera, retrolateral view **B** prolateral view **C** cheliceral strikers, retrolateral view **D** maxillary lyra on left prolateral maxilla **E** left maxilla, prolateral view.

**Figure 4.**
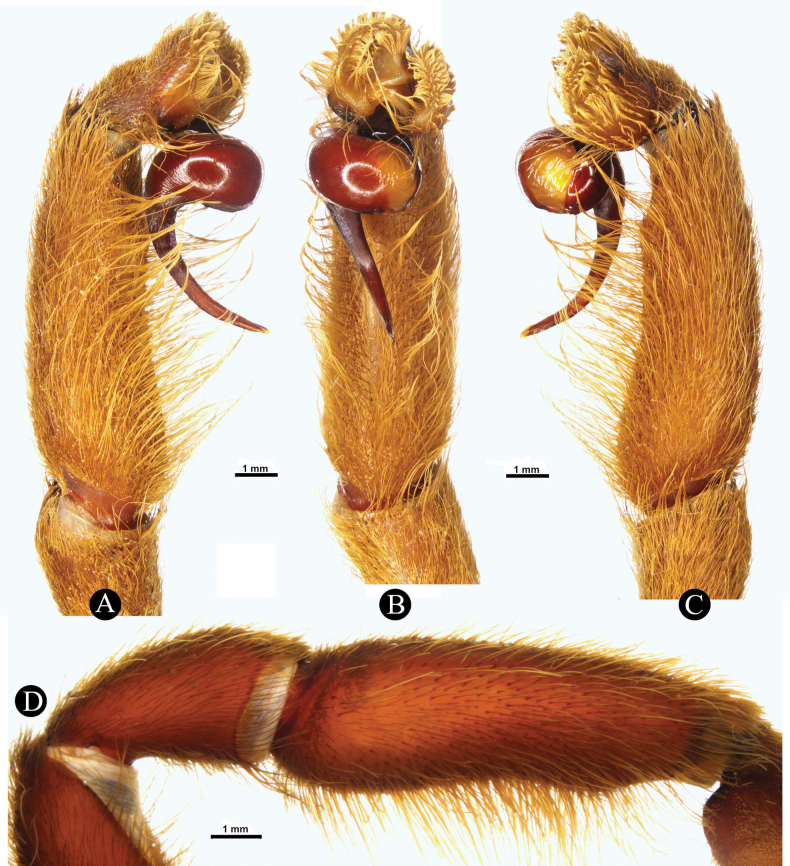
*Chilocosmiadichromata* comb. rest. ♂, SMNS Aran-004182 **A** left pedipalp, prolateral view **B** ventral view **C** retrolateral view **D** left palpal patella and tibia, prolateral view.

**Figure 5.**
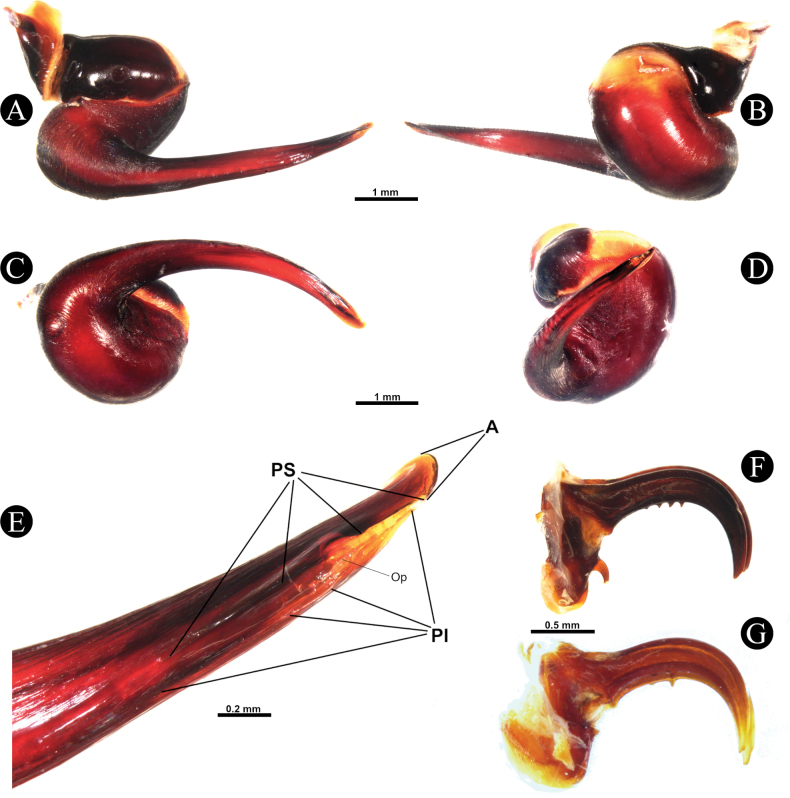
*Chilocosmiadichromata* comb. rest. ♂, SMNS Aran-004182 **A** left palpal organ, prolateral view **B** retrolateral view **C** ventral view **D** apical view **E** tip of embolus, prolateral view **F**, **G** claws on left tarsus IV of two ♂♂ of *C.dichromata***F**SMNS Aran-004182 **G**SMNS Aran-004183, retrolateral view. Abbreviations: PS- prolateral superior keel, PI- prolateral inferior keel, A- apical keel, Op- embolic opening.

**Figure 6.**
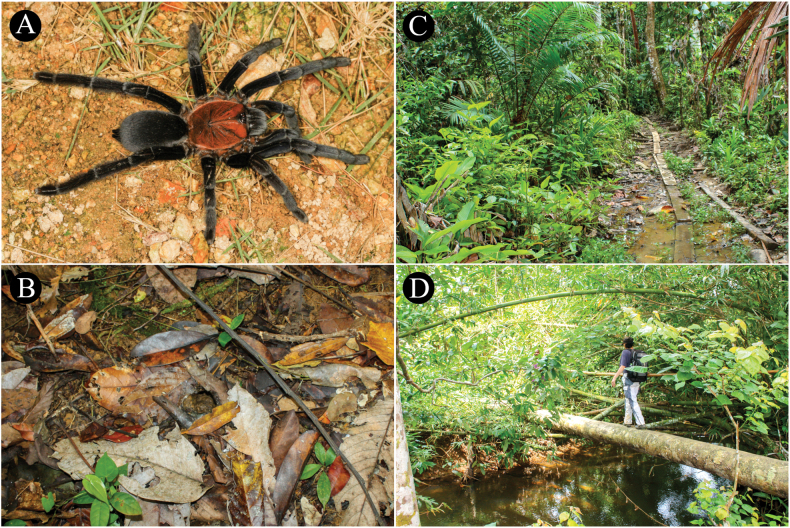
*Chilocosmiadichromata* comb. rest. natural habitat **A***C.dichromata* comb. rest., female, in situ **B** burrow entrance camouflaged with leaf litter **C**, **D** primary forest habitat in Sorong, West New Guinea, Indonesia. Photo credits: Frank Schneider.

Figs 2 , 3 , 4 , 5 , 6 , 20C, F , 21I, J

###### Type material examined.

Indonesia— **West New Guinea** • holotype ♀, SMF 37099-84; Sorong; SMF.

###### Other material examined.

Indonesia— **West New Guinea** • 2 ♂♂ SMNS Aran-004182 and Aran-004183, 5 ♀♀, SMNS Aran-004184, Aran-004185 and Aran-004162–004164, 3 j, SMNS Aran-004186; Sorong; 00°47'36.4"S, 131°26'32.8"E, F. Schneider leg., 2014; SMNS.

###### Diagnosis.

See genus diagnosis.

###### Description.

Male (SMNS Aran-004182). Body length 38.50 (*n* = 2 38.50–38.62).

***Carapace*** (Fig. 2B). 17.2 long, 14.9 wide, anterior width 8,95, cephalic height 4.9, cephalic region 11.3 long, thoracic region 4.79 long. **CI** 86.62, **CLI** 65.69, **CHI** 28.48. Fovea width 1.85, curve length 2.11, procurved (Fig. 2E). Carapace has four pairs of furrows, mainly covered with yellowish brown setae directed towards fovea, lateral profile low and flat, and integument dark brown. Setation: **TS**(a), rows of very short pale yellowish brown setae covering entire carapace, directed towards fovea and OT anteriorly. Posterior surface of OT with few long pale brown TS. **TS**(b), long pale yellowish brown setae at carapace margin, anterior margin pale brown. **SC**(a), brown flat scales sparsely covering OT. **SC**(b), sparse, pale yellowish brown, acicular scales covering carapace, anteriorly.

***Eyes*** (Fig. 2D). Ocular Tubercle 3.12 long, 2.28 wide, OT integument entirely dark. AME round, rest of the eyes ovoid. Anterior row of eyes slightly procurved, posterior row of eyes recurved. Eyes: AME 0.69, ALE 0.79, PME 0.46, PLE 0.61. Interocular distances: AME-AME 0.38, ALE-ALE 1.94, PME-PME 1.48, PLE-PLE 2.21, AME-ALE 0.13, AME-PME 0.22, AME-PLE 0.56, ALE-PLE 0.23, ALE-PME 0.47, PME-PLE 0.14. **EI** (AME) 4.01, **EI** (ALE) 4.59.

***Chelicerae*** (Fig. 3A–C). length 9.72, dorsal width 3.27, lateral width 5.99, fang curve length 7.29. Teeth 12, mesoventral denticles sparse 45. Setation: **TS**, long pale brown setae on dorsal and upper 1/2 retrolateral surface, longer anteriorly. Lower 1/2 of retrolateral surface posteriorly with rows of pallid filiform setae and anteriorly with patch of pallid needleform setae. Mesoretrolateral surface with rows of very sparse setae basally spiniform and anteriorly needleform. Mesoprolateral surface with intercheliceral setae, arcuate strip of rows of pallid needleform setae originating basally. Lower 1/2 prolaterally sparsely covered with setae, brown needleform at lower rows, and pale brown filiform at upper rows. **SC**, flat, translucent, pale brown scales, covering dorsal and upper retrolateral surface. Integument mostly dark brown. Cheliceral strikers (Fig. 3C): 111, 0.20–1.03, dark, long spiniform with filiform ends.

***Maxillae*** (Fig. 3D, F). Prolateral maxilla 6.33 long, 4.02 wide laterally, 3.58 wide ventrally. Maxilla prolaterally planoconvex, anterior lobe well pronounced, integument orangey brown, dark dorsally, basoventral cuspules 212. Lyra (Fig. 3D): lyrate patch, 2.24 long, 1.38 high, total rods 190, 10 or 11 rows, surrounded by very fine setae, denser above and distally. Short rods form an arcuate strip of patch. Longest club-shaped rods in lowest row 13, 0.27–0.78. Setation: **TS**, pale brown setae, longer ventrally, stronger at distodorsal margin. Lyrate patch surrounded by fine setae. Above maxillary suture, rows of ~ 13 stiff dark TS. **SC**, brownish white flat scales covering dorsally. Retrolateral surface smooth, with rows of short semi-transparent bristles at the lower margin.

***Labium and sternum*** (Fig. 2C, F). Labium: 2.30 long, 3.12 wide. Integument orangey brown, dark at posterior margin, anterior 1/3 with cuspules 371. Setation: **TS**(a), long, pale brown with pale filiform ends, covering labium anteriorly and laterally except on cuspule cluster, longer and greater at anterior edge, all pointing anteriorly. **TS**(b), brown, short needleform below cuspule cluster. Sternum: 8.2 long, 6.93 wide integument yellowish brown. Posterior sternal corner acuminate, lateral corners weakly acuminate. Setation: **TS**(a), long pale brown spiniform setae, pale apically, on entire sternum, but very sparse. **TS**(b), short pale brown setae covering sternum entirely. **TS** (c) fine, pallid, and short spiniform, at sternal margin. **SC**, brownish white flat scale mat densely covering entire sternum. Labiosternal sigilla 1.07 long, 0.34 wide, 0.83 apart. Sternal sigilla 2 pairs, median sigilla 0.69 long, 0.35 wide, 3.91 apart and 0.58 away from sternal margin adjacent to coxa II, posterior sigilla 1.08 long, 0.42 wide, 1.59 apart and 1.77 away from sternal margin adjacent to coxa III (Fig. 2C).

***Abdomen and spinnerets***. Abdomen: 17.56 long, 10.09 wide. ovular elongated, integument pale brown. Setation: **TS**(a), long, pale brown with darker bases, needleform, on entire abdomen, shorter ventrally, pallid on book lungs. **TS**(b), pallid short paddle-like, on book lungs and sparse on epigynal plate. **SC**, overlapping pale brown translucent scales covering entire abdomen. Spinnerets: PMS 2.39 long, 0.84 wide, PLS, anterior 2.30 long, 0.71 wide, median 1.82 long, 0.99 wide, posterior 3.65 long, 1.39 wide. Setation: **TS**(a), long, pale brown with darker bases, needleform, on dorsal PMS and PLS. **TS**(b), dark and short, pale brown paddle-like, intermixed with spigots, on PMS and PLS ventrally. **SC**, brownish white flat scales covering PLS dorsally.

***Genitalia*** (Figs 4A–C, 5A–E). Palpal Organ: almost 3/4 of palp tibia length (**POI** 73.70). Tegulum 2.67 long, 2.14 wide, twisted, widest medially, subtegular projection very weakly pronounced (Fig. 5A–D). Embolus length 3.30, width 0.54 basally, tip 0.10 wide (**EMI** 123.56), base robust, tapering distally, curved retrolaterally and ending to a weakly tip. There is a prolateral inferior keel (PI) and prolateral superior keel (PS) in the apical third of the embolus (Fig. 5E). The apical keel (A) is located at the tip of the embolus and is semicircular in shape (Fig. 5E). Embolic opening (Op) located between PS and PI near the tip. Basal lobe is not present.

***Legs***. Leg formula: IV, I, II, III. **RF** ~ 91.77, **LLI** (I) 26.54, **LLI** (IV) 21.15, **DLI** (I) 23.18, **DLI** (IV) 18.49, **MI** (I) 76.25, **MI** (IV) 132, **TI** (I) 69.67, **TI** (IV) 45.45. Leg lengths (fem, pat, tib, met, tar/cym): Palp 26 (10, 4.2, 8.1, -, 3.7) Leg I 61.3 (16.5, 8.1, 16, 12.2, 8.5) Leg II 55.6 (15, 7.1, 13, 12.5, 8) Leg III 49.5 (13, 6, 9, 14.5, 7) Leg IV 66.8 (17, 6, 15, 19.8, 9). Leg lateral width (fem, pat, tib, met, tar/cym): Palp (3.24, 2.92, 3.21, -, 2.46) Leg I (4.15, 3.51, 3.62, 2.82, 2.17) Leg II (4.33, 3.36, 3.16, 2.37, 1.94) Leg III (4.88, 3.45, 3.04, 2.45, 1.91) Leg IV (3.99, 3.47, 3, 1.96, 1.71) Leg dorsal width (fem, pat, tib, met, tar/cym): Palp (3.19, 2.93, 3.21, -, 2.88) Leg I (3.38, 3.30, 2.84, 2.18, 2.51) Leg II (3.40, 3.13, 2.59, 2.05, 2.07) Leg III (4.22, 3.17, 2.42, 1.90, 2.13) Leg IV (3,40, 3,06, 2.54, 1.89, 1.46). Cymbium bipartite. Tarsi IV transversely cracked apically.

***Leg setation and spines***. Setation (femur to tarsus): **TS** on all legs pale brown and short, longer on all metatarsi dorsally, femur of rear legs ventrally and on all tibia ventrally, dense on tibiae I and II, ventrally. There are only pale brown TS. **PB** is also not present on the palps. There are only pale brown TS. **SC**, brownish. Other sensory setae: **ETB**, pair of thin inverted L-shaped cluster of short dark brown setae, basoretrolateral on Met. I and II, single cluster on Met III. Cymbium with single cluster dorsally that broadens basally. **TB**(a), long and short filiform TB intermix with ETB in two rows, longest dorsally. **TB**(b), rows of unordered clavate TB, varying in size, present in all tarsi, and intermix with tarsal ETB. **CHS**, tiny, pale brown translucent erect sensilla tapering apically, present on the palpal and all leg femora to tarsi and intermixes with tarsal and metatarsal scopulae. Spines (dorsal-dorsoprolateral-dorsoretrolateral-ventral): Met II (0-0-0-2). Met III (0-1-1-2). Met IV (0-0-1-4).

***Coxae and trochantera***. Coxae: Length (coxa I, II, III, IV), 8.68, 6.05, 6.76, 6.83. Width (coxa I, II, III, IV), 4.46, 3.68, 4.17, 4.59. Setation: **TS**, long brown setae, dorsally and ventrally; strong and short spiniform setae, prolaterally and retrolaterally on all coxae. Trochantera: Length (troch. palp I, II, III, IV), 3.20, 3.21, 2.92, 3.38, 4.03. Width (troch. Palp I, II, III, IV), 2.77, 4.15, 3.88, 3.55, 3.72.

***Scopulae and claws***. Scopulae: cymbium scopulated ventrally. Tar. I, entire, with very few longer setae. Tar. II, entire, with very few longer setae. Tar. III, entire, with very few longer setae. Tar. IV, in the basal 1/4 divided by three or four long setae. The tarsus is cracked in the apical 1/4. Met. I covered almost all ventral surfaces, entire, but with a few very sparse long setae. Met. II, almost all ventral surface covered, entire, but with a few very sparse long setae. Met. III, almost all ventral surface covered, entire, but with a few very sparse long setae. Met. IV, covering 90% of ventral surface, divided by two or three rows of strong long setae. Claws: longest tarsal IV claw 1.70, no inferior third claw (Fig. 5G) (but present on some specimens; Fig. 5F), but there are a few large teeth on the claws whose number can vary from one claw to another.

***Color in life***. The opisthosoma and legs, except for the coxa and trochanter, are black in color (Fig. 2A). Coxa and trochanter as well as the carapace and the chelicerae basal segment are bronze-colored (Fig. 2A, C). The underside of the cephalothorax is also black. The area around the eye tubercle, as well as two diverging hairless stripes on the carapace, which frame the head, are dark brown in color. On each chelicerae basal segment there is also an elongated dark brown stripe in the middle of the bronze coloration.

###### Variation.

The third inferior claw is present or almost absent on some specimens and varies in length if present, which is also observed in other species in this study.

###### Etymology.

Greek *dýo chrómata* (δύο χρώματα) means two colors, which refers to the bicolored orange and black coloration of this species (Schmidt and von Wirth 1992).

###### Natural history and distribution.

The spiders live in tubes in the primary forest whose entrances are well camouflaged and difficult to find (Fig. 6B). In the habitat of *C.dichromata* comb. rest., there is apparently only this species of tarantula (Fig. 6). All tubes were filled with water to a depth of ~ 10 cm. The temperature in the tubes was 24–26 °C. The water in the tubes was ~ 2 °C cooler. Probably the spiders flee into the water when disturbed. Known only from Sorong, West New Guinea (now Southwest Papua Province), Indonesia.

##### ﻿Tribe Yamiini

Taxon classificationAnimaliaAraneaeTheraphosidae

Kishida, 1920

6EA77DA3-E742-55E0-9A5C-835F449353D7

Phlogiellini West et al., 2012 (synonymised by Nunn et al. 2016).

###### Included genera.

*Orphnaecus* Simon, 1892, *Phlogiellus* Pocock, 1897, *Selenobrachys* Schmidt, 1999, stat. rev.

##### ﻿Genus Orphnaecus

Taxon classificationAnimaliaAraneaeTheraphosidae

Simon, 1892

4375CAEA-0762-5452-B547-A8E7F00A5FA0

###### Type species.

*Orphnaecuspellitus* Simon, 1892, by monotypy.

###### Included species.

*O.adamsoni*Salamanes et al., 2022, *O.kwebaburdeos* (Barrion-Dupo et al., 2015), *O.pellitus* Simon, 1892.

###### Diagnosis.

*Orphnaecus* differs from all selenocosmiine genera (including *Chilocosmia* stat. rev.), except *Phlogiellus* and *Selenobrachys* stat. rev., by having a long prolateral superior keel (PS) (= retrolateral keel (R) in West et al. 2012) from tip to base with pronounced basal lobe (BL) on the embolus of males. *Orphnaecus* differs from *Phlogiellus* and *Selenobrachys* stat. rev., (i) in having a reniform patch, proximally broader, of short bacilliform rods, whereby the bacillae in the lowest row are larger, club-shaped, and rounded at the tip, prolaterally (Fig. 20A, D, G). It can also be distinguished from *Selenobrachys* stat. rev. in having long and dense dorsal palpal brush of setae on patella in males (Fig. 15A), in having a palpal tibia in males proximally swollen and distally tapering (Fig. 15A), and in having long acicular femoral setation on prolateral femur I (Fig. 21A–D).

###### Remarks.

Raven (1985) and Sivayyapram et al. (2020) distinguished *Orphnaecus* from other selenocosmiine genera in lacking an unpaired third claw at least on leg IV. However, the absence of a third claw cannot stand alone as the only diagnostic and defining characteristic of *Orphnaecus* against its known species that possess a third claw, while their genital and lyrate morphology is the same. In the *Orphnaecus* specimens (including syntypes of *O.pellitus*) mentioned and examined herein, we were able to find adult specimens within one species and one locality with a developed third claw, as well as those with a very reduced 3^rd^ claw, and even a few specimens that did not have a third claw at all (Fig. 5G). Raven (1985) may have examined a type specimen from the same type series of *O.pellitus* with no third claw. Most of the syntypes and newly collected specimens of *O.pellitus* possess an unpaired third claw on leg IV (Fig. 15E). Congeners, herein directly examined (types and non-types), also possess a third claw on leg IV. Simon (1892) distinguished *Orphnaecus* in having tiny and more separated eyes caused by troglomorphic adaptation. However, this is only phenotypic plasticity and cannot serve as a defining characteristic of the genus, as it arises solely from prolonged habitation in completely dark cave environments.

###### Distribution.

Philippine endemic: Luzon Is. (Simon 1892), Polillo Is. (Barrion-Dupo et al. 2015), Catanduanes Is., Masbate Is. (West et al. 2012), Negros Is. (Lüddecke et al. 2018), Dinagat Is. (Salamanes et al. 2022), and Mindanao Is. (Fig. 22). Probably widespread from Luzon PAIC, West Visayas PAIC, to Mindanao PAIC.

###### Etymology.

Orphnaeus, one of the four horses that drew the golden chariot of Hades, the king of the underworld in Greek mythology, attached with the suffix -*cus* (probably to avoid homonymy with a centipede genus, *Orphnaeus* Meinert, 1870). The type species *O. pellitus* is possibly a troglobitic species, exhibiting troglomorphism and spending its whole life inside the cave (Simon 1892). Gender is masculine.

##### ﻿Genus Selenobrachys

Taxon classificationAnimaliaAraneaeTheraphosidae

Schmidt, 1999 stat. rev.

410F2332-9685-5190-92E7-CBDAB2140A79

###### Type species.

*Selenobrachysphilippinus* Schmidt, 1999, comb. rest., by original designation and monotypy.

###### Included species.

(2 species) *S.philippinus* Schmidt, 1999, comb. rest., *S.ustromsupasius* sp. nov.

###### Diagnosis.

**Figure 7.**
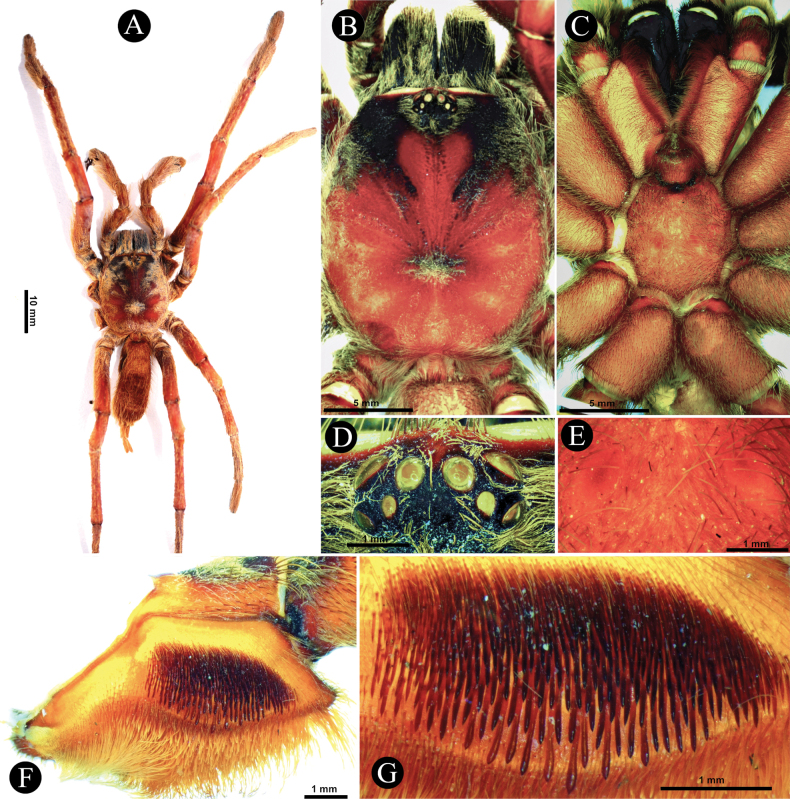
*Selenobrachysphilippinus* comb. rest. ♂, PASI ara0006 **A** dorsal habitus **B** prosoma dorsal view **C** ventral view **D** ocular tubercle, dorsal view **E** posterior sternal sigilla **F** left maxilla, prolateral view **G** lyra on left prolateral maxilla.

**Figure 8.**
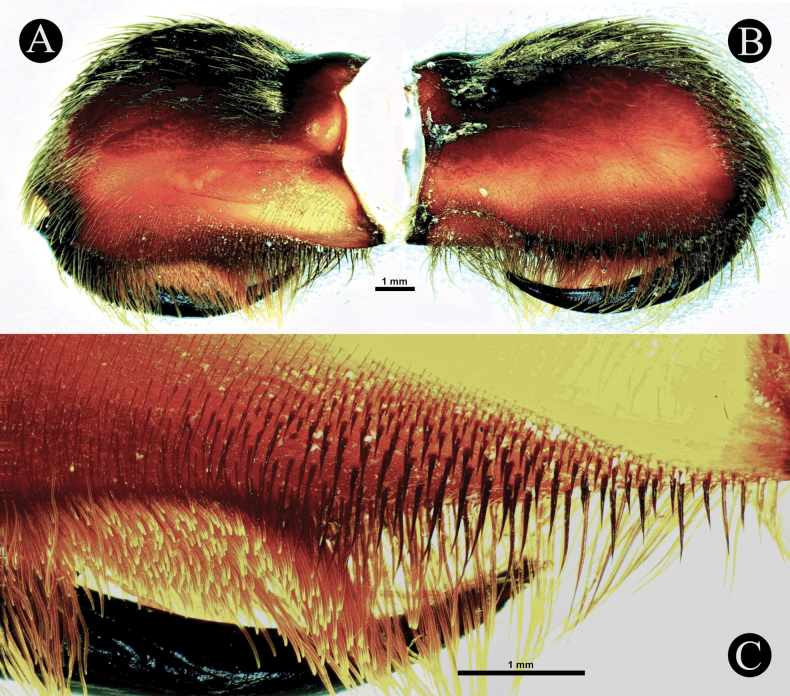
*Selenobrachysphilippinus* comb. rest. ♂, PASI ara0006 **A** left chelicera, retrolateral view **B** prolateral view **C** cheliceral strikers, retrolateral view.

**Figure 9.**
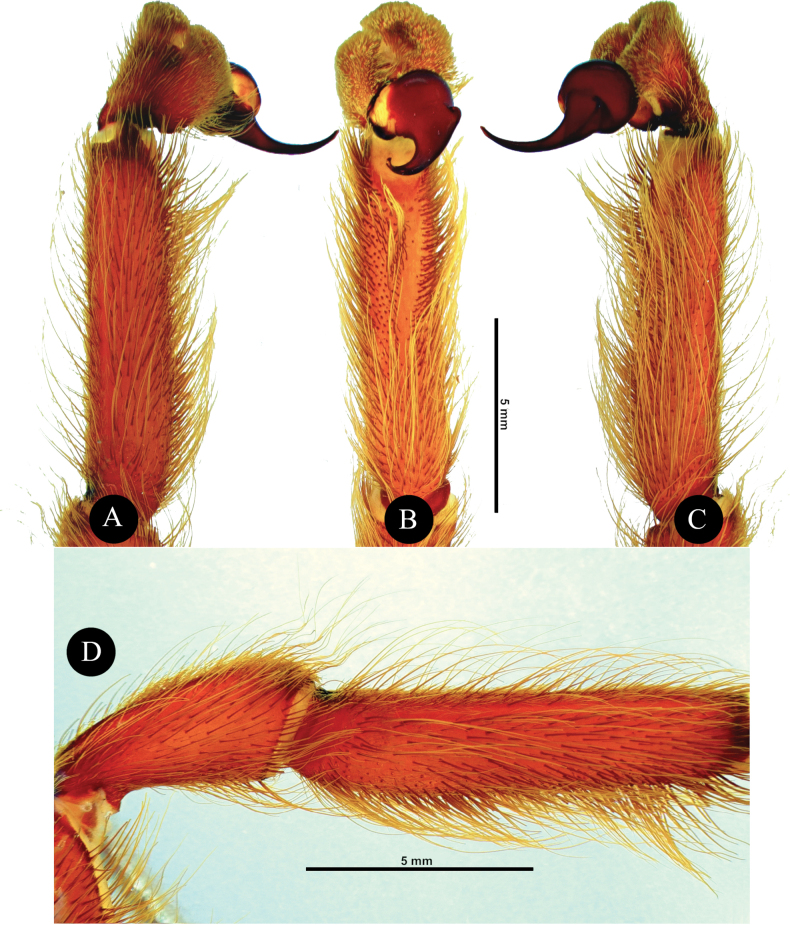
*Selenobrachysphilippinus* comb. rest. ♂ PASI ara0006, left pedipalp **A** tibia, cymbium, and palpal organ, prolateral view **B** ventral view **C** retrolateral view **D** left patella to tibia, retrolateral view.

**Figure 10.**
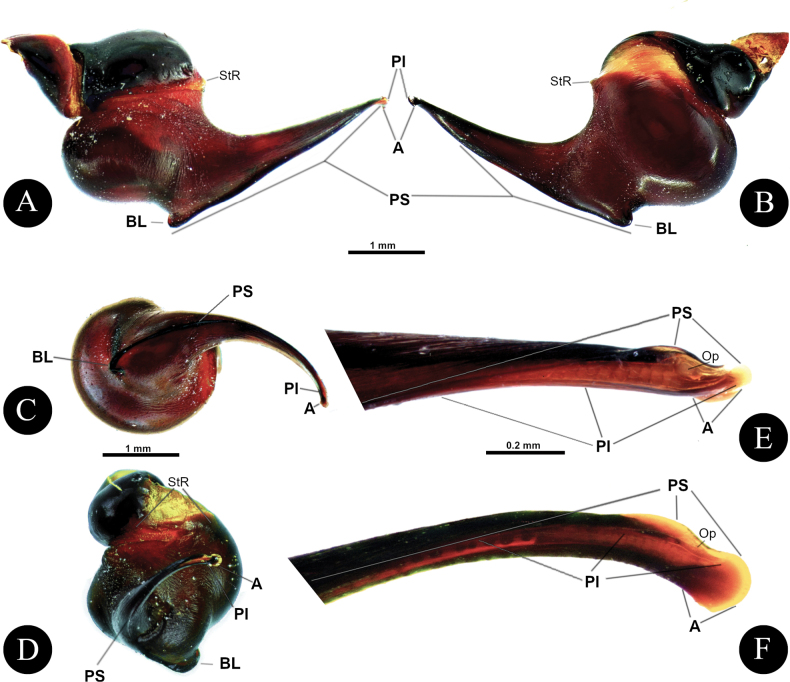
*Selenobrachysphilippinus* comb. rest. ♂, PASI ara0006, left palpal organ **A** prolateral view **B** retrolateral view **C** ventral view **D** apical view **E** tip of embolus, dorsal view **F** prolateral view. Abbreviations: PS- prolateral superior keel, PI- prolateral inferior keel, A- apical keel, BL- basal lobe, Op- embolic opening, StR- subtegular ridge.

*Selenobrachys* stat. rev. differs from all other selenocosmiine genera (including *Chilocosmia* stat. rev.), except sister genera *Orphnaecus* and *Phlogiellus*, in having a long prolateral superior keel (PS) (= retrolateral keel in West et al. 2012) from base to tip with a pronounced basal lobe (BL) on the embolus of males (Figs 10A–F, 14A–E). *Selenobrachys* stat. rev. differs from *Orphnaecus*, and *Phlogiellus* (i) in having an ovoid proximally truncated and distally mildly tapering lyrate patch on the prolateral maxilla, with rows of strong paddle-shaped bacillae possessing thick and strong shafts (Figs 7F, G, 1E, F, 17E, F, 20E) (reniform lyrate morphology for *Orphnaecus*; see above and Fig. 20G; rudimentary patch of needleform rods, if present, for *Phlogiellus*; see Nunn et al. 2016) and where the largest ones in the lowest row have a more pointed tip in prolateral view (Fig. 20E); (ii) in having greater number of cheliceral strikers (< 150; excluding tertiary rows) (Figs 8C, 13C, D, 17C, D); (iii) in having a long and cylindrical palpal tibia in males (Figs 9A–D, 15B, C) (proximally swollen and distally tapering in *Phlogiellus* and *Orphnaecus*; see Fig. 15A and Nunn et al. 2016); and (iv) in having a broad and short, not reduced ends, almost symmetrical, tombstone-shaped spermathecal lobe in females (Fig. 18A, B). It further differs from *Orphnaecus* in lacking long and dense dorsal scopulate palpal brush in adult males (Figs 9D, 15B, C) and in having short sword-like femoral setation on prolateral femur I (Fig. 21E–H). It also differs from *Phlogiellus* in having a greater number of labial cuspules (~ 331–760) and a wider fovea than the ocular tubercle (Nunn et al. 2016; Sivayyapram et al. 2020).

###### Distribution.

Philippine endemic: Negros Is. (Schmidt 1999; West et al. 2012) and Romblon Is. (this study; Fig. 22). Probably restricted to West Visayas PAIC and Romblon PAIC.

###### Etymology.

A combination of two generic names, *Selenocosmia* and *Chilobrachys* (*Seleno- + -brachys*) (Schmidt 1999). Gender is masculine.

##### ﻿ Selenobrachys philippinus

Taxon classificationAnimaliaAraneaeTheraphosidae

Schmidt, 1999, comb. rest.

3CC1EB30-ACC8-53A8-A024-8C6C6B4867D0

Figs 7 , 8 , 9 , 10 , 18B , 20B, E , 21G, H

###### Type material examined.

Philippines: Negros Island— **Negros Occidental Prov.** • holotype ♀, SMF 39202-84; Mambucal (now Mambucal Resort and Wildlife Sanctuary); SMF.

###### Other material examined.

Philippines: Negros Island— **Negros Occidental Prov.**• ♂, PASI ara0006, 9 ♀♀, 8 j, UST-ARC 0112–UST-ARC 0127 (field#NOM1A-01–NOM1A-16); Mambucal Resort and Wildlife Sanctuary; 365 m a.s.l., 26 Jun 2023, burrows under metamorphic rock boulders and crevices, LA Guevarra, DC Acuña, CN Noriega, R Enguito, LJS Villaflor leg.; PASI/ UST- ARC • ♀, SMNS Aran-004192, 5 ♂♂, SMNS Aran-004187–Aran-004191; Mount Canlaon; 2010, JM Verdez leg.; SMNS.

###### Diagnosis.

*Selenobrachysphilippinus* comb. rest. can be distinguished from its congener, *S.ustromsupasius* sp. nov., (i) in having longer leg IV than leg I (**RF**~ 89–98); (ii) in having broader posterior sigilla on the sternum (Fig. 7E); (iii) in having a palpal organ in males with lower palpal organ index (**POI** < 42), with embolus with narrower basal lobe (BL) (Fig. 10B), with thinner prolateral superior keel (PS) (Fig. 10A, B) but broader at the tip (Fig. 10E, F), and with less pronounced subtegular ridge (StR) (Fig. 10A, B); and (iv) in having lesser number of maxillary (< 305) and labial (< 471) cuspules. It also differs in color, having an orange to orangey brown general body coloration (Fig. 7A; Schmidt 1999).

###### Description.

Male (PASI ara0006). Body length 42.07 (*n* = 6: 41.43–43.98).

***Carapace*** (Fig. 7B). 17.51 long, 14.94 wide, anterior width 10.24, cephalic height 3.8, cephalic region 11.6 long, thoracic region 5.91 long. **CI** 85.32, **CLI** 66.25, **CHI** 21.70. Fovea width 2.27, curve length 2.43, procurved. Carapace has four pairs of weak furrows, lateral profile low and flat, and integument orangey brown, darker anteriorly. Setation: **TS**(a), rows of very short pale yellowish brown setae covering entire carapace, directed towards fovea and anteriorly to OT. Posterior surface of OT with pale brown short TS(a). **TS**(b), long pale yellowish brown setae at carapace margin, anterior margin light brown. **SC**(a), very short light brown flat scales sparsely covering OT. **SC**(b), pale yellowish brown acicular scales covering carapace, dense anteriorly, very sparse medially.

***Eyes*** (Fig. 7D). Ocular tubercle 2.09 long, 2.73 wide, integument dark, paler anteriorly. AME round, rest of the eyes ovoid. Anterior row of eyes slightly procurved, posterior row of eyes recurved. Eyes: AME 0.61, ALE 0.76, PME 0.51, PLE 0.51. Interocular distances: AME- AME 0.26, ALE-ALE 1.55, PME-PME 1.25, PLE-PLE 1.98, AME-ALE 0.18, AME-PME 0.13, AME-PLE 0.64, ALE-PLE 0.31, ALE-PME 0.35, PME-PLE 0.14. **EI** (AME) 3.48, **EI** (ALE) 4.34.

***Chelicerae*** (Fig. 8A–C). length 8.92, dorsal width 3.68, lateral width 6.28, fang curve length 7.48. Teeth 11, mesoventral denticles sparse ~ 35. Setation: **TS**, long pale brown setae on dorsal and upper 1/2 retrolateral surface, longer anteriorly. Lower 1/2 of retrolateral surface posteriorly with a patch of pallid filiform setae, anteriorly needleform. Mesoretrolateral surface with rows of very sparse setae basally spiniform, anteriorly needleform. Mesoprolateral surface with intercheliceral setae in arcuate strip of rows of pallid needleform setae originating basally. Lower 1/2, prolaterally, sparsely covered with brown needleform setae, filiform above. **SC**, flat, translucent, brownish white scales, covering dorsal and upper retrolateral surface. Cheliceral strikers (Fig. 8C): Primary rows, ~ 16, 0.65–0.91 dark, long spiniform with filiform ends. Secondary rows, ~ 136, 0.21–0.55, dark long and short spiniform. Tertiary rows, ~ 114, 0.16–0.23, pallid very short needleform setae. Pseudostrikers long and pallid, and present ventrally.

***Maxillae*** (Fig. 7F, G). Prolateral maxilla 7.47 long, 4.53 wide laterally, 3.75 wide ventrally. Maxilla prolaterally planoconvex, anterior lobe well pronounced, integument orangey brown, darker dorsally, basoventral cuspules ~ 305. Maxillary lyra (Fig. 7G): lyrate patch dense, in ovoid shape, truncated proximally, mildly tapering distally, 3.90 long, 2.10 high, total rods ~ 539, on 10–11 rows, surrounded by very fine setae, denser above and distally. Short bacilliform rods ~ 496, 0.24–0.39, needleform. Longer rods ~ 43, 0.49–0.85, paddle-shaped with pointed ends, which ~ 12 have well-defined paddle blades, and thick strong shafts slightly curved outward, located at the lowest rows. Setation: **TS**, pale brown spiniform setae, longer ventrally, stronger at distodorsal margin. Lyrate patch surrounded by fine setae. Above maxillary suture, two rows of ~ 15 stiff dark spiniform TS. Retrolateral surface is smooth, with rows of short semi-transparent bristles at lower margin. **SC**, flat whitish yellow-brown scales covering dorsal surface.

***Labium and sternum*** (Fig. 7C, E). Labium: 2.32 long, 3.15 wide. Integument orangey brown, darker posteriorly, anterior 1/3 with cuspules ~ 471. Setation: **TS**(a), long, pale brown with pale filiform ends, covering labium anteriorly and laterally except on cuspule cluster, longer and greater at anterior edge, all pointing anteriorly. **TS**(b), dark, short needleform below cuspule cluster. Sternum: 8.20 long, 6.93 wide, integument yellowish brown. Posterior sternal corner acuminate and lateral corners weakly acuminate. Setation: **TS**(a), long pale brown spiniform setae, pale apically, on entire sternum but sparse medially. **TS**(b), fine, pallid, and short spiniform, at sternal margin. **SC**, white flat scale mat covering entire sternum. Labiosternal sigilla 1.49 long, 0.38 wide, 0.75 apart. Sternal sigilla 3 pairs, anterior sigilla 0.34 long, 0.22 wide, 4.53 apart, and 0.26 away from sternal margin adjacent to coxa I, median sigilla 0.81 long, 0. 34 wide, 3.93 apart, and 0.50 away from sternal margin adjacent to coxa II, posterior sigilla broad (Fig. 7E), 1.40 long, 0.58 wide, 1.60 apart, and 1.17 away from sternal margin adjacent to coxa III.

***Abdomen and spinnerets***. Abdomen: 18.69 long, 7.65 wide. ovular elongated, integument pale citron brown. Setation: **TS**(a), long, citron brown with darker bases, needleform, on entire abdomen, shorter ventrally, and pallid on book lungs. **TS**(b), rows of pallid and short paddle-like on book lungs and sparse on epigynal plate. **SC**, dense, overlapping flat, translucent, pale brown scales lightened by pale citron integument producing an orangey brown mat covering entire abdomen. Spinnerets: PMS 2.24 long, 0.74 wide, PLS, anterior 2.56 long, 1.52 wide, median 2.84 long, 1.12 wide, posterior 3.76 long, 1.00 wide. Setation: **TS** (a), long, citron brown with darker bases, needle form, on dorsal PMS and PLS. **TS**(b), dark and short, pale brown paddle-like TS, intermixed with spigots, on PMS and PLS ventrally. **SC**, flat brownish white scales covering PLS dorsally.

***Genitalia*** (Figs 9A–C, 10A–E). Palpal Organ: approximately 2/5 of palp tibia length (**POI** 41.45). Tegulum 1.92 long, 1.81 wide, globular, widest medially, subtegular ridge (StR) weakly pronounced (Fig. 10A, B). Embolus length 2.42, width 1.20 basally and 0.37 medially, tip 0.16 wide, Embolus length 1.26 times longer than tegulum length (**EMI** 126.04), base robust, tapering distally, curved retrolaterally and ending to a broad tip. Embolus has a long and stout prolateral superior keel (PS) (Fig. 10A, B) but broad at tip (Fig. 10E, F); has prolateral inferior keel (PI) short, emerged from the tip to rear at apical 1/5, below PS and embolic opening (Op) (Fig. 10E, F); apical keel (A) very short, emerged almost at the tip; embolic opening (Op) located between PS and PI near the tip (Fig. 10E, F). Basal lobe pronounced but not broad, projected proximally (Fig. 10A, B).

***Legs***. Leg formula: IV, I, II, III. **RF**~ 91.94, **LLI** (I) 20.93, **LLI** (IV) 16.39, **DLI** (I) 19.97, **DLI** (IV) 16.23, **MI** (I) 80.99, **MI** (IV) 124.10, **TI** (I) 59.78, **TI** (IV) 44.41. Leg lengths (fem, pat, tib, met, tar/cym): Palp 31.96 (11.08, 6.46, 10.47, -, 3.95) Leg I 66.41(18.11, 9.92, 16.73, 13.55, 8. 1) Leg II 58.56 (15.7, 8.16, 14.59, 12.86, 7.25) Leg III 51.81(13.04, 6.59, 11.54, 13.63, 7.01) Leg IV 72.23 (17.64, 7.68, 16.8, 20.85, 9.26). Leg lateral width (fem, pat, tib, met, tar/cym): Palp (2.64, 2.34, 2.56, -, 2.35) Leg I (4.05, 3.61, 3.06, 1.9, 1.28) Leg II (3.49, 3.35, 2.58, 1.45, 1.37) Leg III (3.61, 2.8, 2.39, 1.75, 1.2) Leg IV (3.77, 3.08, 2.34, 1.55, 1.1) Leg dorsal width (fem, pat, tib, met, tar/cym): Palp (2.63, 2.27, 2-,, 2.38) Leg I (3.75, 3.08, 2.96, 1.84, 1.63) Leg II (3.31, 2.69, 2.54, 1.65, 1.35) Leg III (3.43, 2.61, 2.44, 1.72, 1.31) Leg IV (3.25, 2.76, 2.48, 1.96, 1.27). Cymbium bipartite. Tarsi I–IV transversely cracked, shows transverse weakening or pallid region, tar. I and tar. II more anteriorly, tar. III and tar. IV medially.

***Leg setation and spines***. Setation (femur to tarsus): **TS**(a), brown spiniform setae on all legs, longer TS pale brown, longer on all femora, ventrally and on palp patella and tibia, thicker on leg III and IV, dense citron brown on tibiae I and II, ventrally. **TS**(b), short and pallid paddle-like setae, dense on ventral femur I, sparse on ventral palpal femur, ventral patella I, and ventral femur II. **FS**, prolateral femur I with dense field (less dense than females) of elongated sword-like TS. **PB**, thin layer of short, flat, pale brown, scales, present on dorsal palpal patella and dorsal palpal tibia but very sparse (Fig. 9D). **SC**, flat whitish scales, darkened by reddish brown integument, covering all legs. Other sensory setae: **ETB**, pair of thin inverted L-shaped clusters of short pale brown setae, starting from basolateral to dorsal Met. I and II, single cluster on Met III and IV. Cymbium with a single cluster dorsally that broadens basally. **TB**(a), long and short filiform TB intermix with ETB in two rows, longest dorsally. **TB**(b), rows of unordered clavate TB, varying in size, present in all tarsi, and intermix with tarsal ETB. **CHS**, tiny, pale brown translucent erect sensilla tapering apically, present on the palpal and all leg femora to tarsi and intermixes with tarsal and metatarsal scopulae. Spines (dorsal-dorsoprolateral-dorsoretrolateral-ventral): Met I (0-0-0-1), Met II (0-0-0-3). Met III (0-1-1-4). Met IV (0-1-1-3).

***Coxae and trochantera***. Coxae: Length (coxa I, II, III, IV), 8.16, 7.88, 6.78, 7.31. Width (coxa I, II, III, IV), 4.49, 3.97, 4.27, 4.21. Setation: **TS**(a), long pale brown setae, covering dorsal and ventral surfaces; **TS**(b), strong and short spiniform setae, prolaterally on all coxae; **TS**(c), patches of fine setal fringe present laterally on coxae, intermixed with TS; **TS**(d), Coxae I–IV have rows of short semi-translucent bristles, prolaterally, denser on coxae I and II. **SC**(a), flat, grayish brown scales, covering the ventral to retrolateral 1/2; **SC**(b), white, cottony, and acicular, covering the dorsal of all coxae. Trochantera: Length (troch. palp I, II, III, IV), 2.78, 3.95, 3.02, 1.84, 2.77. Width (troch. palp I, II, III, IV), 2.59, 3.99, 3.52, 3.44, 3.62.

***Scopulae and claws***. Scopulae: cymbium scopulated ventrally. Tar. I, entire, but intermixed with one or two longitudinal rows of very sparse short spiniform setae. Tar. II, entire, but intermixed with one or two longitudinal rows of very sparse short spiniform setae. Tar. III, entire, but intermixed with two or three longitudinal rows of short spiniform setae. Tar. IV, divided by four rows of strong and long spiniform setae. Met. I ventral surface almost completely scopulated, entire, but with one or two longitudinal rows of very sparse long setae. Met. II, almost all ventral surface covered, entire, but intermixed with one or two longitudinal rows of very sparse long setae. Met. III, covering 4/5 distally, entire, but intermixed with one or two longitudinal rows of very sparse long setae. Met. IV, covering 3/4 distally, divided by two or three rows of strong long setae. Claws: pair of claws present on all leg tarsi, with one to three teeth on each claw. Tarsal IV claw, 2.27, with unpaired inferior third claw. 0.20.

***Color in life***. Monochromatic. The reddish brown integument is lightened by whitish scales creating a uniform orange to orangey brown body coloration (Fig. 7A).

###### Variation.

The third inferior claw is absent or almost absent on some specimens and varies in length if present, which is also observed in other species in this study.

###### Natural history and distribution.

Spiders are found roaming outside near their burrows at night. Burrows, not self-dug, are found near streams under metamorphic rock boulders and crevices on mountain slopes. Known only from Mambucal Resort and Wildlife Sanctuary (at the foot of Mt. Canlaon) and Sipalay City (West et al. 2012) in Negros Occidental, Negros Island, Philippines. Reports from the internet in the islands of Panay, Guimaras, and Cebu are unverified and may represent a separate island endemic species of *Selenobrachys*.

###### Etymology.

The specific epithet is a masculine adjective derived from the country locality, the Philippines (Schmidt 1999).

##### ﻿ Selenobrachys ustromsupasius sp. nov.

Taxon classificationAnimaliaAraneaeTheraphosidae

61E4C0E1-35E5-5681-A12A-A8A582E5160C

https://zoobank.org/96F1B924-D25E-48A5-AEB9-4548078E2E15

Figs 11 , 12 , 13 , 14 , 15B–D , 16 , 17 , 18A , 21E, F

###### Type material examined.

Philippines: Romblon Island— **Romblon Prov. • *holotype*** ♂, UST-ARC 0002 (field#R01-02), ***paratypes*** 4 ♂♂, 8 ♀♀, 1 j, UST-ARC 0001, 0003–0014 (field#R01-01, R01-03–R01-14); Municipality of Romblon, Brgy. Tambac; 12°32.0106'N, 122°17.6595'E, 200 m a.s.l., Sep 2022, AB Mayor, L De Capiz, RM De Capiz leg. • ***paratypes*** 2 ♂♂, 4 ♀♀, 10 j, UST-ARC 0015–0030 (field#R02-01–R02-16); Municipality of Romblon, Brgy. Guimpingan; 12°35.2478'N, 122°17.2416'E, 80 m a.s.l., 24 Feb 2023, DC Acuña, LA Guevarra Jr., CN Noriega, MJ Fadri, GA Florendo Jr., CJ Cabisuelas leg.; UST-ARC.

###### Diagnosis.

**Figure 11.**
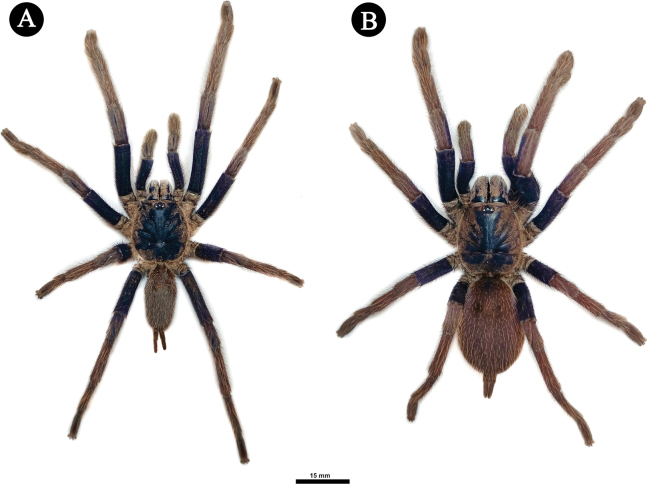
*Selenobrachysustromsupasius* sp. nov. dorsal habitus **A** holotype ♂, UST-ARC 0002 **B** paratype ♀, UST-ARC 0005.

**Figure 12.**
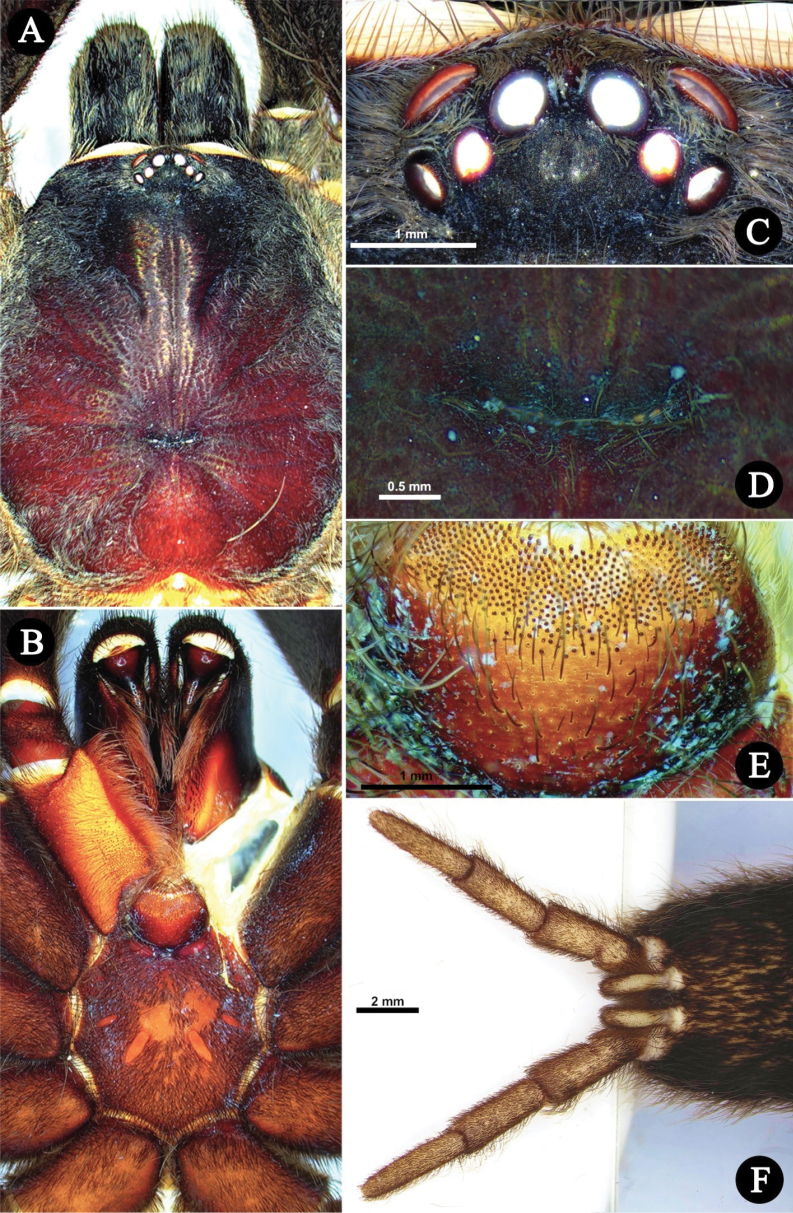
*Selenobrachysustromsupasius* sp. nov., holotype ♂, UST-ARC 0002 **A** prosoma, dorsal view **B** ventral view **C** ocular tubercle, dorsal view **D** fovea, dorsal view **E** labium, ventral view **F** spinnerets, ventral view.

*Selenobrachysustromsupasius* sp. nov. can be distinguished from its congener, *S.philippinus* comb. rest, (i) in having longer leg I than leg IV, with **RF** ~ 104–111; (ii) in having narrower posterior sigilla (Figs 12B, 16C); (iii) in having a palpal organ in males with greater palpal organ index (**POI** > 48), with embolus with broader basal lobe (Fig. 14B), with thicker prolateral superior keel (PS) (Fig. 14A, B) but stouter at tip (Fig. 14E), and with less pronounced subtegular ridge (StR) (Fig. 14A, B); and (iv) in having greater number of maxillary (> 365) and labial (> 608) cuspules. It also differs in color, having a brown to dark brown general body coloration (Figs 11A, B, 19G, H).

###### Description.

**Figure 13.**
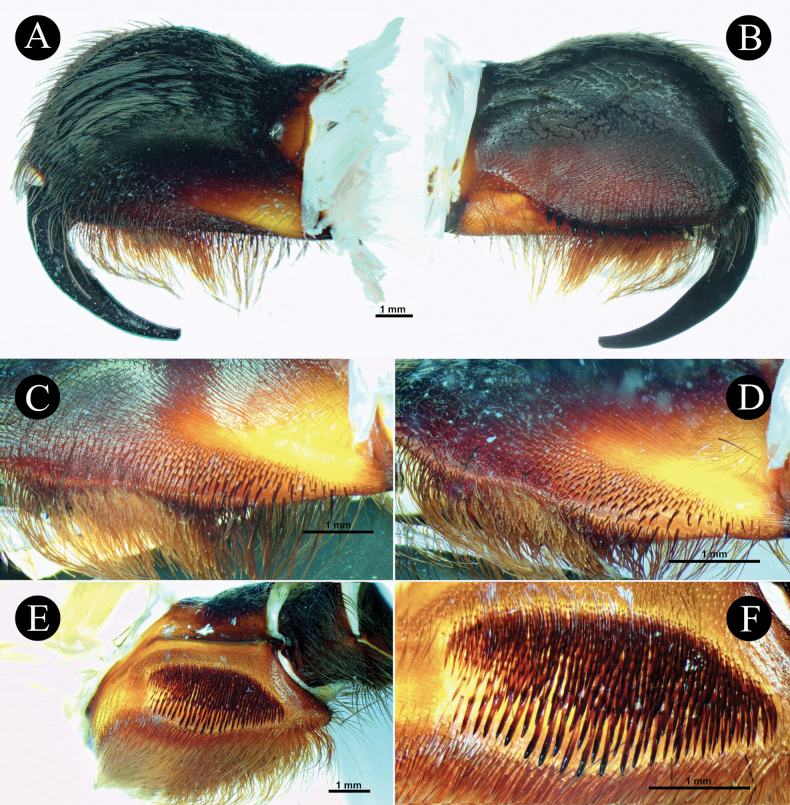
*Selenobrachysustromsupasius* sp. nov., holotype ♂, UST-ARC 0002, left chelicera and maxilla **A** left chelicera retrolateral view **B** prolateral view **C** cheliceral strikers retrolateral view **D** ventrolateral view **E** left maxilla, prolateral view **F** maxillary lyra on left prolateral maxilla.

**Figure 14.**
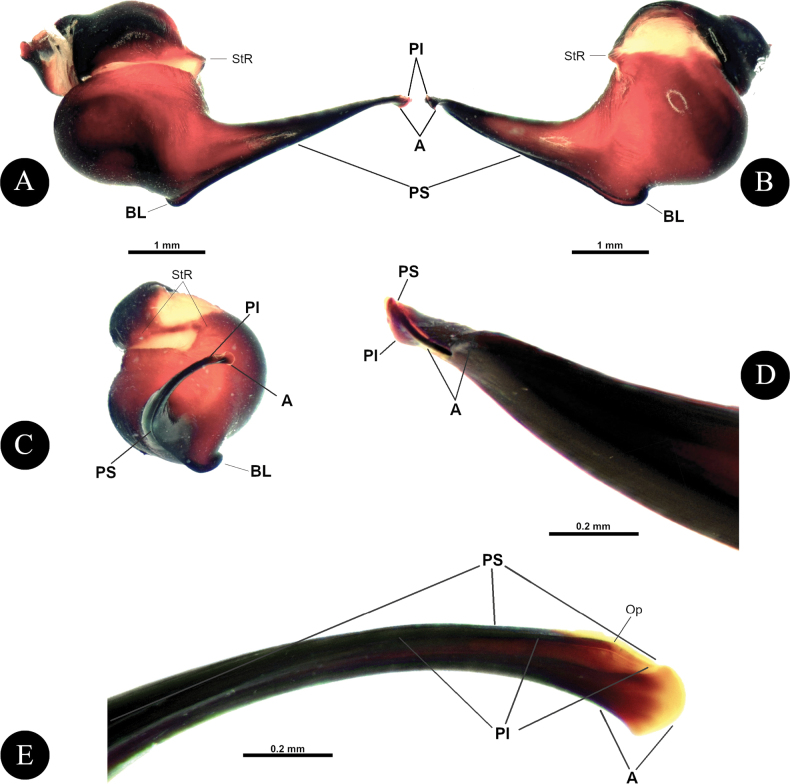
*Selenobrachysustromsupasius* sp. nov., holotype ♂, UST-ARC 0002, left palpal organ **A** prolateral view **B** retrolateral view **C** apical view **D** tip of embolus, retrolateral **E** prolateral view. Abbreviations: PS- prolateral superior keel, PI- prolateral inferior keel, A- apical keel, BL- basal lobe, Op- embolic opening, StR- subtegular ridge.

**Figure 15.**
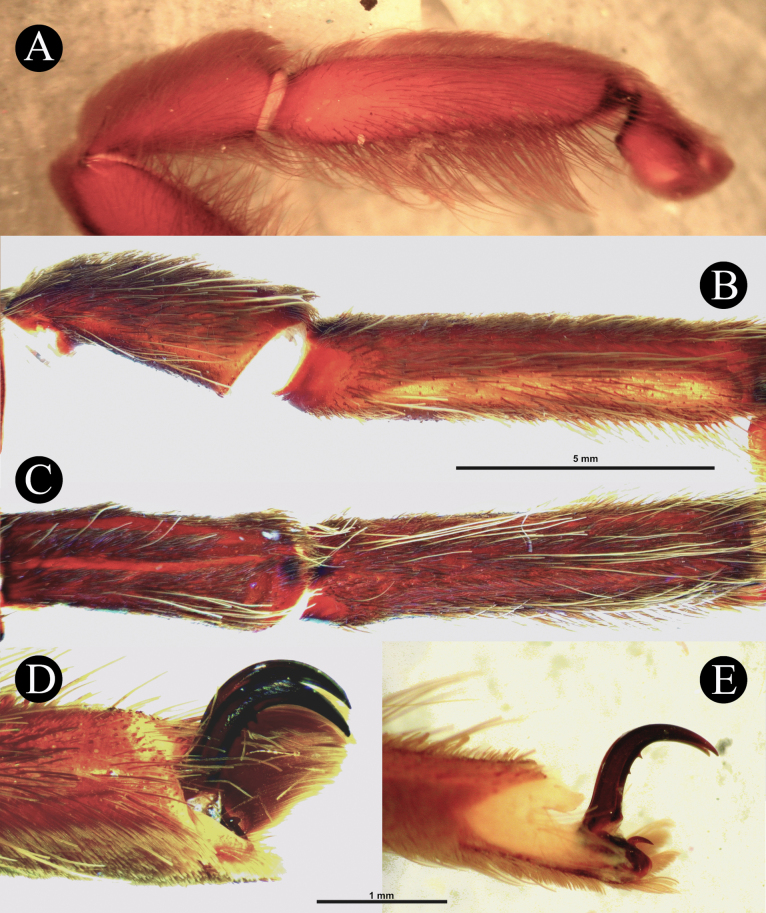
**B**–**D***Selenobrachysustromsupasius* sp. nov., holotype ♂, UST-ARC 0002, pedipalp and tarsus **B** Left palpal patella and tibia, prolateral view **C** dorsal view **D** claws on left tarsus IV, retrolateral view **A**, **E***Orphnaecuspellitus*, syntype ♂, MNHN AR4678 **A** left pedipalp, prolateral view **E** claws on left tarsus IV, retrolateral view.

**Holotype** ♂, UST-ARC 0002 (field#R01-02). Body length 43.18. (*n* = 7: 43.00–45.19) Figs 11A, 12–14, 15B–D.

***Carapace*** (Fig. 12A). Length, 17.41, median width 15.34, **CI** 88.11, **CLI** 65.88, **CHI** 22.75. Integument dark reddish brown. Caput profile low and flat, cephalic region slightly higher. Fovea procurved (Fig. 12D), width 1.72, curve length 1.89. Distance of fovea to posterior carapace margin 4.55; distance to OT 9.68. Setation: **TS**(a) long, thin, pale brown setae at carapace margin, pointing anteriorly. **TS**(b) long, strong, dark brown setae, pale brown apically, intermixed with TS(a) and SC, at clypeus, and few at the top of OT between AMEs. **TS**(c) rows of short pale or dark brown setae, pale brown apically, that run along radial grooves pointing to fovea. **SC**(a), very short, thick, flat, pale brown scales covering anterior 1/2 surface of OT. **SC**(b), mat of long, dense, whitish acicular scales, covering lateral sides and front of OT, pointing towards OT and carapace, sparse to none on middle surface, denser on coastal surface, mostly pointing anteriorly.

***Eyes*** (Fig. 12C). OT length 1.94, width 2.86. Clypeus 0.5, integument dark. Eyes anterior row slightly procurved, posterior row recurved. Eyes: AME 0.61 (round), ALE 0.78 (ovoid), PME 0.44 (ovoid), PLE 0.47 (ovoid). Interocular distances: AME-AME 0.31, PLE-PLE 1.97, ALE-ALE 1.59, PME-PME 1.24, ALE-PLE 0.21, AME-ALE 0.20, AME-PME 0.12, ALE-PME 0.32, PME-PLE 0.09, AME-PLE 0.55. **EI**(AME)3.50, **EI**(ALE) 4.48.

***Chelicerae*** (Fig. 13A–D). Dorsal length 8.51, dorsal width 3.79, lateral width 6.49. Fang curve length 6.68. Prolateral surface: more than upper 1/2 integument dark, lower surface reddish brown, with long brown fine setae, lowest portion above teeth darker and longer. Intercheliceral pegs absent but with rows of stiff setae. Retrolateral surface: integument upper 2/3 dark, lower surface reddish brown to amber, flat whitish scales (**SC**a), fine, white, cottony acicular scales (**SC**b), and long strong setae (**TS**) covering dorsal and upper retrolateral surfaces, greater anteriorly, patch of fine and long, mostly slightly curved setae at posterior area of lower retrolateral surface, patch of very fine setae with curved ends at anterior area of retrolateral surface, proximomedial setae needleform, very sparse. Long, pale reddish brown brush of setae ventrally. Teeth 11, mesoventral denticles sparse, ~ 41, on three or four rows. Lyrate region (Fig. 13C, D): 237–248 total strikers on six to seven horizontal rows, unordered, the strongest and longest strikers on lower rows. Primary rows ~ 11 (0.80–0.83 long) long dark brown with long curved filiform ends pointing distally. Secondary rows ~ 142 (0.17–0.66), dark spiniform with short curved filiform ends pointing inward. Tertiary rows ~ 90 (0.09–0.18), short, pallid, and needleform, located above secondary rows. Long, pallid pseudostrikers present ventrally.

***Maxillae*** (Fig. 13E, F). Prolaterally planoconvex, anterior lobe well pronounced, cuspules on inner basoventral surface ~ 365. Stridulatory organ (Fig. 13F): ~ 465 total bacilliform rods, in nine or ten rows, form long and dense ovoid patch, proximally truncated and distally mildly tapering (3.31 long, 1.70 high). Short bacilliform rods ~ 424, 0.22–0.53, needleform. Longer rods ~ 41, 0.55–0.77, paddle-shaped with thick and strong shafts and pointed ends, ~ 10 of which have well-defined paddle blades, located at lowest rows. Paddles flattened perpendicularly to maxillary surface. Setation: **TS**, brown setae, with upper part paler, present dorsally and ventrally, longest ventrally. Proximodorsal with erect fine pallid setae. Lyrate patch surrounded by fine setae. Above maxillary suture, two rows of ~ 20 stiff dark TS. **SC**, flat whitish scales covering dorsal surface. Retrolateral surface smooth, with rows of short semi-transparent bristles at lower margin.

***Labium and sternum*** (Fig. 12B, E). Labium (Fig. 12E): length 2.30, width 3.30, integument reddish brown, paler anteriorly. Labial cuspules ~ 608, dark, at apical 1/3. Setation: **TS**(a), long brown with filiform ends, covering labium anteriorly and laterally except on cuspule cluster, longer and denser at anterior edge, all pointing anteriorly. **TS**(b), dark, spiniform, on posterior surface. Sternum (Fig. 12B): sternum length 7.68, width 6.80, integument reddish brown, slightly darker at margin. Posterior sternal corner acuminate, lateral corners weakly acuminate. Labiosternal sigilla ovoid, 1.12 long and 0.40 wide, 0.72 apart. Sternal sigilla 3 pairs, ovoid. Anterior pair 0.28 long, 0.16 wide, 4.72 apart, and 0.16 away from the sternal margin adjacent to coxa I. Median pair 0.88 long, 0.24 wide, 3.88 apart, and 0.20 away from the sternal margin adjacent to coxa II. Posterior pair 1.44 long, 0.44 wide, 1.48 apart, and 1.16 away from the sternal margin adjacent to coxa III. Setation: **TS**(a), long and short dark spiniform setae on entire sternum, sparse on middle surface. **TS**(b), dark and pallid, short spiniform with filiform ends, at sternal margin. **SC**, flat grayish brown scales mat covering entire sternum.

***Abdomen and spinnerets***. Abdomen 14.70 long, 8.25 wide, ovular elongated, integument pale brown. Pedicel 1.8, pale brown, dorsally striated. Setation: **TS**, long brown setae, paler apically, covering entire abdomen, except book lungs and epigynal plate, denser laterally. Book lungs covered with short, pale brown spiniform setae. **PTS**, dark and short, covering book lungs and epigynal plate, intermixed with semi-transparent thin and short sensilla. **SC**, grayish brown, covering entire, sparse on book lungs and epigynal plate. All setae pointing distad. Spinnerets (Fig. 12F): PMS 2.02 long, 0.60 wide. PLS 9.52 long (ant. 3.12, mid. 2.84, pos. 3.56), and 2.88 wide (ant. 1.32, med. 0.92, post. 0.64). Setation: **TS**, long brown setae, paler apically, covering dorsally and laterally. **PTS**, dark and short, covering ventrally, intermixed with spigots. **SC**, grayish brown, sparse, present dorsally.

***Genitalia***. Palpal organ (Fig. 14A–E): almost 1/2 of palp tibia length (**POI** 48.35). Tegulum length 1.91, width 1.78. Embolus length 2.79, basal width 1.11, middle length 0.33, tip 0.21 wide. Tegulum globular, widest medially, subtegular ridge (StR) pronounced (Fig. 14A, B). Embolus 146 times longer than tegulum (**EMI** 146.07), robust at the base (Fig. 14A, B), tapering distally, Embolus has long and thick prolateral superior keel (PS) from base to tip (Fig. 14A, B) but stout at the tip (Fig. 14E); prolateral inferior keel (PI) short, emerged from the tip to rear at apical 1/5, located below PS and embolic opening (Op) (Fig. 14E); apical keel (A) very short, emerged almost at the tip (Fig. 14E); embolic opening (Op) located between PS and PI near the tip (Fig. 14E). Basal lobe pronounced and broad, projected proximally (Fig. 14B). Palpal tibia long and cylindrical (Fig. 15B, C).

***Legs***. Leg formula: I, IV, II, III. **RF** ~ 110.99, **LLI** (I) 20.90, **LLI** (IV) 18.83, **DLI** (I) 20.85, **DLI** (IV) 18.67, **TI**(IV) 44.03, **MI**(IV) 108.80. Leg lengths total (fem., pat., tib., met., tar. /cym.): Palp 31.51 (11.24, 6.82, 9.72, n/a, 3.73), Leg I 64.31 (17.72, 9.63, 16.01, 13.19, 7.76), Leg II 53.39 (14.59, 7.88, 12.36, 11.65, 6.91), Leg III 45.83 (12.24, 6.31, 9.63, 11.51, 6.14), Leg IV 57.94 (15.47, 6.89, 13.86, 15.08, 6.64). Leg lateral widths (fem., pat., tib., met., tar. /cym.): Palp (2.53, 2.38, 2.24, n/a, 2.61), Leg I (4.23, 3.21, 2.85, 1.76, 1.39), Leg II (3.85, 3.16, 2.40, 1.58, 1.32), Leg III (4.19, 2.94, 2.47, 1.56, 1.25), Leg IV (3.52, 2.89, 2.17, 1.43, 0.91). Leg dorsal widths (fem., pat., tib., met., tar. /cym.): Palp (2.74, 2.50, 2.07, n/a, 2.50), Leg I (3.56, 3.36, 2.99, 1.93, 1.57), Leg II (3.38, 3.04, 2.45, 1.78, 1.40), Leg III (3.66, 2.75, 2.54, 1.73, 1.29), Leg IV (3.20, 2.52, 2.33, 1.65, 1.12). Cymbium bipartite. Tarsi I–IV transversely cracked, shows transverse weakening or mild pallid region, tar. I and tar. II more anteriorly, tar. III and tar. IV medially.

***Leg setation* (*femora to tarsi*) *and spines***. Setation: **TS**, long, dark brown setae with contrasting pale brown filiform ends, present on palp and to all legs, but sparse to all tarsi, prolateral femora I and II, and retrolateral femur IV, all pointing distad, with some erect rows on leg1 dorsally. **PTS**, short and pallid, dense on ventral femur I, sparse on ventral palpal femur, prolateral to ventral patella I, and ventral femur II. **FS**, prolateral femur I with dense field (less dense than females) of elongated sword-like setae. **SC**, reflective grayish brown, covering all legs, longer and fine on all femora, pale brown on all patellae. **PB**, grayish, present on dorsal palpal patella but not long and dense, also present on dorsal palpal tibia but very sparse (Fig. 15B, C). **ETB**, short brown setae in pair of thin inverted L-shaped clusters, starting from basolateral to dorsal Met I to II, single cluster on Met III to IV. Cymbium with single cluster dorsally that broadens basally. **TB**, long and short filiform intermix with ETB in two rows, longest dorsally. Rows of clavate TB, unordered, varying in size, present in all tarsi and cymbium, and intermix with tarsal ETB. **CHS** tiny, pale brown translucent erect sensilla tapering apically, present on the palpal and all leg femora to tarsi and intermix with tarsal and metatarsal scopulae. Spines: (dorsal-dorsoprolateral-dorsoretrolateral-ventral): Met I (0-0-0-1), Met II (0-0-0-3). Met III (0-1-1-3). Met IV (0-1-1-5).

***Coxae and trochantera***. Coxae: Palp coxa (see *Maxillae*). Lengths (coxa I, II, III, IV) 7.52, 5.80, 5.20, and 5.60. Widths (coxa I, II, III, IV) 4.00, 3.48, 3.44, 3.88. Setation: **TS**, long brown setae, dorsally and ventrally; strong and short spiniform setae, prolaterally on all coxae. **SC**(a), flat scales covering the ventral to retrolateral 1/2. **SC**(b), white, cottony, acicular scales covering the dorsal of all coxae. Patches of fine setal fringe present laterally on coxae, intermixed with short spiniform setae. Coxae I–IV have rows of short semi-translucent bristles, prolaterally, denser on coxae I and II. Trochantera: Lengths (troch. palp, I, II, III, IV) 2.12, 3.52, 3.32, 3.08, 2.72. Widths (coxa palp, I, II, III, IV) 2.48, 3.40, 2.96, 3.12, 3.28.

***Scopulae and claws***. Scopulae: cymbium scopulated ventrally. Tar. I, entire, but intermixed with longitudinal one or two rows of very sparse short spiniform setae. Tar. II, entire, but intermixed with longitudinal one or two rows of very sparse short spiniform setae. Tar. III, entire, but intermixed with longitudinal two or three rows of strong, long setae. Tar. IV, divided by four rows of strong, long setae. All tarsi with a bald spot ventrobasally. Met. I, almost all ventral surface covered, entire, but intermixed with longitudinal one or two rows of very sparse long setae. Met. II, most ventral surface covered, entire, but intermixed with longitudinal one or two rows of very sparse long setae. Met. III, covering 4/5 distally, entire, but intermixed with longitudinal one or two rows of very sparse long setae. Met. IV, covering 3/4 distally, divided by two or three rows of strong long setae. Claws: pair of claws present on all leg tarsi with one to three teeth on each claw. Unpaired inferior third claw 0.13, very short, present on tarsus IV (Fig. 15D).

***Color in life***. Slightly dichromatic, dark on carapace and all femora, and covered with pale brown setae on leg patellae, tibiae, metatarsi, and tarsi, including trochantera, sternum, abdomen, and spinnerets (Fig. 11A). Almost uniformly dark brown with a mild purplish blue sheen reflected by scales just after ecdysis, which fades in a few days.

**Figure 16.**
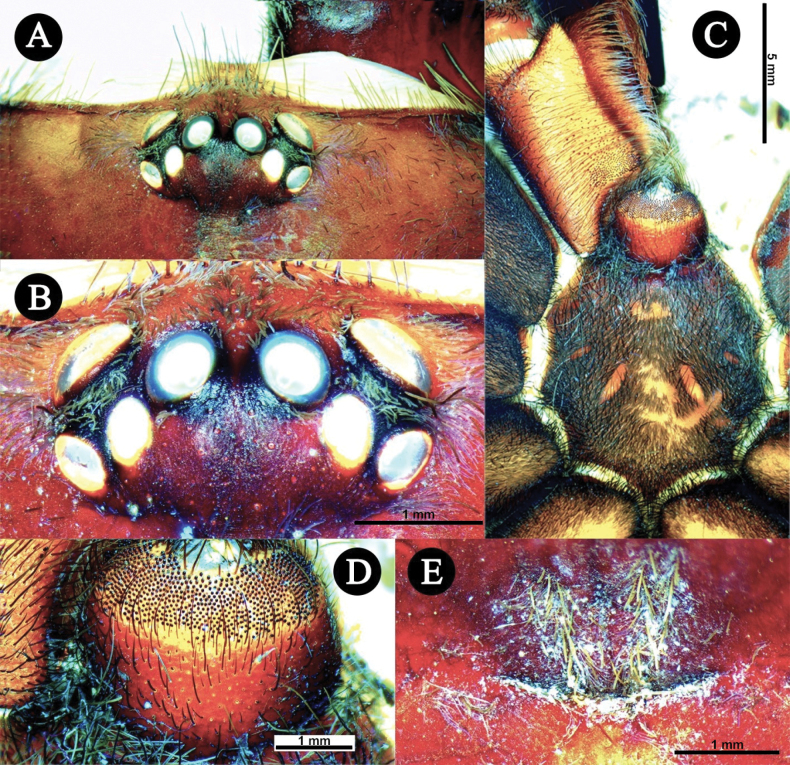
*Selenobrachysustromsupasius* sp. nov., paratype ♀, UST-ARC 0005 **A**, **B** ocular tubercle, dorsal view **C** prosoma, ventral view **D** labium, ventral view **E** fovea, dorsal view.

**Figure 17.**
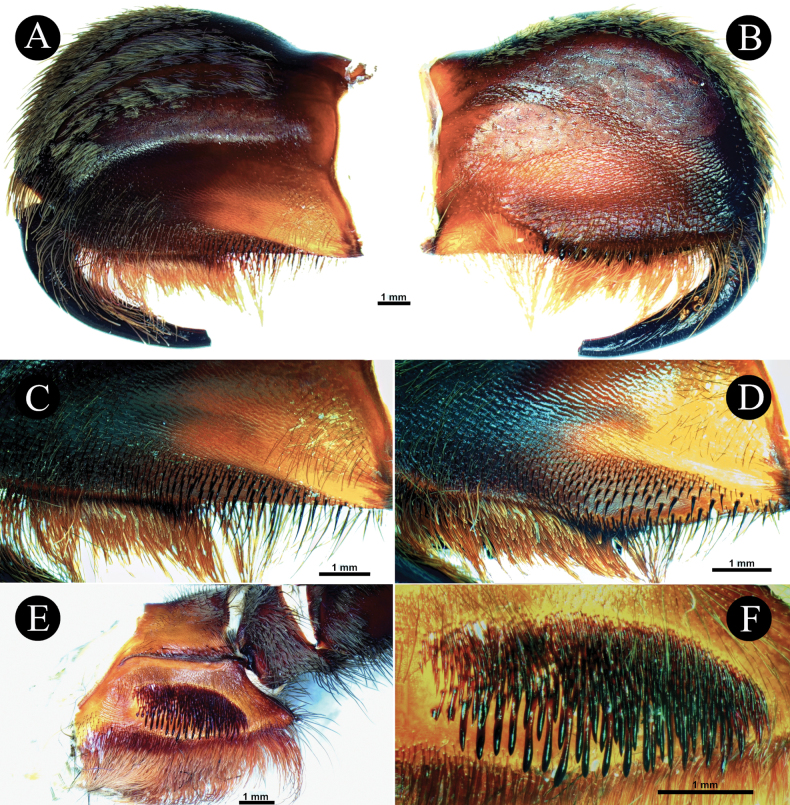
*Selenobrachysustromsupasius* sp. nov., paratype ♀, UST-ARC 0005, left chelicera and maxilla **A** left chelicera, retrolateral view **B** prolateral view **C** cheliceral strikers, retrolateral view **D** ventrolateral view **E** left maxilla, prolateral view **F** maxillary lyra on left maxilla.

**Figure 18.**
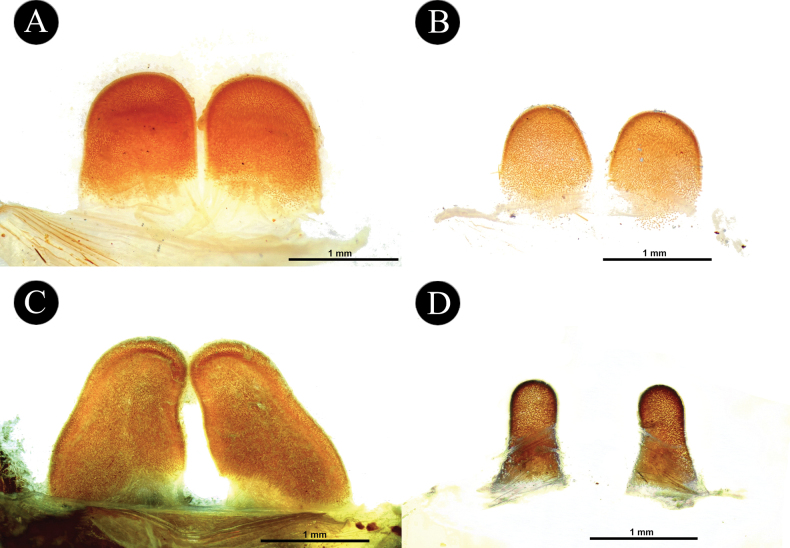
Spermathecae, dorsal view **A***Selenobrachysustromsupasius* sp. nov., paratype ♀, UST-ARC 0005 **B***Selenobrachysphilippinus* comb. rest., ♀, UST-ARC 0117 **C***Orphnaecuskwebaburdeos*, ♀, UST- ARC 0060 **D***Orphnaecuspellitus*, ♀, UST-ARC 0031.

**Paratype** ♀, UST-ARC 0005 (field#R01-05). Body length 53.85 (*n* = 12: 42.56–55.13) Figs 11B, 16, 17, 18A, 21E, F

***Carapace*** (Fig. 16A). Length 18.82, median width 15.61, anterior width 9.47, **CI** 82.94, **LI** 66.84, **CHI** 27.42. Integument reddish brown. Caput profile low and flat, and cephalic region slightly higher. Fovea procurved (Fig. 16E), width 2.13, with curve length 2.22. Distance of fovea to posterior carapace margin 4.74, distance to ocular tubercle 10.67. Setation: **TS**(a), long, thin, pale brown setae at the margin of carapace, directed anteriorly; **TS**(b), long, strong, dark brown setae and pale brown apically, intermixed with TS(a) and SC(b), at clypeus, and one or two at top of OT between AMEs; **TS**(c), rows of short pale brown or dark brown setae, pale brown apically, that runs to radial grooves pointing to fovea; **SC**(a), short, flat, pale brown scales covering anterior 1/2 surface of OT; **SC**(b), mat of satiny white acicular scales covering carapace but sparse to absent medially and greater on margin, most directed anteriorly (denser and longer on male).

***Eyes*** (Fig. 16A, B). Ocular tubercle (OT) length 2.16, width 2.99. Clypeus 1.2. Eyes anterior row slightly procurved, posterior row recurved. Eyes: AME 0.68 (round), ALE 0.85 (ovoid), PME 0.54 (ovoid), PLE 0.52 (ovoid). Interocular distances: AME-AME 0.34, PLE- PLE 2.16, ALE-ALE 1.69, PME-PME 1.34, ALE-PLE 0.20, AME-ALE 0.19, AME-PME 0.06, ALE-PME 0.27, PME-PLE 0.20, AME-PLE 0.57. **EI**(AME)3.61, **EI**(ALE) 4.52.

***Chelicerae*** (Fig. 17A–D). Dorsal length 11.48, dorsal width 4.79, lateral width 67.46. Fang curve length 8.86. Prolateral surface (Fig. 17B): reddish brown, darker distally and paler proximally, long brown fine setae at lower surface, darker and longer at lowest portion above teeth. Intercheliceral pegs are absent but with rows of stiff setae. Retrolateral surface (Fig. 17A): integument, upper 2/3 dark brown, lower surface reddish brown to amber, flat whitish scales (**SC**a), fine, white, cottony acicular (**SC**b), and long, strong tactile setae (**TS**) covering dorsal and upper retrolateral surface, greater anteriorly, patch of fine and long, mostly slightly curved setae at posterior area of lower retrolateral surface, patch of very fine straight setae at anterior area of retrolateral surface, proximomedial setae needle form, very sparse. Long pale reddish brown brush of setae, ventrally. Teeth 16 (including 4 uprooted and 5 smaller teeth) (0.13–0.51), mesoventral denticles dense, ~ 77, on three or four rows. Lyrate region (Fig. 17C, D): 234–245 strikers on six or seven horizontal rows, unordered. Strongest and longest strikers on lower rows. Primary rows ~ 11 (0.86–0.95) long and dark brown, with long, curved filiform ends pointed distally. Secondary rows ~ 140 (0.19–0.67) dark and lanceolate with short curved filiform ends pointed downward. Tertiary rows ~ 89 (0.12–0.15) short, pallid, and needleform. Long, pallid pseudostrikers present ventrally.

***Maxillae*** (Fig. 17E, F). Prolaterally planoconvex, anterior lobe well pronounced, cuspules on inner basoventral surface ~ 365. Stridulatory organ (Fig. 17F): ~ 370 bacilliform rods, in nine or ten rows, form a long and dense ovoid patch, proximally truncated, distally mildly pointed (3.47 long, 1.71 high). Short bacilliform rods 0.21–0.51, with pointed ends. Longer rods 0.49–0.84, paddle-shaped, with paddle blades 0.23–0.36 long and pointed ends, with thick, strong shafts slightly curved outward located at lowest rows. Paddles are flattened perpendicularly to maxillary surface. Total number of rods with paddle blades ~ 40, ~ 10 of them have well-defined paddle blades. Setation: **TS**, brown setae, with upper part paler, present dorsally and ventrally, longest ventrally. Proximodorsal surface with erect pallid fine setae. Lyrate patch surrounded by fine setae. Above maxilla suture with two rows of ~ 24 stiff dark TS. **SC**, flat whitish scales covering dorsal surface.

***Labium and sternum***. Labium (Fig. 16D) length 2.76, width 3.45, integument reddish brown, paler anteriorly. Labial cuspules ~ 759 (0.05–0.08), dark, at apical 1/3. Setation: **TS**(a), long, brown with filiform ends, covering labium anteriorly and laterally except on cuspule cluster, longer and greater at anterior edge, all pointing anteriorly. **TS**(b), dark, spiniform, on posterior surface. Sternum (Fig. 16C) length 8.04, width 7.88, integument pale brown, slightly darker at margin. Posterior sternal corner acuminate, lateral corners weakly acuminate. Labiosternal sigilla ovoid, 1.36 long and 0.49 wide, 1.15 apart. Sternal sigilla three pairs, ovoid. Anterior pair 0.48 long, 0.30 wide, 4.60 apart, and 0.67 away from sternal margin adjacent to coxa I. Median pair 0.48 long, 0.30 wide, 4.92 apart, and 0.52 away from sternal margin adjacent to coxa II. Posterior pair 1.64 long, 0.55 wide, 1.90 apart, and 1.38 away from sternal margin adjacent to coxa III. Setation: **TS**(a), long and short dark spiniform setae on entire sternum, less concentrated on middle surface. **TS**(b), dark and pallid, short spiniform with filiform ends, at sternal margin. **SC**, grayish brown flat scale mat covering entire sternum.

***Abdomen and spinnerets***. Abdomen 23.55 long, 16.33 wide, ovular elongated, integument pale brown. Pedicel 1.87, brown, dorsally striated. Setation: **TS**(a), long brown setae, paler apically, covering entire abdomen except book lungs and epigynal plate, shorter ventrally. Book lungs covered with short brown spiniform setae. **TS**(b), dark short paddle-like, covering entire epigynal plate densely, intermixed with semi-transparent thin and short sensilla, and long spiniform setae anteriorly. **SC**, flat grayish brown scales, covering entire area, darker on epigynal plate. All setae pointing distad. Spinnerets: PMS 2.40 long, 0.84 wide. PLS 10.76 long (ant. 3.72, mid. 3.08, pos. 3.96), 4.08 wide (ant. 1.68, mid. 1.32, pos. 1.08). Setation: **TS**(a), long brown setae, paler apically, covering dorsally and laterally. **TS**(b), dark short paddle-like, covering ventrally. Spigots present on all segments ventrally. **SC**, flat grayish brown scales, sparse, present dorsally.

***Genitalia***. Spermathecae unilobed, not fused. Lobe length 1.25, width 0.93, basal width 1.10, very broad and short tombstone-shaped spermathecal lobe with rounded ends and almost parallel lateral margins (Fig. 18A). Entirely sclerotized, greater apically and weaker basally. Lobes close to each other, separated by 0.07. Epigastric fold 3.96 long.

***Legs***. Leg formula: I, IV, II, III. **RF**~ 104.28., **LLI** (I) 24.43, **LLI** (IV) 21.84, **DLI** (I) 23.37, **DLI** (IV) 20.48, (**MI** (IV) 126.80, **TI** (IV) 50.92. Leg lengths total (fem., pat., tib., met., tar./cym.): Palp 32.07 (10.72, 6.28, 7.92, n/a, 7.15), Leg I 61.35 (16.42, 10.08, 14.29, 11.65, 8.91) Leg II 48.54 (13.13, 8.06, 10.90, 10.10, 6.35), Leg III 42.60 (11.33, 6.30, 8.48, 10.35, 6.14), Leg IV 58.83 (15.07, 7.66, 12.39, 15.71, 8.00). Leg lateral widths (fem., pat., tib., met., tar./cym.): Palp (3.95, 2.50, 2.68, n/a, 0.93), Leg I (4.31, 3.50, 3.02, 2.49, 1.67), Leg II (4.13, 3.28, 2.54, 1.78, 1.41), Leg III (3.69, 3.05, 2.66, 1.74, 1.33), Leg IV (4.06, 3.11, 2.86, 1.61, 1.21). Leg dorsal widths (fem., pat., tib., met., tar./cym.): Palp (2.67, 2.42, 2.46, n/a, 1.70), Leg I (3.42, 3.66, 3.04, 2.31, 1.91), Leg II (3.20, 2.94, 2.51, 2.04, 1.73), Leg III (3.54, 2.60, 2.45, 1.89, 1.64), Leg IV (3.34, 2.79, 2.62, 1.75, 1.55). Tarsus I–V with transverse weakening, tar. I and tar. II more anteriorly, tar. III and tar. IV medially.

***Leg setation* (*femora to tarsi*) *and spines***. Setation: **TS**(a), long and short brown spiniform setae, paler on upper part, covering all over palp and legs except to median to lower prolateral palpal femur surface and leg I and II prolateral femora, longest on palpal tibia and ventral palpal femur, and all leg femora, tibiae, metatarsi, and tarsi. Leg retrolateral femur IV with stout setae. All pointing distad but erect on all ventral femora. **TS**(b), dark paddle-like setae, present on palp (prolateral to ventral distal femur, patella, and proximal tibia), leg I (dorsolateral and ventral femur, ventral to prolateral patella, and proximal tibia), leg II (prolateral and ventral femur, patella, and proximal tibia), and leg III and IV (ventral femora). Very dense on ventral femora I and II and prolateral tibia I and femora II. **FS**, prolateral femur I with a dense field of short sword-like TS (Fig. 21F), similar to TS(b) but shorter and with pointed ends. **SC**, reflective, flat, grayish brown scales, pale brown on all dorsal patellae, covering all legs and palp except prolateral palpal femur proximally, which leaves an ovoid smooth surface. Other sensory setae: **ETB**, short brown setae in two thin inverted L-shaped clusters, starting from basolateral to dorsal Met I to II, and a single cluster on Met III to IV. **TB**(a), long and short filiform TB intermix with ETB in two rows, longest dorsally. **TB**(b) rows of unordered clavate TB, varying in size, present to all tarsi, and intermix with tarsal ETB. **CHS**, tiny pale brown translucent erect sensilla tapering apically, present on palpal and all leg femora to tarsi, and also intermixes with tarsal and metatarsal scopulae. Spines: (dorsal-dorsoprolateral-dorsoretrolateral-ventral): Met I (0-0-0-1), Met II (0-0-0-3). Met III (0-1-1-3). Met IV (0-1-1-3).

***Coxae and trochantera***. Coxae: Palp coxa (see *Maxillae*). Lengths (coxa I, II, III, IV) 8.42, 7.28, 5.72, 6.12. Widths (coxa I, II, III, IV) 4.40, 4.04, 3.88, 4.28. Setation: **TS**, long brown setae dorsally and ventrally, strong and short spiniform setae prolaterally on all coxae. **SC**(a), flat scales covering ventral to retrolateral 1/2. **SC**(b), white cottony acicular covering dorsal of all coxae. Patches of fine setal fringe present laterally on coxae, intermixed with short spiniform setae. Coxae I–IV have rows of short semi-translucent bristles, prolaterally, denser on Coxae I and II. Trochantera: Lengths (troch. palp, I, II, III, IV) 2.84, 4.32, 3.88, 3.12, 3.64. Widths (coxa palp, I, II, III, IV) 2.92, 3.76, 3.20, 3.04, 3.52.

***Scopulae and claws***. Scopulae (left): Palp tarsus undivided but parted. Tarsus I, entire, but intermixed with longitudinal one or two rows of very sparse short spiniform setae. Tarsus II, entire, but intermixed with very sparse longitudinal one or two rows of short spiniform setae. Tarsus III, entire, but intermixed with longitudinal two or three rows of strong long setae. Tarsus IV, entire, divided by four rows of strong long setae. All tarsi with a bald spot ventrobasally. Met. I covered almost all ventral surfaces, entire, but with longitudinal one or two rows of very sparse long setae. Met. II, covered almost all ventral surface, entire, but with longitudinal one or two rows of very sparse long setae. Met. III, covered 4/5 distally, entire, but with longitudinal one or two rows of very sparse long setae. Met. IV, covered 3/4 distally, divided by two or three rows of strong long setae. Claws: pair of claws present on all leg tarsi with one to three teeth on each claw. Tarsus IV with a short unpaired third inferior claw.

***Color in life***. Females are mildly dichromatic (Fig. 11B), with dark brown on carapace, abdomen, palp, and all legs, contrasted by dark femora on all legs and palp. Dark brown legs (except femora) are topped with pale brown setae, sparsely hirsute. Legs and abdomen reflect (by scales) a deep, mild purplish blue sheen. Generally, uniformly dark with a mild bluish sheen after ecdysis, which fades in a few days. Coloration changes, becoming pale brown, as the exoskeleton ages before ecdysis.

###### Natural history and distribution.

**Figure 19.**
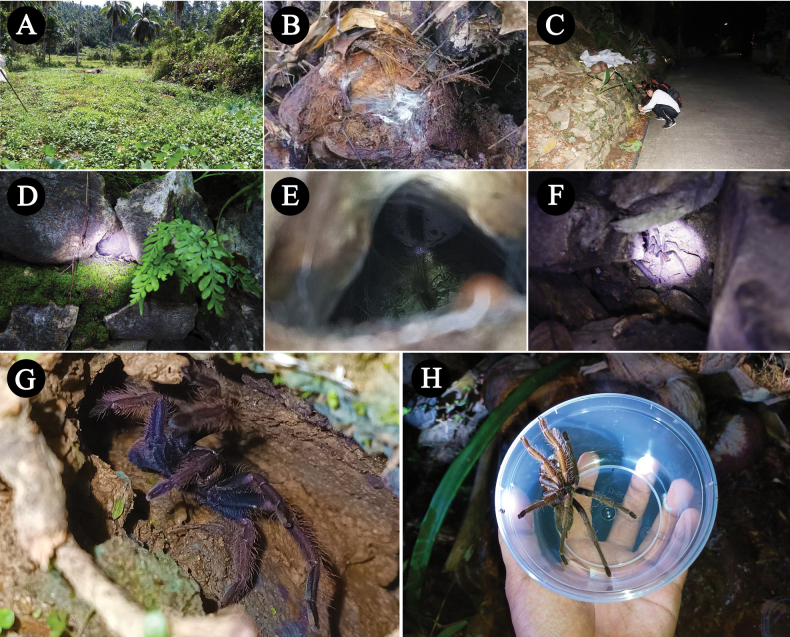
*Selenobrachysustromsupasius* sp. nov. **A**–**H** habitat and burrows on metamorphosed limestone (marble) piles and crevices and piles of coconut husks **E** female with egg sac **E**–**H** paratype females, in situ.

Mature males were collected in September, and females with egg sacs (Fig. 19E) were found in February. Burrows, not self-dug, were found under piles of coconut husks (Fig. 19B, G) piles and crevices of metamorphosed limestones (marble) at the roadside embankments (Fig. 19C–F). They are also found in the beach forest of Bon Bon Beach in the northwestern coast of Romblon Island. The spiders were found on the burrow entrance at night, waiting for prey to ambush. This species is known only to occur in Romblon Island, Philippines (Fig. 22).

**Figure 20.**
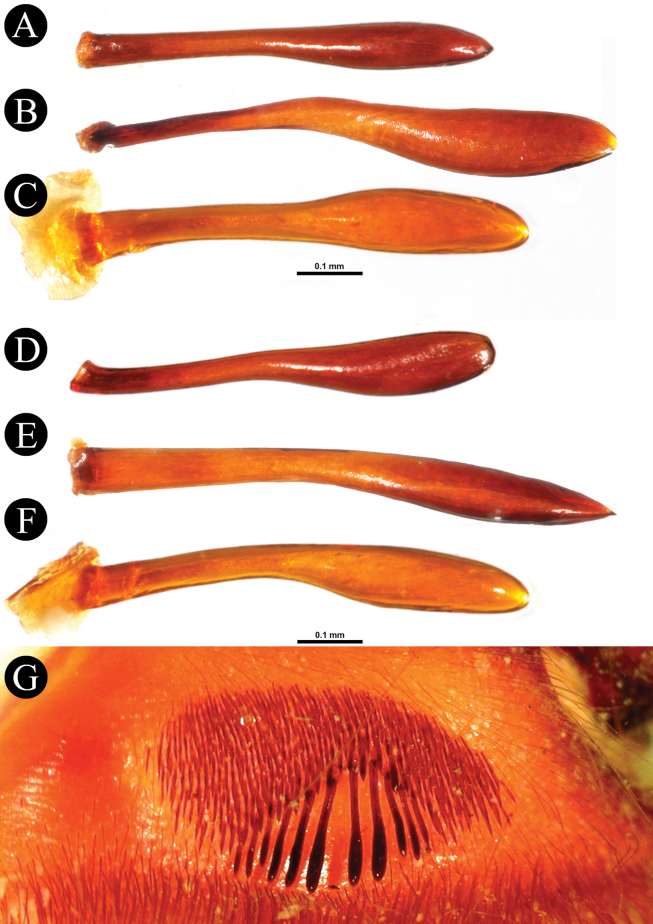
Largest stridulating setae on maxillary lyra **A**–**C** dorsal view **D**–**F** prolateral view **A**, **D***Orphnaecus* sp. **B**, **E***Selenobrachysphilippinus* comb. rest **C**, **F***Chilocosmiadichromata* comb. rest **G***Orphnaecuspellitus*, syntype ♀, lyra on prolateral maxillary surface.

**Figure 21.**
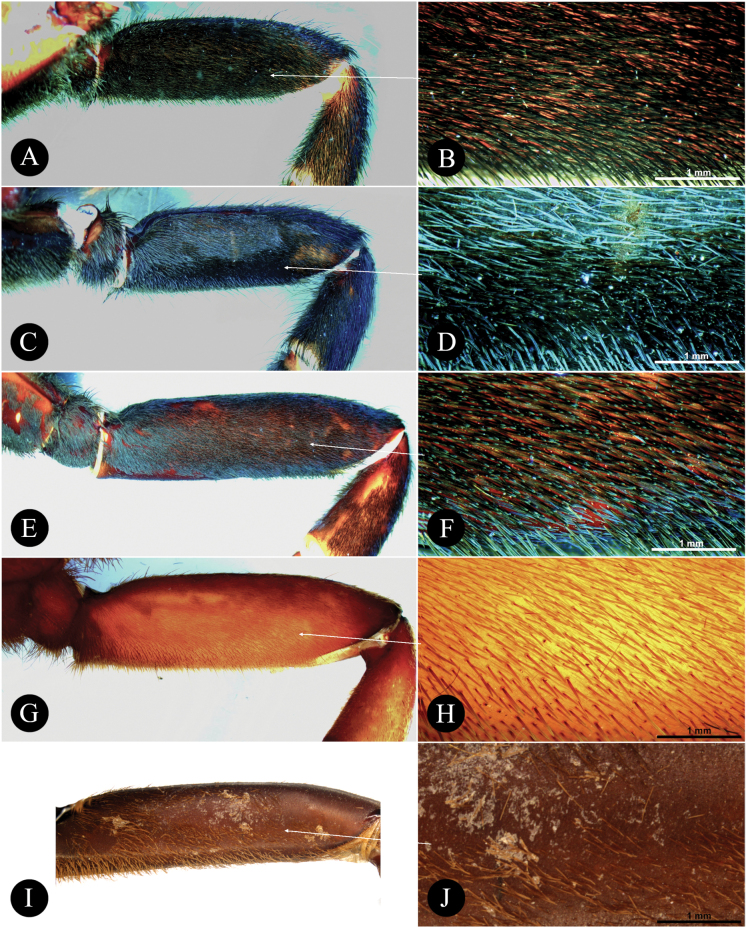
Femoral Setation (FS) on left prolateral femur I **A**, **B***Orphnaecuskwebaburdeos*, ♀, UST-ARC 0064 **C**, **D***Orphnaecus* sp.’ L3’, ♀, UST-ARC 0134 **E**, **F***Selenobrachysustromsupasius* sp. nov., paratype ♀, UST-ARC 0005 **G**, **H***Selenobrachysphilippinus* comb. rest., ♀, UST-ARC 0112 **I**, **J***Chilocosmiadichromata* comb. rest., ♂, SMNS Aran-004182 **B**, **D**, **F**, **H, J** magnified view of the left femur I median prolateral surface.

**Figure 22.**
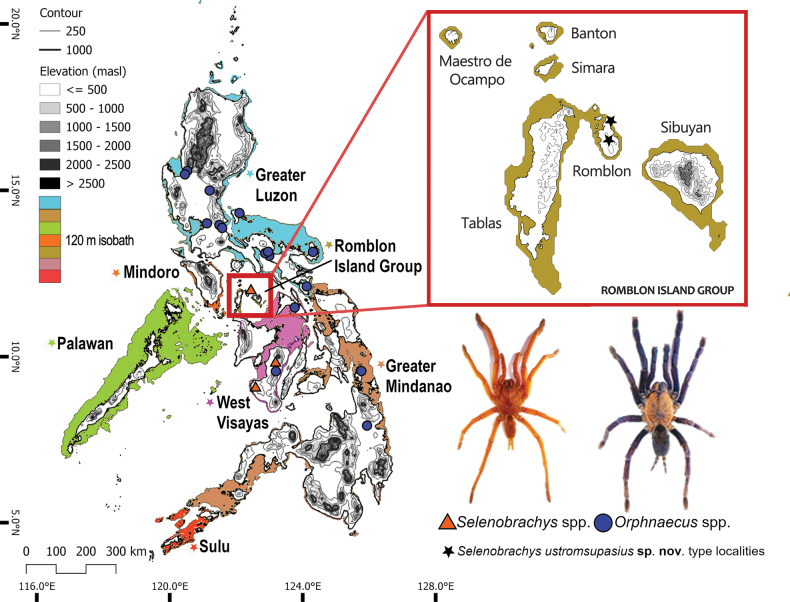
The seven major Philippine biogeographic regions based on the PAIC (Pleistocene Aggregate Island Complex) paradigm and the distribution map of valid species and published records of *Orphnaecus* (blue circles) and *Selenobrachys* (orange triangle). Inset: type localities of *Selenobrachysustromsupasius* sp. nov.

###### Etymology.

The specific epithet is a masculine adjective derived from the combined names of the collaborating academic institutions and an organization of the project in which this study is involved, namely, the University of Santo Tomas (UST), Romblon State University (RSU), Mindanao State University-Iligan State University (MSU-IIT), University of the Philippines-Diliman (UPD), and the Philippine Arachnological Society, Inc. (PASI), attached with the Latin suffix -*us*.

## ﻿Discussion

Morphology and gene sequences are the two most important data that can be used in taxonomy and systematics. Taxonomic information acquired from the morphological analysis is, in many cases, being questioned and subjected to debate among taxonomists due to their differences in interpretations and opinions (Sites and Marshall 2004; Wiens 2004; Will and Rubinoff 2004; Dayrat 2005; Vences et al. 2013). The gene sequencing method appears to be ideal as it is very specific, but this method still needs to be supported with a physically observable characteristic to affirm its validity. The review of the taxa in this study is based on the combined information of morphology and DNA sequences with insights into their biogeography.

### ﻿Morphological basis on the validity of *Selenobrachys* stat. rev. and *Chilocosmia*. stat. rev.

*Selenobrachys* and *Chilocosmia* were considered junior synonyms of *Orphnaecus* by West et al. (2012) based on the characters used in their cladistic analysis. They considered the oval proximally truncated lyrate patch of *Selenobrachys* an autapomorphy in the *Orphnaecus* clade, but we consider this synapomorphy within *Selenobrachys* with the discovery of additional *Selenobrachys* species (*S.ustromsupasius* sp. nov.) and five other potential undescribed species from other islands of Romblon PAIC and West Visayas PAIC known to the authors, which share the same lyrate morphology and spermathecal morphology. Additionally, the maxillary lyra of *Selenobrachys* is differentiated from *Orphnaecus* by its short bacilliform rods forming a dense ovoid patch that is truncated proximally and mildly tapering distally, and the rows of paddle-shaped lyrate bacillae at the lowest rows have distinctly thicker shafts prolaterally whereas the largest ones in the lowest row have a more pointed tip in prolateral view (Figs 7G, 13F, 17F, 20B, E). The short bacilliform rods on the lyra of *Orphnaecus* form a reniform patch (West et al. 2012), less dense, with rows of clavate bacillae that have elongated paddles and longer and stouter shafts and the largest have a rounded tip (Fig. 20A, D).

The secondary rows of cheliceral strikers of *Selenobrachysphilippinus* comb. rest. were scored similarly (lanceolate in shape) with *Orphnaecus* specimens in the same cladistic study (West et al. 2012). However, our analysis shows that all *Selenobrachys* materials we examined have secondary rows of cheliceral strikers that are shorter, slightly curved, and spiniform in shape (Figs 8C, 13C, D, 17C, D), while *Orphnaecus* has straighter and longer lanceolate secondary strikers (West et al. 2012). West et al. (2012) also noted < 50 strikers in *Orphnaecus* and *Phlogiellus*, as one of the synapomorphies of the tribe Yamiini Kishida, 1920, but we recorded > 150 strikers (excluding tertiary strikers) on our *Selenobrachys* specimens. We also noticed that the very short needle-form tertiary rows of cheliceral strikers in *Selenobrachys* are greater in number (100–200) (Figs 8C, 13C, D, 17C, D) which corresponds with the very dense lyrate patch of the genus.

West et al. (2012) mentioned the similarity in spermathecal morphology (tombstone-shaped) of *S.philippinus* comb. rest. to their *Orphnaecus* specimens (QM S83782, S83783) from Masbate Island. With the absence of an illustration of the spermathecae of their *Orphnaecus* species, we can assume that it is just a resemblance, considering that the spermathecal morphology of *O.kwebaburdeos* (Fig. 18C) is almost in a tombstone-shape but still fits our description of *Orphnaecus*, in having longer spermathecal lobes with more concave prolateral margins which slightly pointing inward apically, creating asymmetry to the lobe (Fig. 18C, D). All *Selenobrachys* specimens we examined have broad and short spermathecal lobes, in a tombstone shape, almost symmetrical, with lateral margins almost parallel and very mildly or not concave prolaterally, not converging distally, and the apically rounded ends not pointing inward (Fig. 18A, B).

The male of *S.philippinus* comb. rest. has been unknown since the original description of the species. Fortunately, we were able to collect new specimens from the type locality in Mambucal, Negros Island, which included a single adult male. Additional *S.philippinus* male specimens were examined from SMNS. The palpal tibiae of male *Selenobrachys* are distinctly longer and cylindrical (Figs 9A–D, 15A, B), while in the sister genera, the palpal tibiae of males are proximally incrassate and tapering distally. Additionally, *Orphnaecus* males have a distinct, very dense, very long dorsal brush on the palpal patellae (Fig. 15A; West et al. 2012) which are also present on the palpal tibiae in some species.

West et al. (2012) mentioned the synapomorphy of Yamiini of having a single retrolateral keel on the embolus of males. Based on our analyses of the palpal organ morphology of male *Orphnaecus* and *Selenobrachys* specimens, the retrolateral keel is actually an extremely long prolateral superior keel, following the work of Bertani (2000), for which we adopted the structures on the embolus of males based on the position of the keels. The retrolateral keel emerges retrolaterally from the apex of the embolus to the rear (Bertani 2000), which is not the case in *Orphnaecus* and *Selenobrachys*. However, further studies on homologizing the structures of male palpal organs of Selenocosmiinae are necessary.

*Selenobrachys* species have relatively stouter legs dorsally (except for *O.pellitus* which also has stout legs caused by troglomorphism). We also explored the setation field on the prolateral surface of femur I, which we herein call femoral setation (FS): we found that *Selenobrachys* species have a field of short sword-shaped setae with a narrow base (Fig. 21E–H), while *Orphnaecus* have longer needle-form setae (Fig. 21A–D). However, it is suggested to further study the ultramorphology of the femoral setation under a scanning electron microscope.

*Chilocosmia* stat. rev. was synonymized based on the synapomorphic characters of *Orphnaecus* (West et al. 2012). Schmidt and von Wirth (1992) described the lyra of this genus as an arcuate strip of short rods consisting of an arcuate row of clavate bacillae. The largest bacillae are stronger (Fig. 21C, F) and the short rods are less dense (< 200) than in *Orphnaecus* and *Selenobrachys* stat rev. (> 300; if not absent or not rudimentary which are consequences of phenotypic plasticity). In addition, the morphology of the embolus with its strongly twisted tegulum and its missing BL and the missing long PS keel of the male of *C.dichromata* comb. rest. are not found in any described species of *Orphnaecus* and *Selenobrachys* stat. rev. (Fig. 5A–E). The dense long palpal brush on the dorsal male palpal patellae is also missing in *C.dichromata* comb. rest. (Fig. 5D). The female spermathecae of *C.dichromata* comb. rest. have reduced ends or are distally converging as in *Orphnaecus*, but not concave prolaterally and not pointing inwards (Schmidt and von Wirth 1992). The median carapace line in *Orphnaecus* is not unique to the genus but is also present in other Yamiini genera.

### ﻿Genetic data sequencing and phylogenetic investigation

Most barcoding studies in animals utilize the cytochrome c oxidase subunit I (COl) gene due to its key characteristics such as universality and rapid substitution at the third codon position; the expansion of genetic databases has established a firm basis for utilizing this gene in the identification of specimens. However, the assessment of COI and 16S for DNA barcoding of farmland spiders from previous studies showed the potential efficiency of rRNA genes in identifying genetic species boundaries (Hamilton et al. 2011, 2016; Turner et al. 2018; Briggs et al. 2023) and their capability in identification during high-throughput experiments using various metabarcoding protocols (Wang et al. 2017; Vences et al. 2005). In this study, COI and rRNA genes have provided a preliminary understanding of the relationships between known Philippine tarantula species. Although the COI phylogenetic tree received very low bootstrap support, the assessment of genetic distances and support from morphology collectively contribute to a more robust depiction of the distinction between *Orphnaecus*, *Selenobrachys* stat. rev., and *Chilocosmia* stat. rev. This first molecular analysis for Philippine tarantula spiders is an important initiative for more molecular studies on these taxa.

Significantly, the arrangement of species within clades on the phylogenetic trees (Fig. 1A, C) supports the morphological synapomorphies, particularly evident in instances like *S.philippinus* comb. rest. and the newly described species *S.ustromsupasius* sp. nov., across phylogenetic trees of both genes. The analysis of genetic distances offers quantitative insights into the extent of genetic similarity or divergence among different species that were initially delimited with morphological differences (Suppl. material 2). The observed pairwise distance values for both genes provide additional evidence of the relationships between genus and species. Earlier studies showed the differences in percentage values of intraspecific distance that was much lower than interspecific genetic distance (Powell and Caccone 1989; Cádiz et al. 2018; Ma et al. 2019, 2022). This supports the results of this study that the lower percentage differences between intraspecies and the higher percentage differences among the interspecies in the genetic distances of tarantula spiders indicate greater genetic similarity and divergence, respectively. The comparison of these values across species aligns well with both the morphological synapomorphies and the phylogenetic tree topology.

### ﻿The Pleistocene Aggregate Island Complexes (PAICs) paradigm

Unlike most of the islands in Southeast Asia and Oceania, the Philippine Arc did not evolve from the Sahulian biogeographic realm nor the Eurasian plate (Queaño et al. 2020). The present islands of the Philippines, except Palawan, originated from the Philippine Islands arc system, which evolved as a result of subduction of the ocean floor along the trenches surrounding the Philippines during the Cretaceous period (Yumul et al. 2008; Aurelio et al. 2013). This paleogeographic history of the Philippines explains the existence of numerous flora and fauna unique to this country (Jones and Kennedy 2008).

Islands in the Philippine archipelago have been grouped and interconnected in the past due to Pleistocene sea level drops during the glacial periods (Heaney 1991; Heaney et al. 1998). These major island groups are known as Pleistocene Aggregate Island Complexes (PAICs) (Esselstyn and Brown 2009) and are based on the present 120 m isobath (Fig. 22). This formation has created the seven major biogeographical regions—namely, the Luzon PAIC (Greater Luzon), Mindanao PAIC (Greater Mindanao), West Visayan PAIC, Palawan PAIC, Sulu PAIC, Mindoro PAIC, and Romblon PAIC (Romblon Island Group; RIG) (Fig. 22). This concept may have facilitated the colonization and diversification of Filipino tarantulas. Tarantula spiders, like most mygalomorphs, have a limited mode of dispersion that is limited only to ground movement, making them less mobile than the araneomorph spiders. The archipelagic setting of the Philippines could have promoted rich diversification and unique evolutionary radiation for tarantula spiders, which is evident in the rich terrestrial fauna of the country (Heaney et al. 2016; Oaks et al. 2019; Pitchay and Torrentira 2022).

The results of our morphological and molecular analyses support the resurrection of the genus *Selenobrachys*, but we recognize its close relationship to *Orphnaecus* as a sister genus. Biogeographically, *Selenobrachys* might be limited to the Romblon Island Group + West Visayan PAIC. Citizen science sightings known by the first author provide the presence of the genus in other West Visayan + Romblon PAIC islands (except Masbate Island) which could still have five or more island-endemic new undescribed *Selenobrachys* species. Given its current distance from the rest of West Visayan PAIC islands, Masbate could have fragmented earlier from the rest of West Visayan PAIC islands before the diversification of the genus, hence explaining their potential absence on the island. We presume that the genus evolved from *Orphnaecus-Selenobrachys* ancestor from Luzon when West Visayan PAIC was still connected to Greater Luzon and became isolated upon its fragmentation. Molecular dating is needed to test this hypothesis. *Orphnaecus* is one of the most widespread theraphosid taxa in the Philippines, but they might not be able to colonize Mindoro, Romblon Island Group, Sulu Island Group, and the Palawan realm, owing to their different land mass origins. Proto-*Orphnaecus* may have ridden the proto-Luzon mass while rafting northward to its present position through the Philippine Arc System. The presence of *Orphnaecus* in West Visayan PAIC may be the result of recolonization after the first colonization and isolation of *Selenobrachys* when the islands reconnected through the oscillating water level during the Late Pleistocene, but during that time Romblon PAIC may not be reconnected to Panay thus explains the absence of *Orphnaecus* in the said island group, and Panay became their terminal expansion westward.

The placement of the Papuan species, *C.dichromata* comb. rest., in *Orphnaecus* is biogeographically suspicious. Morphologically, this species is distinct from the Philippine *Orphnaecus* species for having an arcuate strip of short rods in lyra (Schmidt and von Wirth 1992), with slight differences in the structure of the spermathecae and a vastly different genital morphology of the males. The Philippine Arc System and Papuan geographical disjunct is dramatic, although, the most recent possible land connection may have happened during the Late Oligocene to Late Miocene (~ 25–10 mya) when the Philippine Arc and Halmahera Arc may have aligned together creating a path for dispersal (Kalkman et al. 2018; Bocek and Bocak 2019). Genetic investigation by Autelin et al. (2021) on the freshwater gastropod subfamily, Miratestinae, reveals migration from Sahul to the Indo-Australian Archipelago, the Philippines, and the West Pacific Islands in the Early Miocene. It is interesting to test the same with Selenocosmiinae if they have utilized the same routes, which can only be done with the availability of more genetic data from the Sahulian and Sundaic taxa.

## ﻿Conclusions

Evidence from morphological and molecular analyses as well as biogeographical insights revealed that the taxonomic status of the genus *Selenobrachys* is indeed valid. We therefore remove *Selenobrachys* Schmidt, 1999 from the synonymy of *Orphnaecus* Simon, 1892, and we restore the genus to its valid genus status, with its type species, *Orphnaecusphilippinus*, original combination restored becoming *Selenobrachysphilippinus* comb. rest., and we describe a second species for the genus, *Selenobrachysustromsupasius* sp. nov., from Romblon Island. Furthermore, we recognize the close relationship of *Selenobrachys* and *Orphnaecus* based on the synapomorphic characters within the tribe Yamiini, thus placing *Selenobrachys* in this clade. As discussed above, we also restore the genus *Chilocosmia* Schmidt & von Wirth, 1992 stat. rev. and its type species *Chilocosmiadichromata* Schmidt & von Wirth, 1992, comb. rest. based on morphological, molecular, and biogeographic points. The number of genera within the subfamily Selenocosmiinae has now increased to 13, and the theraphosid fauna of the Philippines is now five genera and 14 species. Studying the biogeography of animals in the Philippines that are mostly limited to ground dispersal, like most mygalomorph spiders, may help us better understand the biogeographic history of the Philippines. Molecular analysis should always be supported by morphology (and vice versa) and other available evidence because delimitation based only on genetic divergence is subjective and has no universal standards and the variations in morphology do not always warrant species boundaries.

## Supplementary Material

XML Treatment for Selenocosmiini

XML Treatment for Chilocosmia

XML Treatment for Chilocosmia dichromata

XML Treatment for Yamiini

XML Treatment for Orphnaecus

XML Treatment for Selenobrachys

XML Treatment for Selenobrachys philippinus

XML Treatment for Selenobrachys ustromsupasius

## ﻿Additional information

### Author ORCIDs

Darrell C. Acuña https://orcid.org/0000-0002-2958-8763

Maria Mikaela U. Dumbrique https://orcid.org/0000-0003-3592-2585

Maricel C. Ranido https://orcid.org/0000-0002-4083-5489

Lorenz Rhuel P. Ragasa https://orcid.org/0000-0001-8581-9872

Anna Beatriz R. Mayor https://orcid.org/0000-0002-2256-1357

Mary Jane A. Fadri https://orcid.org/0000-0001-6906-0157

Volker von Wirth https://orcid.org/0000-0003-2868-0768

Myla R. Santiago-Bautista https://orcid.org/0000-0001-9173-7320

Leonardo A. Guevarra Jr https://orcid.org/0000-0002-2031-0300

### Data availability

All of the data that support the findings of this study are available in the main text or Supplementary Information.

## Supplementary materials

Supplementary material 1 List of all new genetic sequences deposited in GenBank Data type pdf https://binary.pensoft.net/file/1295978This dataset is made available under the Open Database License (http://opendatacommons.org/licenses/odbl/1.0/). The Open Database License (ODbL) is a license agreement intended to allow users to freely share, modify, and use this Dataset while maintaining this same freedom for others, provided that the original source and author(s) are credited.Darrell C. Acuña, Myla R. Santiago-Bautista, Leonardo A. Guevarra Jr

Supplementary material 2 Percent pairwise distances of all sequences of CO1 and 12S–tRNA-Val–16S Data type pdf https://binary.pensoft.net/file/1295979This dataset is made available under the Open Database License (http://opendatacommons.org/licenses/odbl/1.0/). The Open Database License (ODbL) is a license agreement intended to allow users to freely share, modify, and use this Dataset while maintaining this same freedom for others, provided that the original source and author(s) are credited.Darrell C. Acuña, Myla R. Santiago-Bautista, Leonardo A. Guevarra Jr
