# Supplementary material for: ﻿Taxonomic revalidation of Selenobrachys Schmidt, 1999 and Chilocosmia Schmidt & von Wirth, 1992 based on morphological and molecular analyses (Araneae, Theraphosidae), with the description of a new species from Romblon Island, Philippines
Source: Zookeys. 2025 Mar 31;1233:139–93. doi: 10.3897/zookeys.1233.128056 (PMC11976310; doi:10.3897/zookeys.1233.128056)
Supplement: Supplementary material 1 — List of all new genetic sequences deposited in GenBank [file zookeys-1233-139_article-128056__-s001.pdf]

Supplementary data 1. List of all new genetic sequences deposited to GenBank

Species	Field No.	Voucher	GenBank Accesion: COI	GenBank Accesion: 12S–tRNA-Val–16S
Aphonopelma seemanni	AS1	–	–	PP794542
Chilocosmia dichromata comb. rest.	–	SMNS Aran-004162	PP726664	–
Chilocosmia dichromata comb. rest.	–	SMNS Aran-004163	PP726662	–
Chilocosmia dichromata comb. rest.	–	SMNS Aran-004182	PP726663	–
Chilocosmia dichromata comb. rest.	–	SMNS Aran-004183	PP726660	–
Chilocosmia dichromata comb. rest.	–	SMNS Aran-004185	PP726661	–
Orphnaecus kwebaburdeos	QB1-03	UST-ARC 0061	–	PP794543
Orphnaecus kwebaburdeos	QB1-06	UST-ARC 0064	PP778328	–
Orphnaecus kwebaburdeos	QB1-07	UST-ARC 0065	–	PP794544
Orphnaecus kwebaburdeos	QB1-10	UST-ARC 0068	–	PP794545
Orphnaecus kwebaburdeos	QB2-07	UST-ARC 0081	PP778329	–
Orphnaecus pellitus	CSL01-01	UST-ARC 0031	PP778304	PP794546
Orphnaecus pellitus	CSL01-02	UST-ARC 0032	PP778305	PP794547
Orphnaecus pellitus	CSL01-08	UST-ARC 0038	PP778306	PP794548
Orphnaecus pellitus	CSL05-01	UST-ARC 0052	PP778313	PP794549
Orphnaecus pellitus	CSL05-05	UST-ARC 0056	–	PP794550
Orphnaecus pellitus	CSL05-06	UST-ARC 0057	PP778314	PP794551
Orphnaecus pellitus	CSL05-07	UST-ARC 0058	PP778315	PP794552
Orphnaecus sp. 'L1'	UPLG1-01	UST-ARC 0105	PP778341	PP794553
Orphnaecus sp. 'L1'	UPLG1-02	UST-ARC 0106	PP778338	–
Orphnaecus sp. 'L1'	UPLG1-03	UST-ARC 0107	PP778342	PP794554
Orphnaecus sp. 'L1'	UPLG1-04	UST-ARC 0108	PP778339	–
Orphnaecus sp. 'L1'	UPLG1-05	UST-ARC 0109	PP778340	–
Orphnaecus sp. 'L1'	LS01-01	UST-ARC 0084	PP778318	–
Orphnaecus sp. 'L2'	QR1-01	UST-ARC 0102	PP778330	–
Orphnaecus sp. 'L2'	QR1-02	UST-ARC 0103	PP778331	PP794555
Orphnaecus sp. 'L2'	LS02-02	UST-ARC 0086	PP778319	–
Orphnaecus sp. 'L2'	LS03-01	UST-ARC 0088	PP778320	–
Orphnaecus sp. 'L2'	LS04-08	UST-ARC 0097	–	PP794556
Orphnaecus sp. 'L2'	LS04-09	UST-ARC 0098	PP778321	–
Orphnaecus sp. 'L2'	LS04-11	UST-ARC 0100	PP778322	–
Orphnaecus sp. 'L2'	LS04-12	UST-ARC 0101	PP778323	–
Orphnaecus sp. 'L3'	CSL02-01	UST-ARC 0130	PP778307	PP794557
Orphnaecus sp. 'L3'	CSL02-02	UST-ARC 0131	–	PP794558
Orphnaecus sp. 'L3'	CSL04-01	UST-ARC 0134	PP778308	PP794561
Orphnaecus sp. 'L3'	CSL04-02	UST-ARC 0135	PP778309	PP794559
Orphnaecus sp. 'L3'	CSL04-03	UST-ARC 0136	PP778310	PP794560
Orphnaecus sp. 'L3'	CSL04-04	UST-ARC 0137	PP778311	–
Orphnaecus sp. 'L3'	CSL04-05	UST-ARC 0138	PP778312	–
Orphnaecus sp. 'L4'	CSL06-02	UST-ARC 0142	PP778316	–
Orphnaecus sp. 'L5'	NEPT1-003	UST-ARC 0145	OQ974566	–
Orphnaecus sp. 'L5'	DME22A001.1	PASI ara0014	PP778327	PP794562
Orphnaecus sp. 'L6'	DCA 37	PASI ara0020	PP778317	–
Orphnaecus sp. 'M1'	PBAT1-01	UST-ARC 0146	PP778326	–
Selenobrachys philippinus comb. rest.	NOM1A-01	UST-ARC 0112	PP778324	PP794563
Selenobrachys philippinus comb. rest.	NOM1A-04	UST-ARC 0115	PP778325	PP794564
Selenobrachys philippinus comb. rest.	NOM1A-07	UST-ARC 0118	–	PP794565
Selenobrachys ustromsupasius sp. nov.	R01-01	UST-ARC 0001	–	PP794566
Selenobrachys ustromsupasius sp. nov.	R01-02	UST-ARC 0002	–	PP794567
Selenobrachys ustromsupasius sp. nov.	R01-05	UST-ARC 0005	PP778332	–
Selenobrachys ustromsupasius sp. nov.	R01-09	UST-ARC 0009	PP778333	–
Selenobrachys ustromsupasius sp. nov.	R02-02	UST-ARC 0016	PP778334	PP794568
Selenobrachys ustromsupasius sp. nov.	R02-16	UST-ARC 0030	–	PP794569
Selenobrachys ustromsupasius sp. nov.	TRT-001	UST-ARC 0147	PP778335	–
Selenobrachys ustromsupasius sp. nov.	TRT-002	UST-ARC 0148	PP778336	–
Selenobrachys ustromsupasius sp. nov.	TRT-003	UST-ARC 0149	PP778337	–
