# Supplementary material for: ﻿Taxonomic revalidation of Selenobrachys Schmidt, 1999 and Chilocosmia Schmidt & von Wirth, 1992 based on morphological and molecular analyses (Araneae, Theraphosidae), with the description of a new species from Romblon Island, Philippines
Source: Zookeys. 2025 Mar 31;1233:139–93. doi: 10.3897/zookeys.1233.128056 (PMC11976310; doi:10.3897/zookeys.1233.128056)
Supplement: Supplementary material 2 — Percent pairwise distances of all sequences of CO1 and 12S–tRNA-Val–16S [file zookeys-1233-139_article-128056__-s002.pdf]

Supplementray Data 2.1. Percent pairwise distances between COI Sequences

	CLS01-08	CSL01-01	CSL01-02	CSL05-01	CSL05-06	CSL05-07	QB1-06	QB2-07	LS01-01	UPLG1-01	UPLG1-02	UPLG1-03	UPLG1-04	UPLG1-05
CLS01-08 *Orphnaecus pellitus*														
CSL01-01 *Orphnaecus pellitus*	0.35%													
CSL01-02 *Orphnaecus pellitus*	0.00%	0.35%												
CSL05-01 *Orphnaecus pellitus*	0.35%	0.00%	0.35%											
CSL05-06 *Orphnaecus pellitus*	0.35%	0.00%	0.35%	0.00%										
CSL05-07 *Orphnaecus pellitus*	0.35%	0.00%	0.35%	0.00%	0.00%									
QB1-06 *Orphnaecus kwebaburdeos*	9.07%	8.67%	9.07%	8.67%	8.67%	8.67%								
QB2-07 *Orphnaecus kwebaburdeos*	9.28%	8.89%	9.28%	8.89%	8.89%	8.89%	0.17%							
LS01-01 *Orphnaecus* sp. 'L1'	10.52%	10.11%	10.52%	10.11%	10.11%	10.11%	8.07%	8.28%						
UPLG1-01 *Orphnaecus* sp. 'L1'	10.45%	10.05%	10.45%	10.05%	10.05%	10.05%	6.06%	6.26%	4.19%					
UPLG01-02 *Orphnaecus* sp. 'L1'	10.45%	10.05%	10.45%	10.05%	10.05%	10.05%	6.06%	6.26%	4.19%	0.00%				
UPLG01-03 *Orphnaecus* sp. 'L1'	10.45%	10.05%	10.45%	10.05%	10.05%	10.05%	6.06%	6.26%	4.19%	0.00%	0.00%			
UPLG01-04 *Orphnaecus* sp. 'L1'	10.45%	10.05%	10.45%	10.05%	10.05%	10.05%	6.06%	6.26%	4.19%	0.00%	0.00%	0.00%		
UPLG01-05 *Orphnaecus* sp. 'L1'	10.45%	10.05%	10.45%	10.05%	10.05%	10.05%	6.06%	6.26%	4.19%	0.00%	0.00%	0.00%	0.00%	
QR1-01 *Orphnaecus* sp. 'L2'	10.63%	10.22%	10.63%	10.22%	10.22%	10.22%	8.51%	8.72%	8.02%	7.74%	7.74%	7.74%	7.74%	7.74%
QR1-02 *Orphnaecus* sp. 'L2'	10.63%	10.22%	10.63%	10.22%	10.22%	10.22%	8.51%	8.72%	8.02%	7.74%	7.74%	7.74%	7.74%	7.74%
LS02-02 *Orphnaecus* sp. 'L2'	10.43%	10.02%	10.43%	10.02%	10.02%	10.02%	8.70%	8.91%	7.98%	8.32%	8.32%	8.32%	8.32%	8.32%
LS04-09 *Orphnaecus* sp. 'L2'	10.21%	9.81%	10.21%	9.81%	9.81%	9.81%	8.10%	8.31%	7.62%	7.33%	7.33%	7.33%	7.33%	7.33%
LS04-11 *Orphnaecus* sp. 'L2'	10.73%	10.27%	10.73%	10.27%	10.27%	10.27%	8.79%	9.03%	8.69%	7.65%	7.65%	7.65%	7.65%	7.65%
LS04-12 *Orphnaecus* sp. 'L2'	10.21%	9.81%	10.21%	9.81%	9.81%	9.81%	8.10%	8.31%	7.62%	7.33%	7.33%	7.33%	7.33%	7.33%
LS03-01 *Orphnaecus* sp. 'L2'	10.43%	10.02%	10.43%	10.02%	10.02%	10.02%	8.70%	8.91%	7.98%	8.32%	8.32%	8.32%	8.32%	8.32%
DME22A001.1 *Orphnaecus* sp. 'L5'	10.86%	10.46%	10.86%	10.46%	10.46%	10.46%	7.67%	7.88%	8.05%	8.10%	8.10%	8.10%	8.10%	8.10%
NEPT1-003 *Orphnaecus* sp. 'L5'	9.63%	9.63%	9.63%	9.63%	9.63%	9.63%	8.67%	8.88%	8.85%	8.90%	8.90%	8.90%	8.90%	8.90%
DCA 37 *Orphnaecus* sp. 'L6'	10.18%	10.58%	10.18%	10.58%	10.58%	10.58%	8.32%	8.53%	9.37%	9.99%	9.99%	9.99%	9.99%	9.99%
CSL02-01 *Orphnaecus* sp. 'L3'	9.12%	9.12%	9.12%	9.12%	9.12%	9.12%	4.90%	5.09%	9.58%	8.23%	8.23%	8.23%	8.23%	8.23%
CSL04-02 *Orphnaecus* sp. 'L3'	9.12%	9.12%	9.12%	9.12%	9.12%	9.12%	4.90%	5.09%	9.58%	8.23%	8.23%	8.23%	8.23%	8.23%
CSL04-01 *Orphnaecus* sp. 'L3'	9.12%	9.12%	9.12%	9.12%	9.12%	9.12%	4.90%	5.09%	9.58%	8.23%	8.23%	8.23%	8.23%	8.23%
CSL04-03 *Orphnaecus* sp. 'L3'	9.12%	9.12%	9.12%	9.12%	9.12%	9.12%	4.90%	5.09%	9.58%	8.23%	8.23%	8.23%	8.23%	8.23%
CSL04-04 *Orphnaecus* sp. 'L3'	9.12%	9.12%	9.12%	9.12%	9.12%	9.12%	4.90%	5.09%	9.58%	8.23%	8.23%	8.23%	8.23%	8.23%
CSL04-05 *Orphnaecus* sp. 'L3'	9.12%	9.12%	9.12%	9.12%	9.12%	9.12%	4.90%	5.09%	9.58%	8.23%	8.23%	8.23%	8.23%	8.23%
CSL06-02 *Orphnaecus* sp. 'L4'	7.91%	8.30%	7.91%	8.30%	8.30%	8.30%	6.69%	6.89%	9.41%	9.30%	9.30%	9.30%	9.30%	9.30%
PBAT1-01 *Orphnaecus* sp. 'M1'	9.72%	9.32%	9.72%	9.32%	9.32%	9.32%	5.49%	5.69%	9.57%	8.47%	8.47%	8.47%	8.47%	8.47%
NOM1A-01 *Selenobrachys philippinus* comb. rest.	12.98%	12.98%	12.98%	12.98%	12.98%	12.98%	12.74%	12.97%	13.07%	13.28%	13.28%	13.28%	13.28%	13.28%
NOM1A-04 *Selenobrachys philippinus* comb. rest.	13.43%	13.43%	13.43%	13.43%	13.43%	13.43%	13.19%	13.42%	13.53%	13.74%	13.74%	13.74%	13.74%	13.74%
TRT-002 *Selenobrachys ustromsupasius* sp. nov.	12.36%	11.95%	12.36%	11.95%	11.95%	11.95%	10.58%	10.79%	11.07%	11.07%	11.07%	11.07%	11.07%	11.07%
TRT-001 *Selenobrachys ustromsupasius* sp. nov.	12.33%	11.92%	12.33%	11.92%	11.92%	11.92%	10.35%	10.57%	11.70%	11.24%	11.24%	11.24%	11.24%	11.24%
TRT-003 *Selenobrachys ustromsupasius* sp. nov.	12.39%	12.18%	12.39%	12.18%	12.18%	12.18%	10.80%	11.02%	11.30%	11.30%	11.30%	11.30%	11.30%	11.30%
R01-05 *Selenobrachys ustromsupasius* sp. nov.	12.56%	12.15%	12.56%	12.15%	12.15%	12.15%	10.59%	10.81%	11.49%	11.49%	11.49%	11.49%	11.49%	11.49%
R01-09 *Selenobrachys ustromsupasius* sp. nov.	12.15%	11.74%	12.15%	11.74%	11.74%	11.74%	10.78%	11.00%	11.27%	11.27%	11.27%	11.27%	11.27%	11.27%
R02-02 *Selenobrachys ustromsupasius* sp. nov.	12.56%	12.15%	12.56%	12.15%	12.15%	12.15%	10.58%	10.79%	11.94%	11.47%	11.47%	11.47%	11.47%	11.47%
SMNS Aran-004163 *Chilocosmia dichromata* comb. rest.	15.28%	14.63%	15.28%	14.63%	14.63%	14.63%	12.50%	12.85%	11.27%	13.29%	13.29%	13.29%	13.29%	13.29%
SMNS Aran-004164 *Chilocosmia dichromata* comb. rest.	15.28%	14.63%	15.28%	14.63%	14.63%	14.63%	12.50%	12.85%	11.27%	13.29%	13.29%	13.29%	13.29%	13.29%
SMNS Aran-004185 *Chilocosmia dichromata* comb. rest.	15.93%	15.28%	15.93%	15.28%	15.28%	15.28%	11.86%	12.21%	10.93%	12.94%	12.94%	12.94%	12.94%	12.94%
SMNS Aran-004162 *Chilocosmia dichromata* comb. rest.	15.28%	14.63%	15.28%	14.63%	14.63%	14.63%	12.50%	12.85%	11.27%	13.29%	13.29%	13.29%	13.29%	13.29%
SMNS Aran-004184 *Chilocosmia dichromata* comb. rest.	15.93%	15.28%	15.93%	15.28%	15.28%	15.28%	11.52%	11.86%	10.59%	12.59%	12.59%	12.59%	12.59%	12.59%
KJ744742 *Selenotholus* sp.	14.68%	14.68%	14.68%	14.68%	14.68%	14.68%	12.99%	13.22%	14.36%	15.42%	15.42%	15.42%	15.42%	15.42%
JF884459 *Chilobrachys* sp.	13.11%	12.90%	13.11%	12.90%	12.90%	12.90%	13.88%	14.12%	14.37%	14.09%	14.09%	14.09%	14.09%	14.09%
MF804598 *Chilobrachys* sp.	14.91%	14.91%	14.91%	14.91%	14.91%	14.91%	12.63%	12.86%	12.82%	13.30%	13.30%	13.30%	13.30%	13.30%
JN018125 *Chilobrachys huahini*	14.46%	14.04%	14.46%	14.04%	14.04%	14.04%	13.02%	13.25%	13.12%	12.14%	12.14%	12.14%	12.14%	12.14%
JN018124 *Aphonopelma seemanni*	16.96%	16.74%	16.96%	16.74%	16.74%	16.74%	16.61%	16.86%	15.69%	15.94%	15.94%	15.94%	15.94%	15.94%
MK234708 *Grammostola rosea*	16.63%	16.41%	16.63%	16.41%	16.41%	16.41%	13.70%	13.93%	14.02%	14.45%	14.45%	14.45%	14.45%	14.45%
KT022078 *Grammostola* sp.	15.41%	15.41%	15.41%	15.41%	15.41%	15.41%	14.29%	14.52%	15.08%	15.73%	15.73%	15.73%	15.73%	15.73%
KT995328 *Brachypelma verdezi*	15.58%	15.79%	15.58%	15.79%	15.79%	15.79%	16.57%	16.82%	15.78%	17.25%	17.25%	17.25%	17.25%	17.25%

	QR1-01	QR1-02	LS02-02	LS04-09	LS04-11	LS04-12	LS03-01	DME22A001.1	NEPT1-003	DCA 37	CSL02-01	CSL04-02	CSL04-01	CSL04-03	CSL04-04	CSL04-05	CSL06-02	PBAT1-01	NOM1A-01	NOM1A-04
QR1-02 *Orphnaecus* sp. 'L2'	0.00%																			
LS02-02 *Orphnaecus* sp. 'L2'	0.87%	0.87%																		
LS04-09 *Orphnaecus* sp. 'L2'	0.35%	0.35%	1.23%																	
LS04-11 *Orphnaecus* sp. 'L2'	0.60%	0.60%	1.42%	0.20%																
LS04-12 *Orphnaecus* sp. 'L2'	0.35%	0.35%	1.23%	0.00%	0.20%															
LS03-01 *Orphnaecus* sp. 'L2'	0.87%	0.87%	0.00%	1.23%	1.42%	1.23%														
DME22A001.1 *Orphnaecus* sp. 'L5'	6.72%	6.72%	6.12%	6.33%	6.94%	6.33%	6.12%													
NEPT1-003 *Orphnaecus* sp. 'L5'	6.52%	6.52%	5.91%	6.12%	6.70%	6.12%	5.91%	3.38%												
DCA 37 *Orphnaecus* sp. 'L6'	7.92%	7.92%	8.34%	7.52%	8.06%	7.52%	8.34%	7.07%	6.48%											
CSL02-01 *Orphnaecus* sp. 'L3'	10.54%	10.54%	10.74%	10.12%	10.16%	10.12%	10.74%	8.89%	8.88%	8.53%										
CSL04-02 *Orphnaecus* sp. 'L3'	10.54%	10.54%	10.74%	10.12%	10.16%	10.12%	10.74%	8.89%	8.88%	8.53%	0.00%									
CSL04-01 *Orphnaecus* sp. 'L3'	10.54%	10.54%	10.74%	10.12%	10.16%	10.12%	10.74%	8.89%	8.88%	8.53%	0.00%	0.00%								
CSL04-03 *Orphnaecus* sp. 'L3'	10.54%	10.54%	10.74%	10.12%	10.16%	10.12%	10.74%	8.89%	8.88%	8.53%	0.00%	0.00%	0.00%							
CSL04-04 *Orphnaecus* sp. 'L3'	10.54%	10.54%	10.74%	10.12%	10.16%	10.12%	10.74%	8.89%	8.88%	8.53%	0.00%	0.00%	0.00%	0.00%						
CSL04-05 *Orphnaecus* sp. 'L3'	10.54%	10.54%	10.74%	10.12%	10.16%	10.12%	10.74%	8.89%	8.88%	8.53%	0.00%	0.00%	0.00%	0.00%	0.00%					
CSL06-02 *Orphnaecus* sp. 'L4'	7.87%	7.87%	7.87%	7.85%	7.51%	7.85%	7.87%	8.49%	8.47%	7.34%	8.09%	8.09%	8.09%	8.09%	8.09%	8.09%				
PBAT1-01 *Orphnaecus* sp. 'M1'	8.53%	8.53%	8.73%	8.52%	8.78%	8.52%	8.73%	8.10%	8.90%	7.16%	5.29%	5.29%	5.29%	5.29%	5.29%	5.29%	6.69%			
NOM1A-01 *Selenobrachys philippinus* comb. rest.	13.29%	13.29%	13.64%	12.86%	12.81%	12.86%	13.64%	11.67%	12.33%	15.04%	11.93%	11.93%	11.93%	11.93%	11.93%	11.93%	13.20%	11.63%		
NOM1A-04 *Selenobrachys philippinus* comb. rest.	13.75%	13.75%	14.10%	13.31%	13.33%	13.31%	14.10%	12.12%	12.78%	15.50%	12.38%	12.38%	12.38%	12.38%	12.38%	12.38%	13.65%	12.07%	0.35%	
TRT-002 *Selenobrachys ustromsupasius* sp. nov.	11.33%	11.33%	11.08%	10.91%	11.06%	10.91%	11.08%	10.35%	10.58%	12.84%	12.72%	12.72%	12.72%	12.72%	12.72%	12.72%	10.23%	11.47%	8.43%	8.85%
TRT-001 *Selenobrachys ustromsupasius* sp. nov.	11.10%	11.10%	11.31%	11.09%	11.03%	11.09%	11.31%	10.98%	10.60%	13.01%	12.69%	12.69%	12.69%	12.69%	12.69%	12.69%	10.20%	11.04%	8.46%	8.87%
TRT-003 *Selenobrachys ustromsupasius* sp. nov.	11.57%	11.57%	11.31%	11.14%	11.33%	11.14%	11.31%	10.58%	10.60%	12.87%	12.75%	12.75%	12.75%	12.75%	12.75%	12.75%	10.25%	11.70%	8.46%	8.87%
R01-05 *Selenobrachys ustromsupasius* sp. nov.	11.76%	11.76%	11.50%	11.33%	11.55%	11.33%	11.50%	10.77%	10.99%	13.27%	13.15%	13.15%	13.15%	13.15%	13.15%	13.15%	10.43%	11.90%	8.84%	9.26%
R01-09 *Selenobrachys ustromsupasius* sp. nov.	11.54%	11.54%	11.28%	11.11%	11.29%	11.11%	11.28%	10.55%	10.38%	12.63%	12.93%	12.93%	12.93%	12.93%	12.93%	12.93%	10.43%	11.68%	8.63%	9.05%
R02-02 *Selenobrachys ustromsupasius* sp. nov.	11.33%	11.33%	11.54%	11.32%	11.29%	11.32%	11.54%	11.21%	10.83%	13.25%	12.93%	12.93%	12.93%	12.93%	12.93%	12.93%	10.43%	11.27%	8.67%	9.09%
SMNS Aran-004163 *Chilocosmia dichromata* comb. rest.	14.35%	14.35%	14.30%	14.35%	16.35%	14.35%	14.30%	13.26%	13.23%	13.70%	14.93%	14.93%	14.93%	14.93%	14.93%	14.93%	14.93%	14.62%	17.36%	18.11%
SMNS Aran-004164 *Chilocosmia dichromata* comb. rest.	14.35%	14.35%	14.30%	14.35%	16.35%	14.35%	14.30%	13.26%	13.23%	13.70%	14.93%	14.93%	14.93%	14.93%	14.93%	14.93%	14.93%	14.62%	17.36%	18.11%
SMNS Aran-004185 *Chilocosmia dichromata* comb. rest.	13.67%	13.67%	13.62%	13.67%	15.93%	13.67%	13.62%	12.62%	13.23%	13.70%	14.93%	14.93%	14.93%	14.93%	14.93%	14.93%	12.90%	13.26%	16.34%	17.07%
SMNS Aran-004162 *Chilocosmia dichromata* comb. rest.	14.68%	14.68%	14.63%	14.68%	16.76%	14.68%	14.63%	13.26%	13.23%	13.70%	14.93%	14.93%	14.93%	14.93%	14.93%	14.93%	14.61%	14.62%	17.36%	18.11%
SMNS Aran-004184 *Chilocosmia dichromata* comb. rest.	13.67%	13.67%	13.62%	13.67%	15.93%	13.67%	13.62%	12.27%	12.88%	13.34%	14.58%	14.58%	14.58%	14.58%	14.58%	14.58%	13.25%	13.62%	16.70%	17.44%
KJ744742 *Selenotholus* sp.	14.77%	14.77%	15.15%	14.33%	14.29%	14.33%	15.15%	14.07%	14.06%	14.38%	14.16%	14.16%	14.16%	14.16%	14.16%	14.16%	14.17%	14.55%	15.00%	15.47%
JF884459 *Chilobrachys* sp.	15.02%	15.02%	14.13%	14.78%	14.94%	14.78%	14.13%	13.49%	13.29%	13.99%	13.96%	13.96%	13.96%	13.96%	13.96%	13.96%	13.76%	15.05%	15.35%	15.82%
MF804598 *Chilobrachys* sp.	14.63%	14.63%	14.35%	14.61%	15.85%	14.61%	14.35%	13.69%	13.96%	13.32%	14.09%	14.09%	14.09%	14.09%	14.09%	14.09%	13.35%	13.72%	18.03%	18.52%
JN018125 *Chilobrachys huahini*	14.76%	14.76%	14.69%	14.52%	14.45%	14.52%	14.69%	13.89%	14.09%	15.69%	12.82%	12.82%	12.82%	12.82%	12.82%	12.82%	13.56%	13.86%	16.27%	16.75%
JN018124 *Aphonopelma seemanni*	15.18%	15.18%	14.71%	15.40%	14.83%	15.40%	14.71%	14.75%	14.10%	16.94%	18.98%	18.98%	18.98%	18.98%	18.98%	18.98%	16.03%	17.64%	19.01%	19.51%
MK234708 *Grammostola rosea*	15.31%	15.31%	14.86%	15.55%	16.95%	15.55%	14.86%	14.03%	14.17%	16.57%	15.29%	15.29%	15.29%	15.29%	15.29%	15.29%	14.78%	15.80%	17.50%	17.99%
KT022078 *Grammostola* sp.	16.51%	16.51%	16.05%	16.27%	17.50%	16.27%	16.05%	14.20%	13.76%	15.94%	15.46%	15.46%	15.46%	15.46%	15.46%	15.46%	14.59%	16.18%	16.34%	16.82%
KT995328 *Brachypelma verdezi*	16.75%	16.75%	16.46%	16.73%	17.24%	16.73%	16.46%	16.22%	16.64%	15.82%	17.37%	17.37%	17.37%	17.37%	17.37%	17.37%	16.56%	17.60%	17.88%	18.38%

	TRT-002	TRT-001	TRT-003	R01-05	R01-09	R02-02	SMNS Aran-004163	SMNS Aran-004164	SMNS Aran-004185	SMNS Aran-004162	SMNS Aran-004184	KJ744742	JF884459	MF804598	JN018125	JN018124	MK234708	KT022078	KT995328
TRT-001 *Selenobrachys ustromsupasius* sp. nov.	0.88%																		
TRT-003 *Selenobrachys ustromsupasius* sp. nov.	0.17%	1.05%																	
R01-05 *Selenobrachys ustromsupasius* sp. nov.	0.35%	1.23%	0.52%																
R01-09 *Selenobrachys ustromsupasius* sp. nov.	0.17%	1.05%	0.35%	0.52%															
R02-02 *Selenobrachys ustromsupasius* sp. nov.	0.70%	0.17%	0.88%	1.05%	0.87%														
SMNS Aran-004163 *Chilocosmia dichromata* comb. rest.	12.84%	12.79%	13.20%	13.19%	12.52%	13.16%													
SMNS Aran-004164 *Chilocosmia dichromata* comb. rest.	12.84%	12.79%	13.20%	13.19%	12.52%	13.16%	0.00%												
SMNS Aran-004185 *Chilocosmia dichromata* comb. rest.	12.20%	12.15%	12.56%	12.54%	12.52%	12.52%	2.80%	2.80%											
SMNS Aran-004162 *Chilocosmia dichromata* comb. rest.	12.84%	12.79%	13.20%	13.19%	12.52%	13.16%	0.19%	0.19%	2.59%										
SMNS Aran-004184 *Chilocosmia dichromata* comb. rest.	11.85%	11.81%	12.21%	12.20%	12.17%	12.17%	2.80%	2.80%	0.39%	2.59%									
KJ744742 *Selenotholus* sp.	15.90%	15.41%	15.93%	15.92%	15.68%	15.66%	14.35%	14.35%	14.00%	14.35%	13.64%								
JF884459 *Chilobrachys* sp.	15.67%	15.69%	15.42%	15.89%	15.46%	15.94%	18.28%	18.28%	17.53%	18.62%	17.16%	15.45%							
MF804598 *Chilobrachys* sp.	16.00%	16.25%	16.25%	16.02%	15.78%	16.50%	14.64%	14.64%	13.54%	14.64%	13.90%	15.59%	13.68%						
JN018125 *Chilobrachys huahini*	14.09%	14.55%	14.33%	13.88%	14.30%	14.79%	17.80%	17.80%	17.09%	17.80%	16.71%	16.86%	13.44%	12.11%					
JN018124 *Aphonopelma seemanni*	16.67%	16.53%	16.41%	16.69%	16.88%	16.28%	18.66%	18.66%	16.87%	18.31%	16.50%	17.11%	17.14%	15.94%	17.97%				
MK234708 *Grammostola rosea*	14.54%	14.50%	14.29%	14.32%	14.32%	14.75%	15.69%	15.69%	15.32%	15.69%	14.95%	17.56%	15.09%	16.03%	15.87%	16.36%			
KT022078 *Grammostola* sp.	14.04%	14.01%	14.08%	13.83%	13.83%	14.26%	15.65%	15.65%	15.28%	15.65%	14.92%	17.39%	14.66%	17.12%	16.44%	17.32%	5.66%		
KT995328 *Brachypelma verdezi*	16.04%	16.37%	15.79%	15.83%	15.83%	16.12%	17.03%	17.03%	16.66%	16.69%	16.29%	16.70%	16.69%	15.17%	16.37%	13.53%	16.52%	17.97%	

Supplementary data 2.2. Percent pairwise distances between 12S–tRNA-Val–16S sequences

	CSL01-08	CSL01-02	CSL01-01	CSL05-07	CSL05-06	CSL05-05	CSL05-01	QBI-10	QBI-07	QBI-03	MG273471-1	UPLG1-03	UPLG1-01	QRI-02	LS04-08	DME22A001.1
CSL01-08 *Orphnaecus pellitus*																
CSL01-02 *Orphnaecus pellitus*	0.25%															
CSL01-01 *Orphnaecus pellitus*	0.50%	0.25%														
CSL05-07 *Orphnaecus pellitus*	0.00%	0.25%	0.50%													
CSL05-06 *Orphnaecus pellitus*	0.00%	0.25%	0.50%	0.00%												
CSL05-05 *Orphnaecus pellitus*	0.00%	0.25%	0.50%	0.00%	0.00%											
CSL05-01 *Orphnaecus pellitus*	0.00%	0.25%	0.50%	0.00%	0.00%	0.00%										
QBI-10 *Orphnaecus kwebaburdeos*	11.40%	11.77%	12.13%	11.40%	11.40%	11.40%	11.40%									
QBI-07 *Orphnaecus kwebaburdeos*	11.40%	11.77%	12.13%	11.40%	11.40%	11.40%	11.40%	0.00%								
QBI-03 *Orphnaecus kwebaburdeos*	11.40%	11.77%	12.13%	11.40%	11.40%	11.40%	11.40%	0.00%	0.00%							
MG273471-1-*Orphnaeus* [*Orphnaecus*] sp. 'N1'	11.09%	11.46%	11.81%	11.09%	11.09%	11.09%	11.09%	9.67%	9.67%	9.67%						
UPLG1-03 *Orphnaecus* sp. 'L1'	8.47%	8.81%	9.13%	8.47%	8.47%	8.47%	8.47%	12.19%	12.19%	12.19%	11.01%					
UPLG1-01 *Orphnaecus* sp. 'L1'	8.79%	9.13%	9.46%	8.79%	8.79%	8.79%	8.79%	12.55%	12.55%	12.55%	11.35%	0.25%				
QRI-02 *Orphnaecus* sp. 'L2'	12.03%	12.40%	12.76%	12.03%	12.03%	12.03%	12.03%	11.54%	11.54%	11.54%	11.33%	9.72%	10.05%			
LS04-08 *Orphnaecus* sp. 'L2'	12.03%	12.40%	12.76%	12.03%	12.03%	12.03%	12.03%	11.54%	11.54%	11.54%	11.33%	9.72%	10.05%	0.00%		
DME22A001.1 *Orphnaecus* sp. 'L5'	10.06%	10.41%	10.75%	10.06%	10.06%	10.06%	10.06%	10.02%	10.02%	10.02%	10.57%	9.32%	9.65%	6.81%	6.81%	
CSL02-02 *Orphnaecus* sp. 'L3'	12.80%	13.18%	13.54%	12.80%	12.80%	12.80%	12.80%	4.98%	4.98%	4.98%	8.87%	11.68%	12.03%	10.08%	10.08%	9.05%
CSL02-01 *Orphnaecus* sp. 'L3'	12.80%	13.18%	13.54%	12.80%	12.80%	12.80%	12.80%	4.98%	4.98%	4.98%	8.87%	11.68%	12.03%	10.08%	10.08%	9.05%
CSL04-03 *Orphnaecus* sp. 'L3'	12.80%	13.18%	13.54%	12.80%	12.80%	12.80%	12.80%	4.98%	4.98%	4.98%	8.87%	11.68%	12.03%	10.08%	10.08%	9.05%
CSL04-02 *Orphnaecus* sp. 'L3'	12.80%	13.18%	13.54%	12.80%	12.80%	12.80%	12.80%	4.98%	4.98%	4.98%	8.87%	11.68%	12.03%	10.08%	10.08%	9.05%
CSL04-01 *Orphnaecus* sp. 'L3'	12.80%	13.18%	13.54%	12.80%	12.80%	12.80%	12.80%	4.98%	4.98%	4.98%	8.87%	11.68%	12.03%	10.08%	10.08%	9.05%
NOM1A-01 *Selenobrachys philippinus* comb. rest.	14.06%	14.46%	14.84%	14.06%	14.06%	14.06%	14.06%	16.01%	16.01%	16.01%	14.26%	14.89%	15.27%	15.16%	15.16%	17.17%
NOM1A-04 *Selenobrachys philippinus* comb. rest.	14.06%	14.46%	14.84%	14.06%	14.06%	14.06%	14.06%	16.01%	16.01%	16.01%	14.26%	14.89%	15.27%	15.16%	15.16%	17.17%
NOM1A-07 *Selenobrachys philippinus* comb. rest.	14.84%	15.24%	15.63%	14.84%	14.84%	14.84%	14.84%	16.81%	16.81%	16.81%	15.03%	15.69%	16.08%	15.95%	15.95%	17.99%
R02-16 *Selenobrachys ustromsupasius* sp. nov.	12.05%	12.42%	12.77%	12.05%	12.05%	12.05%	12.05%	13.88%	13.88%	13.88%	12.80%	12.90%	13.26%	14.63%	14.63%	14.15%
R01-01 *Selenobrachys ustromsupasius* sp. nov.	12.05%	12.42%	12.77%	12.05%	12.05%	12.05%	12.05%	13.88%	13.88%	13.88%	12.80%	12.90%	13.26%	14.63%	14.63%	14.15%
R01-02 *Selenobrachys ustromsupasius* sp. nov.	12.05%	12.42%	12.77%	12.05%	12.05%	12.05%	12.05%	13.88%	13.88%	13.88%	12.80%	12.90%	13.26%	14.63%	14.63%	14.15%
R02-02 *Selenobrachys ustromsupasius* sp. nov.	12.05%	12.42%	12.77%	12.05%	12.05%	12.05%	12.05%	13.88%	13.88%	13.88%	12.80%	12.90%	13.26%	14.63%	14.63%	14.15%
MG273467-1-*Chilobrachys-fimbriatus*	24.07%	24.57%	25.03%	24.07%	24.07%	24.07%	24.07%	22.70%	22.70%	22.70%	21.74%	21.83%	22.27%	26.60%	26.60%	21.62%
MG273477-1-*Phlogiellus* [?]	26.57%	27.12%	27.61%	26.57%	26.57%	26.57%	26.57%	25.26%	25.26%	25.26%	21.87%	22.74%	23.19%	24.16%	24.16%	22.68%
MG273476-1-*Lyrognathus-giannisposatoi*	26.39%	26.93%	27.41%	26.39%	26.39%	26.39%	26.39%	27.60%	27.60%	27.60%	24.00%	27.06%	27.55%	24.79%	24.79%	26.00%
MG273480-1-*Selenocosmia-javanensis*	32.13%	32.73%	33.27%	32.13%	32.13%	32.13%	32.13%	29.27%	29.27%	29.27%	30.08%	29.51%	30.02%	31.41%	31.41%	31.13%
MG273468-1-*Cyriopagopus-lividus*	34.39%	35.03%	35.60%	34.39%	34.39%	34.39%	34.39%	37.43%	37.43%	37.43%	36.69%	34.92%	34.92%	35.04%	35.04%	34.66%
MG273484-1-*Poecilotheria-formosa*	40.06%	40.76%	41.37%	40.06%	40.06%	40.06%	40.06%	35.30%	35.30%	35.30%	34.94%	33.85%	34.40%	32.22%	32.22%	35.31%
MG273470-1-*Lampropelma-nigerrimum*	38.56%	39.24%	39.84%	38.56%	38.56%	38.56%	38.56%	37.65%	37.65%	37.65%	34.43%	38.17%	38.77%	36.96%	36.96%	34.84%
MG273466-1-*Brachypelma* sp.	38.27%	38.96%	39.57%	38.27%	38.27%	38.27%	38.27%	37.12%	37.12%	37.12%	36.96%	39.14%	39.14%	38.98%	38.98%	36.84%
MG273485-1-*Grammostola-pulchripes*	36.45%	37.11%	37.70%	36.45%	36.45%	36.45%	36.45%	38.73%	38.73%	38.73%	36.49%	39.24%	39.24%	38.07%	38.07%	38.13%
AS1 *Aphonopelma seemanni*	39.90%	40.61%	41.23%	39.90%	39.90%	39.90%	39.90%	32.76%	32.76%	32.76%	37.51%	40.98%	40.98%	37.04%	37.04%	36.41%

	CSL02-02	CSL02-01	CSL04-03	CSL04-02	CSL04-01	NOM1A-01	NOM1A-04	NOM1A-07	R02-16	R01-01	R01-02	R02-02	MG273467-1	MG273477-1	MG273476-1	MG273480-1	MG273468-1	MG273484-1	MG273470-1	MG273466-1	MG273485-1	AS1
CSL02-01 *Orphnaecus* sp. 'L3'	0.00%																					
CSL04-03 *Orphnaecus* sp. 'L3'	0.00%	0.00%																				
CSL04-02 *Orphnaecus* sp. 'L3'	0.00%	0.00%	0.00%																			
CSL04-01 *Orphnaecus* sp. 'L3'	0.00%	0.00%	0.00%	0.00%																		
NOM1A-01 *Selenobrachys philippinus* comb. rest.	17.50%	17.50%	17.50%	17.50%	17.50%																	
NOM1A-04 *Selenobrachys philippinus* comb. rest.	17.50%	17.50%	17.50%	17.50%	17.50%	0.00%																
NOM1A-07 *Selenobrachys philippinus* comb. rest.	18.33%	18.33%	18.33%	18.33%	18.33%	0.50%	0.50%															
R02-16 *Selenobrachys ustromsupasius* sp. nov.	16.47%	16.47%	16.47%	16.47%	16.47%	9.16%	9.16%	9.82%														
R01-01 *Selenobrachys ustromsupasius* sp. nov.	16.47%	16.47%	16.47%	16.47%	16.47%	9.16%	9.16%	9.82%	0.00%													
R01-02 *Selenobrachys ustromsupasius* sp. nov.	16.47%	16.47%	16.47%	16.47%	16.47%	9.16%	9.16%	9.82%	0.00%	0.00%												
R02-02 *Selenobrachys ustromsupasius* sp. nov.	16.47%	16.47%	16.47%	16.47%	16.47%	9.16%	9.16%	9.82%	0.00%	0.00%	0.00%											
MG273467-1-*Chilobrachys-fimbriatus*	20.11%	20.11%	20.11%	20.11%	20.11%	25.75%	25.75%	26.76%	28.77%	28.77%	28.77%	28.77%										
MG273477-1-*Phlogiellus* [?]	25.38%	25.38%	25.38%	25.38%	25.38%	20.29%	20.29%	21.20%	23.89%	23.89%	23.89%	23.89%	27.63%									
MG273476-1-*Lyrognathus-giannisposatoi*	28.32%	28.32%	28.32%	28.32%	28.32%	24.11%	24.11%	25.08%	25.35%	25.35%	25.35%	25.35%	26.87%	28.41%								
MG273480-1-*Selenocosmia-javanensis*	31.30%	31.30%	31.30%	31.30%	31.30%	31.87%	31.87%	33.00%	31.13%	31.13%	31.13%	31.13%	28.64%	31.25%	17.88%							
MG273468-1-*Cyriopagopus-lividus*	38.53%	38.53%	38.53%	38.53%	38.53%	38.46%	38.46%	39.75%	35.51%	35.51%	35.51%	35.51%	39.21%	40.04%	44.98%	44.57%						
MG273484-1-*Poecilotheria-formosa*	35.31%	35.31%	35.31%	35.31%	35.31%	33.40%	33.40%	34.57%	31.25%	31.25%	31.25%	31.25%	38.93%	40.10%	43.19%	45.43%	24.09%					
MG273470-1-*Lampropelma-nigerrimum*	39.73%	39.73%	39.73%	39.73%	39.73%	33.85%	33.85%	35.02%	38.03%	38.03%	38.03%	38.03%	37.63%	35.91%	39.93%	47.64%	23.12%	32.00%				
MG273466-1-*Brachypelma* sp.	37.88%	37.88%	37.88%	37.88%	37.88%	41.42%	41.42%	42.78%	41.05%	41.05%	41.05%	41.05%	35.38%	45.70%	37.96%	48.59%	33.94%	37.79%	38.63%			
MG273485-1-*Grammostola-pulchripes*	38.65%	38.65%	38.65%	38.65%	38.65%	37.35%	37.35%	38.61%	35.47%	35.47%	35.47%	35.47%	34.37%	38.24%	39.92%	43.78%	31.31%	35.50%	35.96%	25.31%		
AS1 *Aphonopelma seemanni*	35.54%	35.54%	35.54%	35.54%	35.54%	37.78%	37.78%	39.06%	36.00%	36.00%	36.00%	36.00%	39.35%	37.33%	38.92%	48.43%	40.76%	37.24%	33.94%	21.58%	31.95%	
